# WFX Molecular Fragment References for Hirshfeld Charge‐Transfer Analysis in Non‐Covalent Complexes

**DOI:** 10.1002/jcc.70476

**Published:** 2026-07-29

**Authors:** Jorge Garza, Rubicelia Vargas

**Affiliations:** ^1^ Departamento de Química, División de Ciencias Básicas e Ingeniería Universidad Autónoma Metropolitana Iztapalapa Iztapalapa México

**Keywords:** charge transfer, fragments, Hirshfeld, non‐covalent interactions

## Abstract

In this work, we introduce and assess a fragment‐based Hirshfeld scheme in which molecular WFX files of isolated fragments are used to reconstruct reference densities directly. The electron density of the full complex is reconstructed from its WFX file and partitioned using either atomic WFX references or molecular WFX fragment references on the same integration grid. The method is applied to water clusters and to representative complexes from the S66 set, including hydrogen‐bonded, donor–acceptor, π‐stacked, symmetric, and dispersion‐dominated systems. Across density‐functional calculations and the available CCSD correlated‐wave‐ function results, molecular WFX fragment references lead to substantially smaller fragment‐charge magnitudes than atomic references. For hydrogen‐bonded and donor–acceptor complexes, the reduction commonly reaches 75%–90% in the primary 6‐311++G** calculations, whereas symmetric or dispersion‐dominated systems exhibit zero or nearly zero net integrated fragment charge. The results show that atomic‐reference fragment charges include contributions associated with reconstructing each molecular fragment from isolated atoms, whereas WFX fragment references measure redistribution relative to already formed isolated monomers. The proposed approach therefore provides a chemically meaningful density‐based reference for analyzing intermolecular charge transfer in non‐covalent complexes.

## Graphical abstract

The choice of reference state strongly affects Hirshfeld charge‐transfer analysis. Atomic references predict substantially larger intermolecular charge transfer than molecular fragment WFX references in non‐covalent complexes.

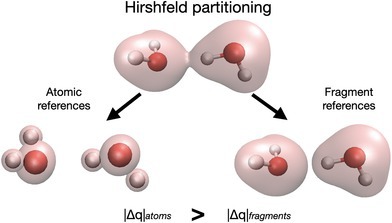

## 1 Introduction

Non‐covalent interactions play a central role in molecular recognition, supramolecular organization, hydrogen‐bond networks, biomolecular structure, molecular crystals, and host–guest chemistry [1, 2]. In the terminology of the IUPAC Gold Book, these interactions include hydrogen bonding, electrostatic interactions, and van der Waals forces that contribute to the stabilization of molecular and biomolecular structures [3]. Although non‐covalent interactions are usually weaker than covalent bonds when considered individually, their cumulative effect often determines the structure, stability, and properties of complex chemical systems. Their physical origin is multifaceted and involves electrostatics, exchange repulsion, induction, dispersion, and, in some cases, charge‐transfer contributions [4]. A reliable description of non‐covalent complexes therefore requires not only the calculation of interaction energies, but also an analysis of the accompanying electronic‐density redistribution [5].

Charge redistribution in non‐covalent complexes is commonly discussed in terms of polarization, induction, and charge transfer. In particular, intermolecular charge transfer provides a chemically useful way to identify donor–acceptor effects and directional electronic communication between molecular units [6, 7]. However, the quantitative evaluation of charge transfer is not unique, because it depends on the reference state and on the partitioning scheme used to assign the molecular electron density to different parts of the system. This point is especially important for weakly bound complexes, where the expected net charge transfer may be small and can be affected by how the interacting units are defined [8, 9, 10, 11, 12].

The concept of a molecular fragment is therefore essential. In order to discuss charge transfer, one must first define the chemical units between which the transfer is evaluated. For a water dimer, a water cluster, a hydrogen‐bonded donor–acceptor complex, or a stacked aromatic system, the chemically relevant units are usually the molecular monomers or selected functional groups rather than isolated atoms. Thus, the fragment definition is not a merely technical detail; it establishes the physical meaning of the population analysis and determines the reference with respect to which charge redistribution is interpreted. More general stockholder formulations are not intrinsically restricted to atomic reference entities, but may be expressed in terms of arbitrary density pieces or molecular fragments, as emphasized in generalized and variational Hirshfeld frameworks [13].

Fragment concepts are widely used in computational chemistry, including fragment‐localized orbital frameworks, energy‐decomposition analyses, and other approaches in which chemically meaningful molecular units are used to interpret or simplify the electronic structure of large systems [7, 8]. In these contexts, fragments provide a way to reduce the complexity of molecular systems while retaining the identity of the interacting units. The same idea is natural for the analysis of charge transfer in non‐covalent complexes: the charge assigned to a fragment should ideally be measured relative to an appropriate reference for that fragment.

Hirshfeld population analysis provides an elegant density‐based framework for assigning electron populations through stockholder weights. In the conventional atomic formulation, the promolecular density is built from isolated spherical atomic reference densities [14, 15, 16]. This definition is well established and leads to useful atomic charges. Nevertheless, when the target property is the net charge transferred between molecular units, an atomic promolecule may not be the most direct reference [17]. If a molecular fragment is represented only as a sum of isolated atoms, the resulting fragment charge may contain not only intermolecular charge redistribution, but also contributions associated with the reconstruction of the intramolecular density of each monomer from atomic densities.

Previous charge‐transfer analyses based on donor–acceptor orbital descriptions, fragment‐localized orbital frameworks, and energy‐decomposition schemes have emphasized the importance of separating charge transfer from other components of the interaction [6, 7, 9, 10, 11]. At the same time, critical discussions of orbital‐based interpretations have shown that the physical meaning assigned to charge transfer can depend strongly on the chosen reference and analysis procedure [18]. The present work addresses a complementary density‐based problem. Rather than decomposing the interaction energy, we examine how the reference density used in Hirshfeld population analysis affects the resulting fragment charges.

In this work, we introduce and assess a fragment‐based Hirshfeld scheme in which molecular fragments, rather than isolated atoms alone, are used as reference entities for constructing the promolecular density. The molecular electron density of the full complex is retained, but the Hirshfeld denominator can be built either from spherical atomic WFX densities or from molecular WFX densities corresponding to preformed isolated fragments. This allows atomic‐reference fragment charges and molecular‐fragment‐reference charges to be compared on the same numerical footing. This provides a direct WFX‐based route to use preformed molecular fragments, rather than isolated atoms alone, as reference entities in Hirshfeld charge‐transfer analysis. The method is evaluated using water‐cluster geometries reported by Miliordos, Aprà, and Xantheas [19] and representative complexes from the S66 set [20], with particular attention to the difference between fragment charges derived from atomic references and those obtained from molecular WFX fragment references. The WFX files were generated with Gaussian 09 [21].

## 2 Methodology

### 2.1 Hirshfeld Population Analysis From WFX Wave Functions

Hirshfeld populations and charges were evaluated directly from wave‐function data stored in WFX format. The molecular electron density of the full system, $\rho \left(r\right)$, was reconstructed from the molecular WFX file, which also provided the nuclear coordinates used in the numerical integration. The WFX‐reading and density‐evaluation modules were adapted from the Graphics Processing Units for Atoms and Molecules (GPUAM) project [22, 23]. The Hirshfeld population assigned to a reference entity X was obtained as

(1) $$N_{X}=\int h_{X}\left(r\right)\;\rho \left(r\right)\;dr,$$

where the Hirshfeld weight is defined as

(2) $$h_{X}\left(r\right)=\frac{\rho_{X}^{\mathrm{ref}}\left(r\right)}{\sum_{Y}\;\rho_{Y}^{\mathrm{ref}}\left(r\right)}.$$

The corresponding Hirshfeld charge was computed as

(3) $$q_{X}=Z_{X}^{\mathrm{ref}}-N_{X}.$$

In this equation, $Z_{X}^{\mathrm{ref}}$ is the sum of the nuclear charges associated with the atoms assigned to entity X. For atomic entities, $Z_{X}^{\mathrm{ref}}$ is simply the nuclear charge of the atom, whereas for molecular fragments it is the sum of the nuclear charges of the atoms belonging to the fragment.

Numerical integrations were carried out on a multicenter Becke grid centered on the nuclei of the full system. The radial coordinate was mapped according to $r=-\alpha \ln\left(1-x^{m}\right)$ [24], where x is equally spaced from 0 to $1-1/N_{r}$ and it never touches 1. Angular integrations were performed using a Gauss–Legendre quadrature in μ=cosθ and an equally spaced quadrature in the azimuthal angle. The Becke partition weights were used only to define the multicenter numerical integration grid [25, 26], whereas the Hirshfeld weights were built from the selected reference densities. Unless otherwise stated, all calculations used $N_{r}=100$, $N_{\mu }=24$, $N_{\phi }=48$, α=7.0, and m=4.0. The implementation monitors both the normalization of the Becke partition and the local sum of the Hirshfeld weights.

### 2.2 Atomic WFX–Based Hirshfeld Charges

In the first implementation, conventional atomic Hirshfeld charges were obtained. The promolecular density was constructed as a sum of spherical atomic reference densities,

(4) $$\rho_{\text{promol}}\left(r\right)=\sum_{A}\;\rho_{A}^{\mathrm{sph}}\left(|r-R_{A}|\right).$$

Each spherical atomic density was generated from an isolated atomic WFX file. The atomic density was evaluated on radial shells and spherically averaged over angular directions. Linear interpolation was then used to evaluate the spherical density at arbitrary grid points during the Becke‐grid integration. The Hirshfeld population of atom A was therefore computed using

(5) $$h_{A}\left(r\right)=\frac{\rho_{A}^{\mathrm{sph}}\left(|r-R_{A}|\right)}{\sum_{B}\rho_{B}^{\mathrm{sph}}\left(|r-R_{B}|\right)}.$$

This implementation produces one Hirshfeld population and one Hirshfeld charge per atom.

### 2.3 Fragment Charges From Atomic Reference Densities

The second implementation extends the atomic scheme to user‐defined molecular fragments. In this case, each fragment is specified as a list of atoms, but its reference density is still constructed from spherical atomic WFX densities. For a fragment F, the reference density is

(6) $$\rho_{F}^{\mathrm{ref}}\left(r\right)=\sum_{A\in F}\rho_{A}^{\mathrm{sph}}\left(|r-R_{A}|\right).$$

The Hirshfeld weight of fragment F is then

(7) $$h_{F}\left(r\right)=\frac{\rho_{F}^{\mathrm{ref}}\left(r\right)}{\sum_{G}\rho_{G}^{\mathrm{ref}}\left(r\right)}.$$

The fragment population is obtained by integrating $h_{F}\left(r\right)\rho \left(r\right)$ over the molecular density of the full system. This approach allows one to report charge transfer between molecular fragments while retaining a purely promolecular, atom‐based construction of the reference density.

### 2.4 Fragment Charges From Molecular WFX Reference Densities

The third implementation uses molecular WFX files as reference densities for the fragments. In this case, the reference density of fragment F is reconstructed directly from the WFX file of the isolated fragment,

(8) $$\rho_{F}^{\mathrm{ref}}\left(r\right)=\rho_{F}^{\mathrm{WFX}}\left(r\right).$$

The fragment WFX files must be generated for the isolated fragments in the frozen geometry they adopt within the complex and in the same Cartesian frame as the WFX file of the full system, so that the fragment densities are spatially consistent with the cluster geometry. The Hirshfeld population is again obtained from

(9) $$N_{F}=\int \frac{\rho_{F}^{\mathrm{WFX}}\left(r\right)}{\sum_{G}\rho_{G}^{\mathrm{ref}}\left(r\right)}\rho \left(r\right)\;dr.$$

This implementation differs from the atom‐based fragment scheme because the reference density of a fragment includes the molecular electronic structure of the isolated fragment, rather than a simple sum of spherical atomic densities. It therefore provides a way to assess how the choice of reference density affects the magnitude and direction of the computed charge transfer.

### 2.5 Mixed Fragment/Atomic Representation

The fragment implementation also allows mixed reference partitions. Some parts of the system can be defined as molecular fragments, either through atom‐based reference densities or through molecular WFX reference densities, while the remaining atoms can be retained as independent atomic entities. In this mode, the denominator of the Hirshfeld weight contains all reference entities simultaneously,

(10) $$\sum_{Y}\rho_{Y}^{\mathrm{ref}}\left(r\right)=\sum_{\text{fragments}}\rho_{F}^{\mathrm{ref}}\left(r\right)+\sum_{\text{atoms}}\rho_{A}^{\mathrm{sph}}\left(r\right).$$

This option is useful when only selected regions of a system are to be treated as molecular fragments, while the rest of the system remains atom‐resolved. The implementation can also assign unlisted atoms automatically as independent atomic entities.

### 2.6 Treatment of Charged Fragments

For fragment WFX references, an optional formal charge can be supplied in the fragment‐definition file. This value is not used to alter the Hirshfeld formula, since the charge is always computed as

(11) $$q_{F}=Z_{F}^{\mathrm{ref}}-N_{F}.$$

Instead, the declared formal charge is used as metadata and as a consistency check against the charge inferred from the fragment WFX file, namely the difference between the nuclear charge and the electron occupation of the reference fragment. This feature allows charged fragments, such as hydronium or hydroxide, to be included in the same WFX‐based framework, although the systems reported in the present work are neutral non‐covalent complexes.

The DFT calculations were performed using B3LYP‐D3BJ [27, 28, 29, 30, 31], ωB97X‐D [32], and M06‐2X [33], all with the 6‐311++G** basis set [34, 35]. The complete DFT results obtained with the 6‐311++G** basis set are reported in Tables S1 and S2 of the Supporting Information. To assess the basis‐set dependence of the fragment charges, all DFT calculations for the water clusters and the S66 set were repeated with the smaller 6‐31G* basis set [36], while retaining the same geometries, fragment definitions, and numerical‐integration parameters. The complete results are reported in Tables S4 and S5 of the Supporting Information. The available CCSD calculations were performed with the same 6‐311++G** basis set [37], and the corresponding results are reported in Table S3 of the Supporting Information. WFX files were interpreted according to the AIMAll/WFX specification of Keith [38, 39]. The present implementation was developed in C for CPU execution. A GPU‐accelerated implementation within the GPUAM code base is currently under development, and release of the source code as open‐source software is planned.

## 3 Results and Discussion

**FIGURE 1:**
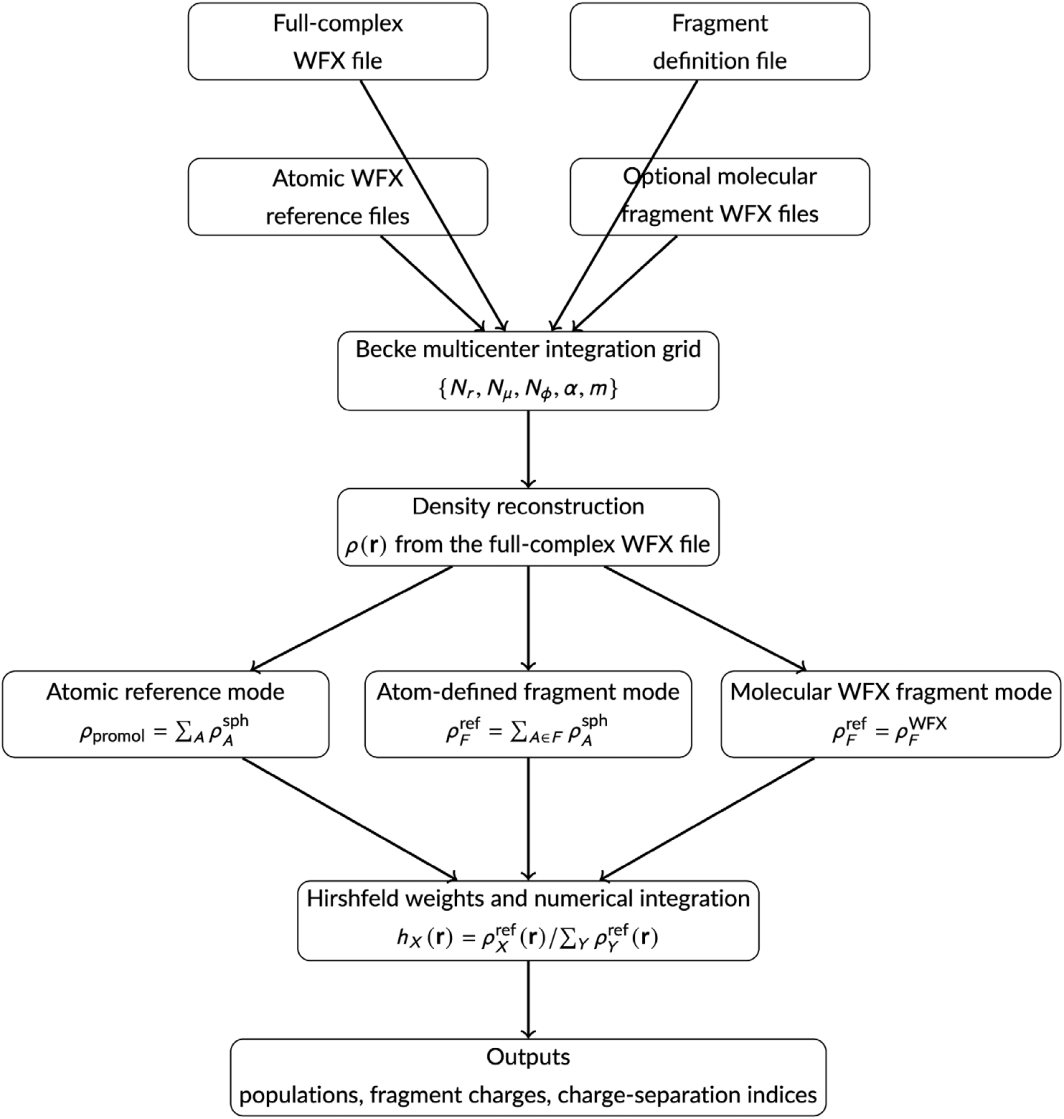
Schematic workflow of the WFX‐based Hirshfeld implementation. The electron density of the full complex is reconstructed from its WFX file and integrated on a Becke multicenter grid. The same integration framework can be used with atomic spherical WFX references, atom‐defined molecular fragments constructed from atomic references, or molecular WFX reference densities of isolated fragments. The code reports populations, charges, charge‐separation indices, normalization diagnostics, and optional consistency checks for charged fragments.

The computational workflow summarized in Figure 1 was used to compare atomic‐reference and molecular‐fragment‐reference Hirshfeld charges on the same numerical grid. The results are presented in two stages. We first analyze the water clusters as a chemically controlled test case, where all fragments correspond to water molecules and the main variables are the topology and symmetry of the hydrogen‐bond network. We then extend the analysis to a representative subset of the S66 benchmark set, which includes hydrogen‐bonded, dispersion‐dominated, π‐stacked, and mixed non‐covalent complexes. This order allows us to establish the methodological effect of replacing atomic references by molecular WFX fragment references in a homogeneous family of systems before examining its transferability to a chemically diverse benchmark.

### 3.1 Water Clusters

**FIGURE 2:**
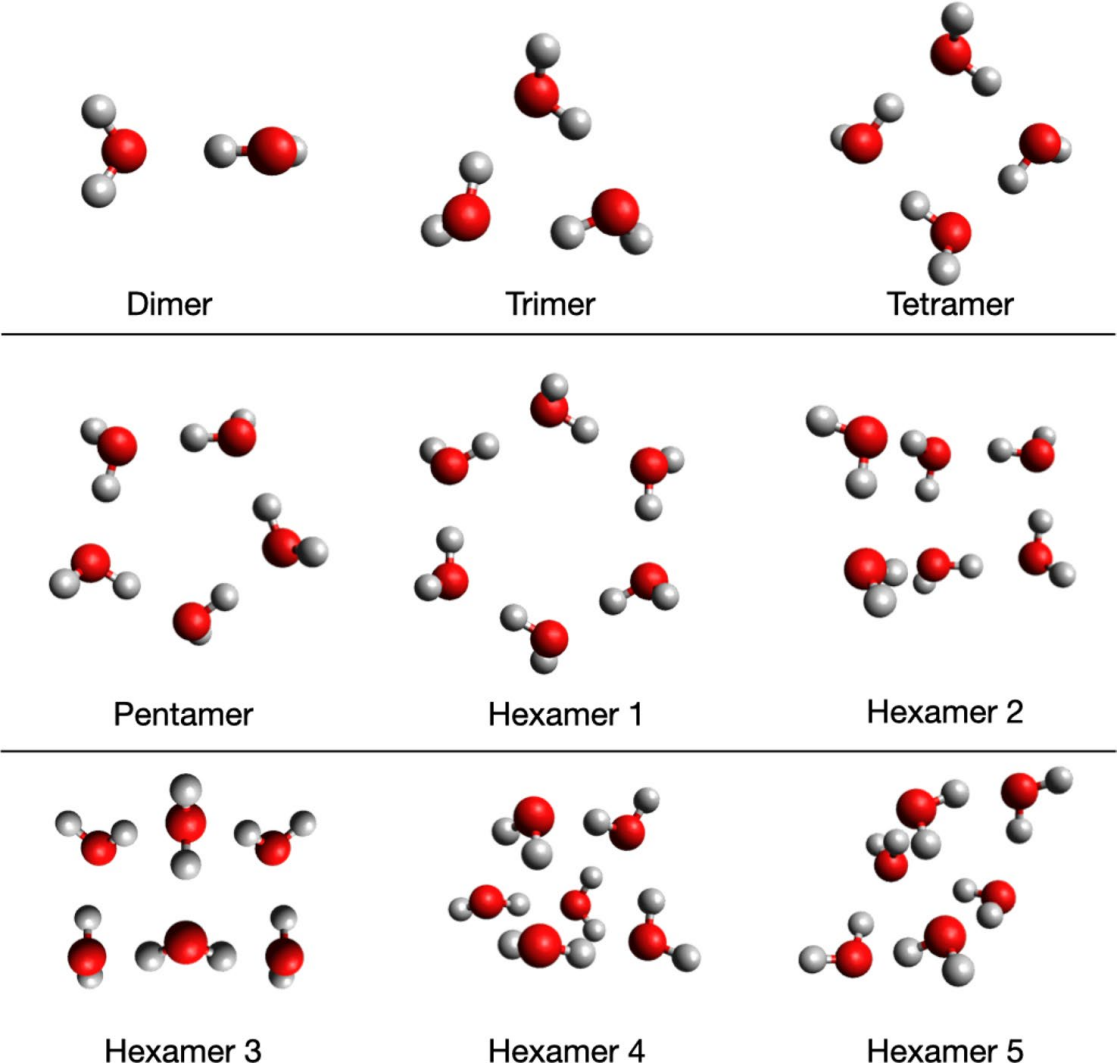
Structures of the water clusters considered in this work, generated from the geometries reported in Reference [19].

The water clusters provide a well‐defined test for the fragment‐based Hirshfeld scheme because all fragments have the same chemical identity. In contrast to chemically heterogeneous dimers, where the two fragments may have very different electronic structures, the fragments in these clusters are all water molecules. Therefore, the observed differences mainly reflect the hydrogen‐bond network, the structural arrangement of the cluster, and the choice of the Hirshfeld reference density. The structures of the water clusters are presented in Figure 2.

To avoid presenting the charge of every individual water molecule in the main text, Table 1 reports a total charge‐separation index for each cluster,

$$Q_{\text{atom}}=\sum_{i}|q_{i,\text{atom}}|,\;Q_{\mathrm{WFX}}=\sum_{i}|q_{i,\mathrm{WFX}}|.$$

**TABLE 1:** Total Hirshfeld charge‐separation index for nine water clusters [19].

Water cluster	Method	$Q_{\text{atom}}$	$Q_{\mathrm{WFX}}$	Reduction (%)
Dimer	B3LYP‐D3BJ	0.1894	0.0312	83.5
	ωB97X‐D	0.1886	0.0292	84.5
	M06‐2X	0.1838	0.0268	85.4
Trimer	B3LYP‐D3BJ	0.0092	0.0036	60.9
	ωB97X‐D	0.0092	0.0034	63.0
	M06‐2X	0.0088	0.0033	62.5
Tetramer	B3LYP‐D3BJ	0.0000	0.0000	—
	ωB97X‐D	0.0000	0.0000	—
	M06‐2X	0.0000	0.0000	—
Pentamer	B3LYP‐D3BJ	0.0090	0.0051	43.3
	ωB97X‐D	0.0088	0.0048	45.5
	M06‐2X	0.0089	0.0049	44.9
Hexamer 1	B3LYP‐D3BJ	0.0000	0.0000	—
	ωB97X‐D	0.0000	0.0000	—
	M06‐2X	0.0000	0.0000	—
Hexamer 2	B3LYP‐D3BJ	0.1430	0.0502	64.9
	ωB97X‐D	0.1432	0.0521	63.6
	M06‐2X	0.1386	0.0560	59.6
Hexamer 3	B3LYP‐D3BJ	0.1747	0.0383	78.1
	ωB97X‐D	0.1750	0.0395	77.4
	M06‐2X	0.1702	0.0422	75.2
Hexamer 4	B3LYP‐D3BJ	0.1190	0.0090	92.4
	ωB97X‐D	0.1194	0.0094	92.1
	M06‐2X	0.1158	0.0114	90.2
Hexamer 5	B3LYP‐D3BJ	0.1234	0.0382	69.0
	ωB97X‐D	0.1236	0.0381	69.2
	M06‐2X	0.1160	0.0418	64.0

*Note:* For each cluster, $Q_{\text{atom}}=\sum_{i}|q_{i,\text{atom}}|$ was obtained by summing the absolute values of the fragment charges computed from atomic Hirshfeld references, whereas $Q_{\mathrm{WFX}}=\sum_{i}|q_{i,\mathrm{WFX}}|$ was obtained using molecular WFX files for the isolated water fragments. The reduction percentage is defined as $100\left[1-Q_{\mathrm{WFX}}/Q_{\text{atom}}\right]$. Cases with $Q_{\text{atom}}=0$ correspond to symmetric arrangements for which the reduction percentage is not defined.

These quantities should not be interpreted as total charges of the cluster, which are zero for the neutral systems considered here. Instead, they measure the magnitude of the charge separation among molecular fragments. The complete set of individual fragment charges is provided in the Supporting Information.

For all water clusters and all density functionals, the fragment charges obtained by summing atomic Hirshfeld charges are identical, within the reported numerical precision, to those obtained by defining each water molecule as an atomic fragment while retaining atomic promolecular references. This equivalence was verified numerically for all systems, and the complete individual fragment charges are reported in the Supporting Information. This confirms that the use of atom‐defined fragments does not modify the net fragment charge when the underlying promolecular density is still constructed from isolated atoms. The relevant methodological change occurs only when the isolated molecular WFX files of the water fragments are used to construct the promolecular reference.

The use of molecular WFX fragment references leads to a substantial reduction in the charge‐separation index. This effect is particularly evident for the water dimer, where the atomic‐reference scheme gives $Q_{\text{atom}}\approx 0.18–0.19\;\;e$, whereas the WFX‐fragment reference gives $Q_{\mathrm{WFX}}\approx 0.03\;\;e$. Equivalently, the charge on each water molecule decreases from approximately 0.09e with atomic references to about 0.01–0.02e with molecular WFX references. Thus, the molecular‐fragment reference removes a large fraction of the apparent charge separation obtained from isolated atomic references.

This behavior follows from the different physical content of the reference densities. When atomic references are used, the Hirshfeld partition measures the redistribution of the total density relative to a promolecule constructed from isolated spherical atoms. In water clusters, this reference does not contain the electronic structure of the isolated water molecule. As a consequence, part of the resulting fragment charge reflects the reconstruction of molecular water from atomic densities, in addition to the redistribution associated with intermolecular hydrogen bonding. In contrast, when WFX files of the isolated water molecules are used as references, the intramolecular electronic structure of each monomer is already included in the promolecular density. The remaining redistribution is therefore more directly associated with intermolecular effects within the hydrogen‐bond network.

The magnitude of the charge‐separation index also depends strongly on the topology and symmetry of the water cluster. The tetramer and one of the hexamer structures give essentially zero net charge on each molecular fragment for all three functionals, consistent with highly symmetric hydrogen‐bond arrangements in which donor and acceptor roles are balanced. It should be emphasized that a zero net fragment charge does not imply the absence of local density redistribution, polarization, or directional intermolecular charge‐transfer contributions. The present analysis integrates the density assigned to each complete molecular fragment and therefore reports only the net charge gained or lost by that fragment. In highly symmetric systems, symmetry‐related local contributions may cancel exactly in the integrated fragment charge. Consequently, a description based on a single integrated charge per molecular fragment does not resolve pairwise or symmetry‐cancelled charge‐transfer processes. Such effects would require a finer fragment subdivision or a complementary spatial or pairwise analysis. The other hexamer isomers display sizable charge separations when atomic references are used, indicating that the local hydrogen‐bond environment can generate distinguishable donor‐like and acceptor‐like water molecules within the cluster. However, these charge separations are again strongly reduced when molecular WFX references are used.

The reduction produced by the WFX fragment references is not uniform across the water clusters. Instead, it depends on the specific structural motif. For example, the reduction is approximately 84%–85% for the water dimer, 43%–46% for the pentamer, and above 90% for water hexamer 4. This shows that the WFX‐fragment scheme does not simply scale the atomic‐reference charges by a constant factor. Rather, changing the reference from isolated atoms to isolated molecular fragments modifies the local balance of charge redistribution in a structure‐dependent way. In some individual water molecules within the hexamers, even the sign of the fragment charge can change upon replacing atomic references by WFX fragment references. This emphasizes that the molecular‐fragment reference defines a different physical partitioning problem, not merely a numerical correction to the atomic‐reference result.

The three density functionals lead to the same qualitative conclusions. B3LYP‐D3BJ and ωB97X‐D generally give very similar charge‐separation indices, while M06‐2X shows a slightly different response in some clusters. In particular, M06‐2X often gives slightly smaller atomic‐reference charge separations, but in some hexamers it yields somewhat larger WFX‐fragment values. Nevertheless, the dominant trend is independent of the functional: molecular WFX references produce significantly smaller fragment charge separations than atomic references.

Taken together, the water‐cluster results establish the main methodological conclusion in a homogeneous and chemically controlled family of systems. Fragment charges based on atomic references and atom‐defined fragments are internally consistent but remain tied to an atomic promolecular picture. In contrast, WFX‐based molecular fragment references provide a chemically more appropriate reference state for discussing charge redistribution between preformed molecular units. For hydrogen‐bonded water networks, this distinction is essential because it separates the electronic structure of the isolated water monomers from the additional redistribution induced by cluster formation.

### 3.2 Representative Complexes From the S66 Set

**FIGURE 3:**
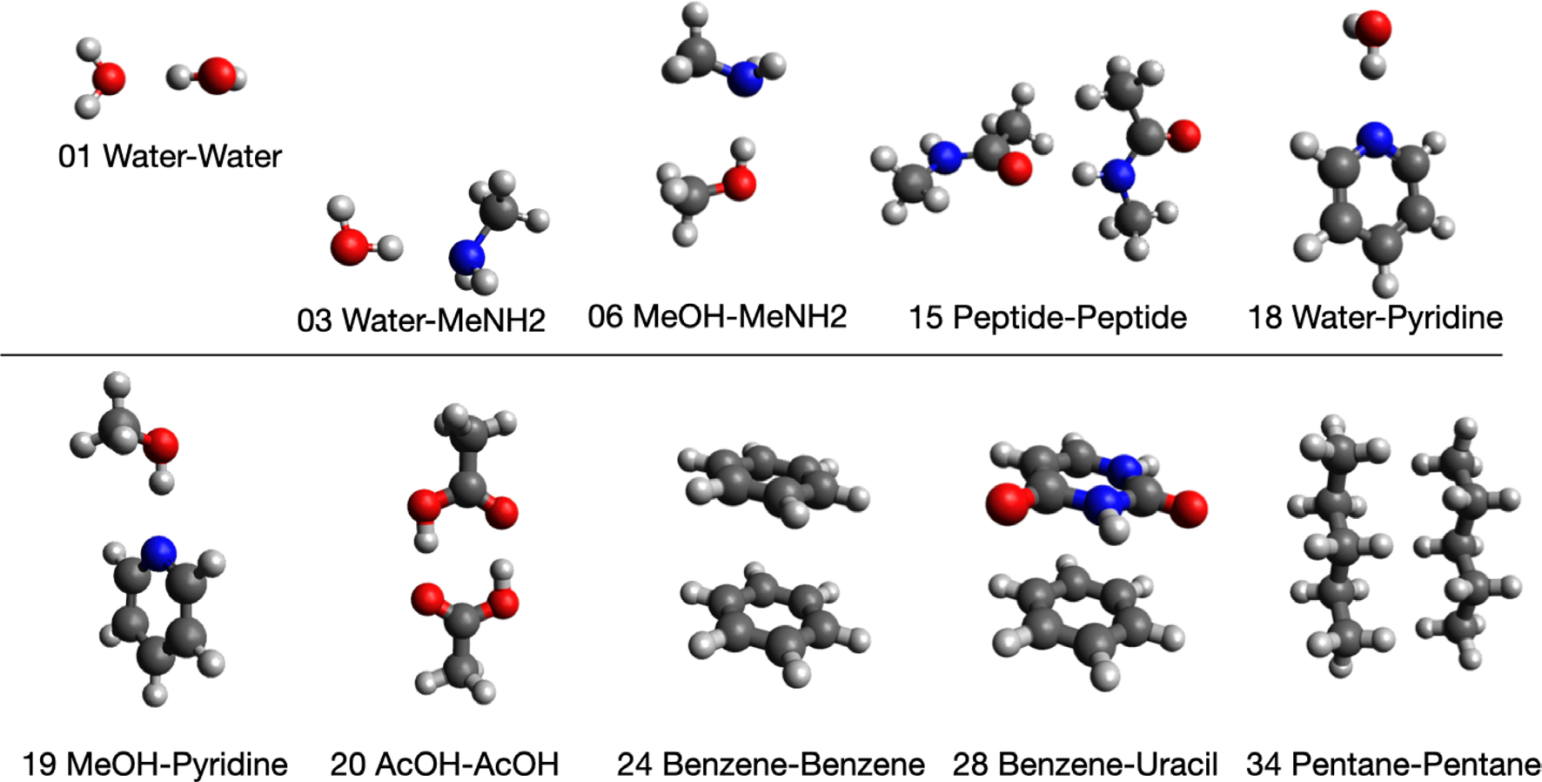
Structures of the selected S66 complexes considered in Table 2, generated from the geometries reported in Reference [20].

After establishing the effect of the molecular‐fragment reference in water clusters, we now examine whether the same behavior is observed in a chemically diverse set of non‐covalent complexes. For this purpose, Table 2 reports a representative subset of the S66 benchmark set at the DFT level. Their structures are presented in Figure 3. The selected systems include hydrogen‐bonded complexes, biomolecular hydrogen‐bond models, symmetric dimers, π‐stacked complexes, and dispersion‐dominated systems. The complete S66 table is provided in the Supporting Information. CCSD results are reported separately in Table 3 for the systems for which this level of theory was computationally feasible.

**TABLE 2:** Representative Hirshfeld fragment charges for selected complexes of the S66 set.

System	Method	Transferred charge		
		$|q_{\text{atom}}|$	$|q_{\mathrm{WFX}}|$	Reduction (%)
01_Water‐Water	B3LYP‐D3BJ	0.0934	0.0144	84.6
	ωB97X‐D	0.0931	0.0135	85.5
	M06‐2X	0.0909	0.0125	86.2
03_Water‐MeNH2	B3LYP‐D3BJ	0.1295	0.0238	81.6
	ωB97X‐D	0.1284	0.0216	83.2
	M06‐2X	0.1265	0.0204	83.9
06_MeOH‐MeNH2	B3LYP‐D3BJ	0.1359	0.0268	80.3
	ωB97X‐D	0.1350	0.0247	81.7
	M06‐2X	0.1333	0.0239	82.1
15_Peptide‐Peptide	B3LYP‐D3BJ	0.0987	0.0255	74.2
	ωB97X‐D	0.0984	0.0243	75.3
	M06‐2X	0.0959	0.0228	76.2
18_Water‐Pyridine	B3LYP‐D3BJ	0.1234	0.0285	76.9
	ωB97X‐D	0.1229	0.0258	79.0
	M06‐2X	0.1209	0.0246	79.7
19_MeOH‐Pyridine	B3LYP‐D3BJ	0.1344	0.0314	76.6
	ωB97X‐D	0.1338	0.0289	78.4
	M06‐2X	0.1320	0.0279	78.9
20_AcOH‐AcOH	B3LYP‐D3BJ	0.0000	0.0000	—
	ωB97X‐D	0.0000	0.0000	—
	M06‐2X	0.0000	0.0000	—
24_Benzene‐Benzene_pi‐pi	B3LYP‐D3BJ	0.0000	0.0000	—
	ωB97X‐D	0.0000	0.0000	—
	M06‐2X	0.0000	0.0000	—
28_Benzene‐Uracil_pi‐pi	B3LYP‐D3BJ	0.0068	0.0025	63.2
	ωB97X‐D	0.0064	0.0030	53.1
	M06‐2X	0.0080	0.0016	80.0
34_Pentane‐Pentane	B3LYP‐D3BJ	0.0000	0.0000	—
	ωB97X‐D	0.0000	0.0000	—
	M06‐2X	0.0000	0.0000	—

*Note:* The atomic‐reference charge, $q_{\text{atom}}$, corresponds to the sum of atomic Hirshfeld charges over the selected fragment. Since $q_{\mathrm{sum}}$ and $q_{\text{atomic}}$ are identical within numerical precision, only $q_{\text{atom}}$ is reported. The WFX‐fragment charge, $q_{\mathrm{WFX}}$, was obtained using molecular WFX files for the isolated fragments. The reduction percentage is defined as $100\left[1-|q_{\mathrm{WFX}}|/|q_{\text{atom}}|\right]$.

**TABLE 3:** Condensed CCSD Hirshfeld charge‐transfer analysis for the systems for which correlated WFX calculations were available.

System	Set	Quantity	Atomic ref.	WFX ref.	Reduction (%)
Dimer 1	water cluster	Q	0.1788	0.0244	86.4
Trimer 1	water cluster	Q	0.0091	0.0032	64.8
Tetramer 1	water cluster	Q	0.0000	0.0000	—
Pentamer 1	water cluster	Q	0.0090	0.0046	48.9
Hexamer 1	water cluster	Q	0.0000	0.0000	—
Hexamer 2	water cluster	Q	0.1296	0.0535	58.7
Hexamer 3	water cluster	Q	0.1638	0.0388	76.3
Hexamer 4	water cluster	Q	0.1123	0.0118	89.5
Hexamer 5	water cluster	Q	0.1149	0.0391	66.0
01_Water‐Water	S66	$|q_{A}|$	0.0883	0.0114	87.1
03_Water‐MeNH2	S66	$|q_{A}|$	0.1246	0.0181	85.5
06_MeOH‐MeNH2	S66	$|q_{A}|$	0.1310	0.0213	83.7
18_Water‐Pyridine	S66	$|q_{A}|$	0.1195	0.0222	81.4
19_MeOH‐Pyridine	S66	$|q_{A}|$	0.1299	0.0251	80.7

*Note:* For nine water clusters, $Q_{\text{atom}}=\sum_{i}|q_{i,\text{atom}}|$ and $Q_{\mathrm{WFX}}=\sum_{i}|q_{i,\mathrm{WFX}}|$ measure the total charge separation among molecular fragments. For the S66 dimers, the reported quantity is $|q_{A}|$, the absolute charge of the first molecular fragment. The reduction percentage is defined as $100\left[1-X_{\mathrm{WFX}}/X_{\text{atom}}\right]$, where X denotes either Q for water clusters or $|q_{A}|$ for S66 dimers.

The S66 results confirm that the trends identified for the water clusters are not restricted to hydrogen‐bonded networks of identical monomers. When atomic promolecular densities are used as references, the net charge obtained by summing the atomic Hirshfeld charges over a given molecular fragment, $q_{\mathrm{sum}}$, is essentially identical to the charge obtained by explicitly defining that fragment as a collection of atoms, $q_{\text{atomic}}$. The differences between both quantities are zero within the numerical precision of the calculations; the corresponding complete data are provided in the Supporting Information. This result is expected because both procedures rely on the same set of isolated atomic reference densities. Nevertheless, it provides an important internal consistency check: the fragment charge obtained from atomic references is invariant with respect to whether the population is accumulated atom by atom or reported directly for a predefined fragment.

A qualitatively different behavior is observed when the reference density of each fragment is constructed from its molecular WFX file. In this case, the resulting fragment charges, $q_{\mathrm{WFX}}$, are systematically smaller in magnitude than those obtained from atomic references. This reduction is observed across the representative S66 subset and is particularly significant for systems in which the atomic‐reference scheme predicts relatively large charge transfers. Thus, the use of molecular fragment WFX references leads to a more conservative estimate of intermolecular charge transfer.

This behavior can again be understood from the different physical content of the reference densities. In the atomic‐reference scheme, each monomer is represented as a superposition of isolated spherical atoms. Therefore, the Hirshfeld partition must account not only for changes associated with the interaction between the two monomers, but also for the reconstruction of the intramolecular electronic structure of each monomer from atomic densities. In contrast, when molecular WFX files are used for the isolated fragments, each monomer enters the promolecular reference as a preformed electronic entity. The intramolecular density redistribution is already incorporated in the reference state. Consequently, the remaining charge redistribution measured by the Hirshfeld partition is more directly associated with the interaction between the molecular fragments.

From this perspective, the larger charge transfers obtained with atomic references should not necessarily be interpreted as purely intermolecular charge transfer. They also contain contributions associated with the use of independent atomic densities to represent molecular fragments. The WFX‐fragment scheme removes much of this contribution by using a reference density that is chemically closer to the isolated monomers. Therefore, the difference between $q_{\text{atom}}$ and $q_{\mathrm{WFX}}$ is not merely a numerical effect, but reflects a change in the physical definition of the reference state used in the Hirshfeld analysis.

The chemical classification of the S66 set is reflected in the magnitude of the fragment charges. Hydrogen‐bonded complexes display the largest charge transfers when atomic references are used. Systems such as water–methylamine, methanol–methylamine, water–pyridine, and methanol–pyridine show relatively large values of $q_{\text{atom}}$, consistent with the presence of clear donor–acceptor interactions. When molecular WFX fragment references are used, these charge transfers are strongly reduced but remain appreciable, indicating that some degree of intermolecular density redistribution persists even after the isolated molecular fragments are used as the reference state.

In contrast, dispersion‐dominated complexes and symmetric π‐stacked systems exhibit zero or nearly zero charge transfer, particularly with the WFX‐fragment reference. This behavior is consistent with the expectation that dispersion‐dominated interactions should involve little net charge transfer between monomers. The heteromolecular benzene–uracil π–π complex gives small but nonzero fragment charges, and the reduction depends more noticeably on the functional. This is also expected because, for very small values of $q_{\text{atom}}$, ratios such as $|q_{\mathrm{WFX}}|/|q_{\text{atom}}|$ become more sensitive to small absolute changes.

An additional feature of the results is the functional dependence of the charge redistribution. The values obtained with B3LYP‐D3BJ and ωB97X‐D are generally very similar, especially for the charges based on atomic references. M06‐2X, however, displays a somewhat different response. In several hydrogen‐bonded complexes, M06‐2X gives slightly smaller atomic‐reference charge transfers than the other two functionals. At the same time, the reduction produced by replacing atomic references with molecular WFX fragment references is often particularly pronounced for M06‐2X. This indicates that the sensitivity of the Hirshfeld charges to the definition of the reference density is not completely independent of the exchange–correlation functional.

It is important to note that reduction percentages or ratios such as $|q_{\mathrm{WFX}}|/|q_{\text{atom}}|$ must be interpreted with caution for systems in which $q_{\text{atom}}$ is very small or exactly zero. In such cases, even a small absolute change in the charge can produce a large or apparently anomalous ratio, and the percentage is not defined when $q_{\text{atom}}=0$. Therefore, for weakly interacting, symmetric, or dispersion‐dominated systems, the absolute magnitudes of the charges provide a more reliable criterion than relative ratios.

Overall, the S66 results demonstrate the transferability of the conclusion obtained from the water clusters. Atomic references and atom‐defined fragments lead to equivalent fragment charges, confirming the internal consistency of the implementation. However, replacing the atomic promolecule by a promolecule constructed from molecular WFX fragments leads to systematically smaller charge transfers. This indicates that molecular‐fragment references provide a different and chemically more appropriate reference state for the analysis of intermolecular charge redistribution. Rather than measuring the redistribution relative to isolated atoms, the WFX‐fragment scheme measures the redistribution relative to isolated molecular fragments. This distinction is essential when Hirshfeld charges are used to discuss charge transfer in non‐covalent complexes.

The 6‐31G* calculations preserve the main qualitative conclusion obtained with 6‐311++G**. Molecular WFX fragment references continue to yield substantially smaller charges than atomic references for hydrogen‐bonded and donor–acceptor complexes, although the absolute WFX‐fragment charges exhibit a more noticeable basis‐set dependence. For example, at the B3LYP‐D3BJ level, the reduction changes from 83.5% to 67.7% for the water dimer, from 81.6% to 69.1% for water–methylamine, and from 80.3% to 68.4% for methanol–methylamine. Symmetry‐enforced zero net fragment charges are preserved. These results indicate that the reference‐state effect is robust, while the precise magnitude of small fragment charges should be interpreted with regard to the basis set.

### 3.3 Correlated‐Wave‐Function Validation With CCSD

Because CCSD calculations for the largest complexes are computationally demanding, this level of theory was not used as a production method for the complete representative set. Instead, CCSD was employed as a correlated wave‐function validation for all systems for which the full‐complex and isolated‐fragment WFX files could be obtained. The results are condensed in Table 3. For the water clusters, the table reports the total charge‐separation index, $Q=\sum_{i}|q_{i}|$, because this quantity summarizes the redistribution among all water molecules without listing every individual fragment. For the S66 dimers, where the two fragment charges have equal magnitude and opposite sign, the table reports the absolute value of the charge of fragment A, $|q_{A}|$.

The CCSD results collected in Table 3 provide an important correlated wave‐function test of the conclusions obtained at the DFT level. Although the CCSD set is necessarily smaller than the complete DFT data set, it includes two complementary groups of systems: a systematic series of nine water clusters and several strongly interacting hydrogen‐bonded or donor–acceptor complexes from S66. In both groups, replacing the atomic promolecular reference by molecular WFX fragment references produces a marked decrease in the apparent charge transfer.

For the water clusters, the CCSD charge‐separation index follows the same structure‐dependent pattern observed with the density functionals. Symmetric arrangements such as water tetramer 1 and water hexamer 1 show essentially zero charge separation with both references, indicating that the method preserves the expected cancellation imposed by the balanced hydrogen‐bond topology. In contrast, the less symmetric hexamer isomers exhibit sizable atomic‐reference charge separation, with $Q_{\text{atom}}$ values between 0.1123 and 0.1638 e for hexamers 2–5. When molecular WFX references are used, these values decrease to 0.0118–0.0535 e, corresponding to reductions of approximately 59%–90%. The water dimer shows an even clearer effect: Q decreases from 0.1788 e with atomic references to 0.0244 e with WFX fragment references. Therefore, the correlated calculation confirms that much of the charge separation obtained from isolated atomic references is not retained when each water molecule is represented as a preformed molecular fragment.

The CCSD S66 results reinforce the same interpretation for chemically heterogeneous complexes. For water–methylamine, methanol–methylamine, water–pyridine, and methanol–pyridine, the atomic‐reference charges are in the range 0.1195–0.1310 e, whereas the WFX‐fragment charges are only 0.0181–0.0251 e. The corresponding reductions, 80.7%–85.5%, are very close to those obtained for the water dimer. This consistency is significant because these systems involve different donor and acceptor fragments, rather than identical water monomers. Thus, the reduction is not an artifact of symmetry or of the water‐cluster test set; it is a consequence of changing the reference state in the Hirshfeld partition.

Taken together, the CCSD data show that the main methodological conclusion is not tied to a particular density functional. Atomic references systematically assign larger fragment charges because they compare the molecular density of the complex with a promolecule assembled from isolated atoms. Molecular WFX references instead compare the complex density with a promolecular density built from isolated, already bonded fragments. The latter reference removes the intramolecular contribution associated with forming each monomer from atoms and leaves a smaller charge redistribution that is more directly associated with the interaction between fragments. The CCSD calculations therefore support the use of WFX molecular fragments as a chemically meaningful reference for Hirshfeld charge‐transfer analysis in non‐covalent systems.

## 4 Conclusions

A molecular‐fragment formulation of Hirshfeld population analysis based on WFX reference densities has been introduced and tested for non‐covalent complexes. In this approach, the electron density of the full system is partitioned using reference densities obtained either from isolated spherical atoms or from isolated molecular fragments represented by their own WFX wave functions. This makes it possible to evaluate how the definition of the promolecular reference affects the magnitude and interpretation of fragment charges.

The results show that defining molecular fragments as collections of atoms does not modify the net fragment charge when the underlying promolecular density is still constructed from isolated atomic references. In that case, the charge obtained by summing atomic Hirshfeld charges over a fragment is equivalent, within numerical precision, to the charge obtained by explicitly defining the same group of atoms as a fragment. This confirms the internal consistency of the implementation and establishes that the decisive methodological change is not the grouping of atoms itself, but the replacement of atomic reference densities by molecular WFX fragment densities.

For the water clusters considered in this article, molecular WFX fragment references produce a strong reduction of the total charge‐separation index relative to atomic references. Symmetric hydrogen‐bond arrangements, such as the water tetramer and one of the hexamer structures, give essentially zero charge separation with both reference schemes, as expected from their balanced topologies. Less symmetric clusters exhibit sizable charge separation when atomic references are used, but this separation is markedly reduced when each water molecule is described as a preformed molecular WFX fragment. The reduction is structure dependent rather than a simple uniform scaling, showing that the molecular‐fragment reference changes the physical partitioning problem.

The representative S66 complexes lead to the same conclusion in a more chemically diverse context. Hydrogen‐bonded and donor–acceptor complexes show the largest atomic‐reference fragment charges, whereas symmetric dimers, dispersion‐dominated systems, and symmetric π‐stacked complexes display zero or nearly zero net integrated fragment charge. For symmetric systems, this result may reflect cancellation of local contributions and should not be interpreted as evidence for the absence of local density redistribution. Upon replacing atomic references with molecular WFX fragment references, the charge‐transfer magnitudes are systematically reduced. This reduction indicates that part of the charge separation obtained with atomic references originates from the use of isolated atoms to represent molecular fragments, rather than from intermolecular charge transfer alone. Calculations with the smaller 6‐31G* basis set show a noticeable quantitative dependence of the WFX‐fragment charges, but preserve their systematic reduction relative to atomic‐reference charges.

The CCSD calculations provide an important correlated‐wave‐function validation of the trends observed at the density‐functional level. Although the CCSD data set is smaller because of computational cost, it includes both water clusters and representative hydrogen‐bonded or donor–acceptor complexes from S66. In all cases where nonzero charge separation is found, the molecular WFX fragment reference gives substantially smaller charge transfer than the atomic reference. Therefore, the main conclusion is not an artifact of a particular exchange–correlation functional, but follows from the reference state used in the Hirshfeld partition.

In summary, the WFX molecular‐fragment formulation provides a more chemically appropriate reference for discussing charge transfer between preformed molecular units. Atomic Hirshfeld references remain useful for atomic population analysis, but fragment charge‐transfer analysis in non‐covalent complexes benefits from a reference that already contains the intramolecular electronic structure of each isolated monomer. The proposed scheme therefore separates, at least at the level of the density‐based reference, the formation of the molecular fragments from the additional charge redistribution induced by intermolecular interaction.

Extensions of the present WFX‐fragment reference strategy to iterative, variational, and scaled Hirshfeld schemes [13, 15, 40] are natural directions for future work, because self‐consistent or variational updates of the reference densities may modify the balance between intramolecular and intermolecular charge redistribution.

## Funding

The authors have nothing to report.

## Conflicts of Interest

The authors declare no conflicts of interest.

## Supporting information


**Table S1:** Comparison of Hirshfeld charges obtained from the sum of atomic charges, fragments defined from atomic densities, and fragments defined through WFX files for water clusters. All calculations were obtained with the 6‐311++G** basis set.
**Table S2:** Comparison of Hirshfeld charges obtained from the sum of atomic charges, fragments defined from atomic densities, and fragments defined through WFX files. All calculations were obtained with the 6‐311++G** basis set.
**Table S3:** Hirshfeld charges obtained using fragments defined from atomic densities and fragments defined through WFX files from CCSD/6‐311++G** method.
**Table S4:** Comparison of Hirshfeld charges obtained from the sum of atomic charges, fragments defined from atomic densities, and fragments defined through WFX files for water clusters. All calculations were obtained with the 6‐31G* basis set.
**Table S5:** Comparison of Hirshfeld charges obtained from the sum of atomic charges, fragments defined from atomic densities, and fragments defined through WFX files. All calculations were obtained with the 6‐31G* basis set.

## Data Availability

The data that support the findings of this study are available in the Supporting Information of this article.
