# Supplementary material for: WFX Molecular Fragment References for Hirshfeld Charge‐Transfer Analysis in Non‐Covalent Complexes
Source: J Comput Chem. 2026 Jul 29;47(20):e70476. doi: 10.1002/jcc.70476 (PMC13417543; doi:10.1002/jcc.70476)
Supplement: Supplementary file 1 — Table S1: Comparison of Hirshfeld charges obtained from the sum of atomic charges, fragments defined from atomic densities, and fragments defined through WFX files for water clusters. All calculations were obtained with the 6‐311++G** basis set. Table S2: Comparison of Hirshfeld charges obtained from the sum of atomic charges, fragments defined from atomic densities, and fragments defined through WFX files. All calculations were obtained with the 6‐311++G** basis set. Table S3: Hirshfeld charges obtained using fragments defined from atomic densities and fragments defined through WFX files from CCSD/6‐311++G** method. Table S4: Comparison of Hirshfeld charges obtained from the sum of atomic charges, fragments defined from atomic densities, and fragments defined through WFX files for water clusters. All calculations were obtained with the 6‐31G* basis set. Table S5: Comparison of Hirshfeld charges obtained from the sum of atomic charges, fragments defined from atomic densities, and fragments defined through WFX files. All calculations were obtained with the 6‐31G* basis set. [file JCC-47-0-s001.pdf]

WFX Molecular Fragment References for Hirshfeld Charge-Transfer Analysis in Non-Covalent Complexes

Jorge Garza and Rubicelia Vargas

Departamento de Química, División de Ciencias Básicas e Ingeniería,

Universidad Autónoma Metropolitana Iztapalapa,

San Rafael Atlixco 186, Col. Vicentina, C.P. 09340, Iztapalapa, CDMX, México.

Corresponding author: jgo@xanum.uam.mx

Contents

1 Water clusters/6-311++G** 1

2 S66 set/6-311++G** 5

3 CCSD results/6-311++G** 15

4 Water clusters/6-31G* 16

5 S66 set/6-31G* 19

1 Water clusters/6-311++G**

Table S1: Comparison of Hirshfeld charges obtained from the sum of atomic charges, fragments defined from atomic densities, and fragments defined through WFX files for water clusters. All calculations were obtained with the 6-311++G** basis set.

System	Functional	Fragment	q_{sum}	q_{atomic}	q_{WFX}	Δ_{atomic}	Δ_{WFX}
01_water_dimer_1	B3LYP_D3BJ	cluster_1	-0.0947	-0.0947	-0.0156	-0.0000	0.0791
01_water_dimer_1	B3LYP_D3BJ	cluster_2	0.0947	0.0947	0.0156	0.0000	-0.0791

Continued on the next page

Table S1: Comparison of Hirshfeld charges obtained from the sum of atomic charges, fragments defined from atomic densities, and fragments defined through WFX files for water clusters. All calculations were obtained with the 6-311++G** basis set (continued).

System	Functional	Fragment	q_{sum}	q_{atomic}	q_{WFX}	Δ_{atomic}	Δ_{WFX}
01_water_dimer_1	M06_2X	cluster_1	-0.0919	-0.0919	-0.0134	-0.0000	0.0785
01_water_dimer_1	M06_2X	cluster_2	0.0919	0.0919	0.0134	0.0000	-0.0785
01_water_dimer_1	wB97XD	cluster_1	-0.0943	-0.0943	-0.0146	-0.0000	0.0797
01_water_dimer_1	wB97XD	cluster_2	0.0943	0.0943	0.0146	-0.0000	-0.0797
02_water_trimer_1	B3LYP_D3BJ	cluster_1	-0.0046	-0.0046	-0.0018	0.0000	0.0029
02_water_trimer_1	B3LYP_D3BJ	cluster_2	0.0010	0.0010	0.0007	0.0000	-0.0004
02_water_trimer_1	B3LYP_D3BJ	cluster_3	0.0036	0.0036	0.0011	0.0000	-0.0025
02_water_trimer_1	M06_2X	cluster_1	-0.0044	-0.0044	-0.0016	-0.0000	0.0028
02_water_trimer_1	M06_2X	cluster_2	0.0008	0.0008	0.0006	0.0000	-0.0002
02_water_trimer_1	M06_2X	cluster_3	0.0036	0.0036	0.0011	0.0000	-0.0025
02_water_trimer_1	wB97XD	cluster_1	-0.0046	-0.0046	-0.0017	-0.0000	0.0029
02_water_trimer_1	wB97XD	cluster_2	0.0009	0.0009	0.0006	0.0000	-0.0003
02_water_trimer_1	wB97XD	cluster_3	0.0037	0.0037	0.0011	-0.0000	-0.0026
03_water_tetramer_1	B3LYP_D3BJ	cluster_1	-0.0000	-0.0000	-0.0000	-0.0000	-0.0000
03_water_tetramer_1	B3LYP_D3BJ	cluster_2	-0.0000	-0.0000	-0.0000	-0.0000	-0.0000
03_water_tetramer_1	B3LYP_D3BJ	cluster_3	-0.0000	-0.0000	-0.0000	-0.0000	0.0000
03_water_tetramer_1	B3LYP_D3BJ	cluster_4	-0.0000	-0.0000	-0.0000	-0.0000	-0.0000
03_water_tetramer_1	M06_2X	cluster_1	-0.0000	-0.0000	-0.0000	0.0000	0.0000
03_water_tetramer_1	M06_2X	cluster_2	-0.0000	-0.0000	-0.0000	-0.0000	0.0000
03_water_tetramer_1	M06_2X	cluster_3	-0.0000	-0.0000	-0.0000	0.0000	0.0000
03_water_tetramer_1	M06_2X	cluster_4	-0.0000	-0.0000	-0.0000	-0.0000	0.0000
03_water_tetramer_1	wB97XD	cluster_1	-0.0000	-0.0000	-0.0000	0.0000	0.0000
03_water_tetramer_1	wB97XD	cluster_2	-0.0000	-0.0000	-0.0000	-0.0000	-0.0000
03_water_tetramer_1	wB97XD	cluster_3	-0.0000	-0.0000	-0.0000	0.0000	0.0000
03_water_tetramer_1	wB97XD	cluster_4	-0.0000	-0.0000	-0.0000	-0.0000	0.0000
04_water_pentamer_1	B3LYP_D3BJ	cluster_1	-0.0003	-0.0003	-0.0001	0.0000	0.0002
04_water_pentamer_1	B3LYP_D3BJ	cluster_2	0.0011	0.0011	0.0005	-0.0000	-0.0006
04_water_pentamer_1	B3LYP_D3BJ	cluster_3	0.0034	0.0034	0.0018	-0.0000	-0.0016
04_water_pentamer_1	B3LYP_D3BJ	cluster_4	-0.0042	-0.0042	-0.0024	0.0000	0.0018
04_water_pentamer_1	B3LYP_D3BJ	cluster_5	0.0000	0.0000	0.0003	0.0000	0.0002
04_water_pentamer_1	M06_2X	cluster_1	-0.0003	-0.0003	-0.0001	-0.0000	0.0001
04_water_pentamer_1	M06_2X	cluster_2	0.0010	0.0010	0.0004	0.0000	-0.0006
04_water_pentamer_1	M06_2X	cluster_3	0.0034	0.0034	0.0018	-0.0000	-0.0016
04_water_pentamer_1	M06_2X	cluster_4	-0.0041	-0.0041	-0.0024	-0.0000	0.0018
04_water_pentamer_1	M06_2X	cluster_5	-0.0001	-0.0001	0.0002	-0.0000	0.0003
04_water_pentamer_1	wB97XD	cluster_1	-0.0003	-0.0003	-0.0001	-0.0000	0.0002
04_water_pentamer_1	wB97XD	cluster_2	0.0010	0.0010	0.0004	0.0000	-0.0006
04_water_pentamer_1	wB97XD	cluster_3	0.0034	0.0034	0.0018	-0.0000	-0.0017
04_water_pentamer_1	wB97XD	cluster_4	-0.0041	-0.0041	-0.0023	0.0000	0.0018
04_water_pentamer_1	wB97XD	cluster_5	-0.0000	-0.0000	0.0002	-0.0000	0.0002
05_water_hexamer_1	B3LYP_D3BJ	cluster_1	-0.0000	-0.0000	-0.0000	0.0000	0.0000
05_water_hexamer_1	B3LYP_D3BJ	cluster_2	-0.0000	-0.0000	-0.0000	0.0000	-0.0000
05_water_hexamer_1	B3LYP_D3BJ	cluster_3	-0.0000	-0.0000	-0.0000	-0.0000	0.0000
05_water_hexamer_1	B3LYP_D3BJ	cluster_4	-0.0000	-0.0000	-0.0000	0.0000	0.0000

Continued on the next page

Table S1: Comparison of Hirshfeld charges obtained from the sum of atomic charges, fragments defined from atomic densities, and fragments defined through WFX files for water clusters. All calculations were obtained with the 6-311++G** basis set (continued).

System	Functional	Fragment	q_{sum}	q_{atomic}	q_{WFX}	Δ_{atomic}	Δ_{WFX}
05_water_hexamer_1	B3LYP_D3BJ	cluster_5	-0.0000	-0.0000	-0.0000	-0.0000	-0.0000
05_water_hexamer_1	B3LYP_D3BJ	cluster_6	-0.0000	-0.0000	-0.0000	0.0000	0.0000
05_water_hexamer_1	M06_2X	cluster_1	0.0000	0.0000	0.0000	-0.0000	-0.0000
05_water_hexamer_1	M06_2X	cluster_2	-0.0000	-0.0000	-0.0000	-0.0000	-0.0000
05_water_hexamer_1	M06_2X	cluster_3	0.0000	0.0000	0.0000	-0.0000	0.0000
05_water_hexamer_1	M06_2X	cluster_4	0.0000	0.0000	0.0000	-0.0000	-0.0000
05_water_hexamer_1	M06_2X	cluster_5	-0.0000	-0.0000	-0.0000	-0.0000	-0.0000
05_water_hexamer_1	M06_2X	cluster_6	0.0000	0.0000	0.0000	0.0000	0.0000
05_water_hexamer_1	wB97XD	cluster_1	-0.0000	-0.0000	-0.0000	0.0000	0.0000
05_water_hexamer_1	wB97XD	cluster_2	0.0000	0.0000	-0.0000	0.0000	-0.0000
05_water_hexamer_1	wB97XD	cluster_3	-0.0000	-0.0000	0.0000	-0.0000	0.0000
05_water_hexamer_1	wB97XD	cluster_4	-0.0000	-0.0000	-0.0000	-0.0000	0.0000
05_water_hexamer_1	wB97XD	cluster_5	0.0000	0.0000	-0.0000	-0.0000	-0.0000
05_water_hexamer_1	wB97XD	cluster_6	-0.0000	-0.0000	0.0000	0.0000	0.0000
06_water_hexamer_2	B3LYP_D3BJ	cluster_1	0.0065	0.0065	-0.0124	0.0000	-0.0189
06_water_hexamer_2	B3LYP_D3BJ	cluster_2	-0.0153	-0.0153	0.0112	0.0000	0.0266
06_water_hexamer_2	B3LYP_D3BJ	cluster_3	0.0318	0.0318	-0.0105	-0.0000	-0.0424
06_water_hexamer_2	B3LYP_D3BJ	cluster_4	-0.0526	-0.0526	0.0037	-0.0000	0.0563
06_water_hexamer_2	B3LYP_D3BJ	cluster_5	0.0332	0.0332	-0.0022	0.0000	-0.0354
06_water_hexamer_2	B3LYP_D3BJ	cluster_6	-0.0036	-0.0036	0.0102	-0.0000	0.0137
06_water_hexamer_2	M06_2X	cluster_1	0.0065	0.0065	-0.0127	0.0000	-0.0192
06_water_hexamer_2	M06_2X	cluster_2	-0.0135	-0.0135	0.0130	0.0000	0.0265
06_water_hexamer_2	M06_2X	cluster_3	0.0302	0.0302	-0.0123	-0.0000	-0.0424
06_water_hexamer_2	M06_2X	cluster_4	-0.0521	-0.0521	0.0044	-0.0000	0.0565
06_water_hexamer_2	M06_2X	cluster_5	0.0326	0.0326	-0.0030	0.0000	-0.0356
06_water_hexamer_2	M06_2X	cluster_6	-0.0037	-0.0037	0.0106	0.0000	0.0143
06_water_hexamer_2	wB97XD	cluster_1	0.0064	0.0064	-0.0126	0.0000	-0.0190
06_water_hexamer_2	wB97XD	cluster_2	-0.0152	-0.0152	0.0116	-0.0000	0.0269
06_water_hexamer_2	wB97XD	cluster_3	0.0321	0.0321	-0.0108	0.0000	-0.0429
06_water_hexamer_2	wB97XD	cluster_4	-0.0529	-0.0529	0.0040	0.0000	0.0569
06_water_hexamer_2	wB97XD	cluster_5	0.0331	0.0331	-0.0027	-0.0000	-0.0358
06_water_hexamer_2	wB97XD	cluster_6	-0.0035	-0.0035	0.0104	-0.0000	0.0140
07_water_hexamer_3	B3LYP_D3BJ	cluster_1	0.0375	0.0375	-0.0039	0.0000	-0.0414
07_water_hexamer_3	B3LYP_D3BJ	cluster_2	-0.0306	-0.0306	0.0056	0.0000	0.0362
07_water_hexamer_3	B3LYP_D3BJ	cluster_3	-0.0393	-0.0393	0.0084	-0.0000	0.0478
07_water_hexamer_3	B3LYP_D3BJ	cluster_4	0.0381	0.0381	-0.0075	0.0000	-0.0457
07_water_hexamer_3	B3LYP_D3BJ	cluster_5	0.0117	0.0117	0.0051	-0.0000	-0.0066
07_water_hexamer_3	B3LYP_D3BJ	cluster_6	-0.0175	-0.0175	-0.0078	-0.0000	0.0097
07_water_hexamer_3	M06_2X	cluster_1	0.0372	0.0372	-0.0047	0.0000	-0.0419
07_water_hexamer_3	M06_2X	cluster_2	-0.0306	-0.0306	0.0064	-0.0000	0.0370
07_water_hexamer_3	M06_2X	cluster_3	-0.0380	-0.0380	0.0098	0.0000	0.0478
07_water_hexamer_3	M06_2X	cluster_4	0.0365	0.0365	-0.0089	0.0000	-0.0454
07_water_hexamer_3	M06_2X	cluster_5	0.0114	0.0114	0.0049	-0.0000	-0.0065
07_water_hexamer_3	M06_2X	cluster_6	-0.0165	-0.0165	-0.0075	0.0000	0.0090

Continued on the next page

Table S1: Comparison of Hirshfeld charges obtained from the sum of atomic charges, fragments defined from atomic densities, and fragments defined through WFX files for water clusters. All calculations were obtained with the 6-311++G** basis set (continued).

System	Functional	Fragment	q_{sum}	q_{atomic}	q_{WFX}	Δ_{atomic}	Δ_{WFX}
07_water_hexamer_3	wB97XD	cluster_1	0.0377	0.0377	-0.0043	0.0000	-0.0420
07_water_hexamer_3	wB97XD	cluster_2	-0.0309	-0.0309	0.0060	-0.0000	0.0369
07_water_hexamer_3	wB97XD	cluster_3	-0.0396	-0.0396	0.0087	-0.0000	0.0482
07_water_hexamer_3	wB97XD	cluster_4	0.0381	0.0381	-0.0079	0.0000	-0.0459
07_water_hexamer_3	wB97XD	cluster_5	0.0117	0.0117	0.0050	-0.0000	-0.0067
07_water_hexamer_3	wB97XD	cluster_6	-0.0170	-0.0170	-0.0076	-0.0000	0.0095
08_water_hexamer_4	B3LYP_D3BJ	cluster_1	0.0510	0.0510	-0.0020	-0.0000	-0.0530
08_water_hexamer_4	B3LYP_D3BJ	cluster_2	0.0047	0.0047	0.0022	-0.0000	-0.0025
08_water_hexamer_4	B3LYP_D3BJ	cluster_3	-0.0034	-0.0034	-0.0022	0.0000	0.0012
08_water_hexamer_4	B3LYP_D3BJ	cluster_4	-0.0058	-0.0058	0.0006	0.0000	0.0064
08_water_hexamer_4	B3LYP_D3BJ	cluster_5	0.0038	0.0038	-0.0003	0.0000	-0.0041
08_water_hexamer_4	B3LYP_D3BJ	cluster_6	-0.0503	-0.0503	0.0017	0.0000	0.0520
08_water_hexamer_4	M06_2X	cluster_1	0.0495	0.0495	-0.0034	-0.0000	-0.0529
08_water_hexamer_4	M06_2X	cluster_2	0.0048	0.0048	0.0022	0.0000	-0.0026
08_water_hexamer_4	M06_2X	cluster_3	-0.0032	-0.0032	-0.0020	0.0000	0.0012
08_water_hexamer_4	M06_2X	cluster_4	-0.0058	-0.0058	0.0006	0.0000	0.0064
08_water_hexamer_4	M06_2X	cluster_5	0.0036	0.0036	-0.0003	0.0000	-0.0039
08_water_hexamer_4	M06_2X	cluster_6	-0.0489	-0.0489	0.0029	-0.0000	0.0518
08_water_hexamer_4	wB97XD	cluster_1	0.0511	0.0511	-0.0024	0.0000	-0.0535
08_water_hexamer_4	wB97XD	cluster_2	0.0048	0.0048	0.0022	0.0000	-0.0026
08_water_hexamer_4	wB97XD	cluster_3	-0.0032	-0.0032	-0.0020	-0.0000	0.0012
08_water_hexamer_4	wB97XD	cluster_4	-0.0057	-0.0057	0.0006	0.0000	0.0064
08_water_hexamer_4	wB97XD	cluster_5	0.0038	0.0038	-0.0003	0.0000	-0.0041
08_water_hexamer_4	wB97XD	cluster_6	-0.0508	-0.0508	0.0019	-0.0000	0.0527
09_water_hexamer_5	B3LYP_D3BJ	cluster_1	-0.0083	-0.0083	-0.0031	0.0000	0.0052
09_water_hexamer_5	B3LYP_D3BJ	cluster_2	-0.0451	-0.0451	0.0139	-0.0000	0.0590
09_water_hexamer_5	B3LYP_D3BJ	cluster_3	-0.0083	-0.0083	-0.0031	0.0000	0.0052
09_water_hexamer_5	B3LYP_D3BJ	cluster_4	0.0086	0.0086	0.0026	-0.0000	-0.0060
09_water_hexamer_5	B3LYP_D3BJ	cluster_5	0.0445	0.0445	-0.0129	0.0000	-0.0574
09_water_hexamer_5	B3LYP_D3BJ	cluster_6	0.0086	0.0086	0.0026	-0.0000	-0.0060
09_water_hexamer_5	M06_2X	cluster_1	-0.0080	-0.0080	-0.0028	0.0000	0.0052
09_water_hexamer_5	M06_2X	cluster_2	-0.0420	-0.0420	0.0161	-0.0000	0.0581
09_water_hexamer_5	M06_2X	cluster_3	-0.0080	-0.0080	-0.0028	0.0000	0.0052
09_water_hexamer_5	M06_2X	cluster_4	0.0085	0.0085	0.0024	0.0000	-0.0061
09_water_hexamer_5	M06_2X	cluster_5	0.0410	0.0410	-0.0153	-0.0000	-0.0563
09_water_hexamer_5	M06_2X	cluster_6	0.0085	0.0085	0.0024	-0.0000	-0.0061
09_water_hexamer_5	wB97XD	cluster_1	-0.0081	-0.0081	-0.0029	-0.0000	0.0052
09_water_hexamer_5	wB97XD	cluster_2	-0.0456	-0.0456	0.0139	0.0000	0.0594
09_water_hexamer_5	wB97XD	cluster_3	-0.0081	-0.0081	-0.0029	0.0000	0.0052
09_water_hexamer_5	wB97XD	cluster_4	0.0087	0.0087	0.0026	-0.0000	-0.0061
09_water_hexamer_5	wB97XD	cluster_5	0.0444	0.0444	-0.0132	-0.0000	-0.0576
09_water_hexamer_5	wB97XD	cluster_6	0.0087	0.0087	0.0026	-0.0000	-0.0061

2 S66 set/6-311++G**

Table S2: Comparison of Hirshfeld charges obtained from the sum of atomic charges, fragments defined from atomic densities, and fragments defined through WFX files. All calculations were obtained with the 6-311++G** basis set.

System	Functional	Fragment	q_{sum}	q_{atomic}	q_{WFX}	Δ_{atomic}	Δ_{WFX}
01_Water-Water	B3LYP_D3BJ	Water_A	-0.0934	-0.0934	-0.0144	-0.0000	0.0790
01_Water-Water	B3LYP_D3BJ	Water_B	0.0934	0.0934	0.0144	0.0000	-0.0790
01_Water-Water	M06_2X	Water_A	-0.0909	-0.0909	-0.0125	-0.0000	0.0784
01_Water-Water	M06_2X	Water_B	0.0909	0.0909	0.0125	0.0000	-0.0784
01_Water-Water	wB97XD	Water_A	-0.0931	-0.0931	-0.0135	-0.0000	0.0795
01_Water-Water	wB97XD	Water_B	0.0931	0.0931	0.0135	-0.0000	-0.0795
02_Water-MeOH	B3LYP_D3BJ	Water_A	-0.0914	-0.0914	-0.0190	-0.0000	0.0724
02_Water-MeOH	B3LYP_D3BJ	MeOH_B	0.0914	0.0914	0.0190	-0.0000	-0.0724
02_Water-MeOH	M06_2X	Water_A	-0.0894	-0.0894	-0.0161	0.0000	0.0732
02_Water-MeOH	M06_2X	MeOH_B	0.0894	0.0894	0.0161	-0.0000	-0.0732
02_Water-MeOH	wB97XD	Water_A	-0.0915	-0.0915	-0.0177	-0.0000	0.0738
02_Water-MeOH	wB97XD	MeOH_B	0.0915	0.0915	0.0177	-0.0000	-0.0738
03_Water-MeNH2	B3LYP_D3BJ	Water_A	-0.1295	-0.1295	-0.0238	-0.0000	0.1056
03_Water-MeNH2	B3LYP_D3BJ	MeNH2_B	0.1295	0.1295	0.0238	-0.0000	-0.1056
03_Water-MeNH2	M06_2X	Water_A	-0.1265	-0.1265	-0.0204	-0.0000	0.1061
03_Water-MeNH2	M06_2X	MeNH2_B	0.1265	0.1265	0.0204	-0.0000	-0.1061
03_Water-MeNH2	wB97XD	Water_A	-0.1284	-0.1284	-0.0216	0.0000	0.1068
03_Water-MeNH2	wB97XD	MeNH2_B	0.1284	0.1284	0.0216	-0.0000	-0.1068
04_Water-Peptide	B3LYP_D3BJ	Water_A	-0.0967	-0.0967	-0.0256	0.0000	0.0711
04_Water-Peptide	B3LYP_D3BJ	Peptide_B	0.0967	0.0967	0.0256	-0.0000	-0.0711
04_Water-Peptide	M06_2X	Water_A	-0.0944	-0.0944	-0.0216	0.0000	0.0728
04_Water-Peptide	M06_2X	Peptide_B	0.0944	0.0944	0.0216	-0.0000	-0.0728
04_Water-Peptide	wB97XD	Water_A	-0.0965	-0.0965	-0.0235	0.0000	0.0729
04_Water-Peptide	wB97XD	Peptide_B	0.0965	0.0965	0.0235	-0.0000	-0.0729
05_MeOH-MeOH	B3LYP_D3BJ	MeOH_A	-0.0976	-0.0976	-0.0202	0.0000	0.0774
05_MeOH-MeOH	B3LYP_D3BJ	MeOH_B	0.0976	0.0976	0.0202	0.0000	-0.0774
05_MeOH-MeOH	M06_2X	MeOH_A	-0.0954	-0.0954	-0.0176	0.0000	0.0777
05_MeOH-MeOH	M06_2X	MeOH_B	0.0954	0.0954	0.0176	0.0000	-0.0777
05_MeOH-MeOH	wB97XD	MeOH_A	-0.0974	-0.0974	-0.0189	0.0000	0.0785
05_MeOH-MeOH	wB97XD	MeOH_B	0.0974	0.0974	0.0189	0.0000	-0.0785
06_MeOH-MeNH2	B3LYP_D3BJ	MeOH_A	-0.1359	-0.1359	-0.0268	0.0000	0.1091
06_MeOH-MeNH2	B3LYP_D3BJ	MeNH2_B	0.1359	0.1359	0.0269	0.0000	-0.1091
06_MeOH-MeNH2	M06_2X	MeOH_A	-0.1333	-0.1333	-0.0239	-0.0000	0.1094
06_MeOH-MeNH2	M06_2X	MeNH2_B	0.1333	0.1333	0.0239	0.0000	-0.1094
06_MeOH-MeNH2	wB97XD	MeOH_A	-0.1350	-0.1350	-0.0247	-0.0000	0.1102
06_MeOH-MeNH2	wB97XD	MeNH2_B	0.1350	0.1350	0.0247	0.0000	-0.1102
07_MeOH-Peptide	B3LYP_D3BJ	MeOH_A	-0.0998	-0.0998	-0.0258	-0.0000	0.0741
07_MeOH-Peptide	B3LYP_D3BJ	Peptide_B	0.0998	0.0998	0.0257	0.0000	-0.0741
07_MeOH-Peptide	M06_2X	MeOH_A	-0.0978	-0.0978	-0.0225	0.0000	0.0753
07_MeOH-Peptide	M06_2X	Peptide_B	0.0978	0.0978	0.0225	-0.0000	-0.0753
07_MeOH-Peptide	wB97XD	MeOH_A	-0.0995	-0.0995	-0.0240	0.0000	0.0755

Continued on the next page

Table S2: Comparison of Hirshfeld charges obtained from the sum of atomic charges, fragments defined from atomic densities, and fragments defined through WFX files. All calculations were obtained with the 6-311++G** basis set (continued).

System	Functional	Fragment	q_{sum}	q_{atomic}	q_{WFX}	Δ_{atomic}	Δ_{WFX}
07_MeOH-Peptide	wB97XD	Peptide_B	0.0995	0.0995	0.0240	-0.0000	-0.0755
08_MeOH-Water	B3LYP_D3BJ	MeOH_A	-0.0974	-0.0974	-0.0150	0.0000	0.0823
08_MeOH-Water	B3LYP_D3BJ	Water_B	0.0974	0.0974	0.0150	-0.0000	-0.0823
08_MeOH-Water	M06_2X	MeOH_A	-0.0948	-0.0948	-0.0133	0.0000	0.0815
08_MeOH-Water	M06_2X	Water_B	0.0948	0.0948	0.0133	-0.0000	-0.0815
08_MeOH-Water	wB97XD	MeOH_A	-0.0969	-0.0969	-0.0142	-0.0000	0.0827
08_MeOH-Water	wB97XD	Water_B	0.0969	0.0969	0.0142	0.0000	-0.0827
09_MeNH2-MeOH	B3LYP_D3BJ	MeNH2_A	-0.0560	-0.0560	-0.0095	-0.0000	0.0465
09_MeNH2-MeOH	B3LYP_D3BJ	MeOH_B	0.0560	0.0560	0.0095	0.0000	-0.0465
09_MeNH2-MeOH	M06_2X	MeNH2_A	-0.0544	-0.0544	-0.0081	0.0000	0.0463
09_MeNH2-MeOH	M06_2X	MeOH_B	0.0544	0.0544	0.0081	-0.0000	-0.0463
09_MeNH2-MeOH	wB97XD	MeNH2_A	-0.0559	-0.0559	-0.0089	0.0000	0.0471
09_MeNH2-MeOH	wB97XD	MeOH_B	0.0559	0.0559	0.0089	0.0000	-0.0471
10_MeNH2-MeNH2	B3LYP_D3BJ	MeNH2_A	-0.0568	-0.0568	-0.0104	-0.0000	0.0464
10_MeNH2-MeNH2	B3LYP_D3BJ	MeNH2_B	0.0568	0.0568	0.0104	-0.0000	-0.0464
10_MeNH2-MeNH2	M06_2X	MeNH2_A	-0.0554	-0.0554	-0.0086	-0.0000	0.0468
10_MeNH2-MeNH2	M06_2X	MeNH2_B	0.0554	0.0554	0.0086	-0.0000	-0.0468
10_MeNH2-MeNH2	wB97XD	MeNH2_A	-0.0565	-0.0565	-0.0093	0.0000	0.0472
10_MeNH2-MeNH2	wB97XD	MeNH2_B	0.0565	0.0565	0.0093	-0.0000	-0.0472
11_MeNH2-Peptide	B3LYP_D3BJ	MeNH2_A	-0.0275	-0.0275	-0.0045	-0.0000	0.0230
11_MeNH2-Peptide	B3LYP_D3BJ	Peptide_B	0.0275	0.0275	0.0045	-0.0000	-0.0230
11_MeNH2-Peptide	M06_2X	MeNH2_A	-0.0281	-0.0281	-0.0035	0.0000	0.0246
11_MeNH2-Peptide	M06_2X	Peptide_B	0.0281	0.0281	0.0035	-0.0000	-0.0246
11_MeNH2-Peptide	wB97XD	MeNH2_A	-0.0280	-0.0280	-0.0040	-0.0000	0.0239
11_MeNH2-Peptide	wB97XD	Peptide_B	0.0280	0.0280	0.0040	-0.0000	-0.0239
12_MeNH2-Water	B3LYP_D3BJ	MeNH2_A	0.1214	0.1214	0.0256	-0.0000	-0.0958
12_MeNH2-Water	B3LYP_D3BJ	Water_B	-0.1214	-0.1214	-0.0256	0.0000	0.0958
12_MeNH2-Water	M06_2X	MeNH2_A	0.1189	0.1189	0.0219	0.0000	-0.0970
12_MeNH2-Water	M06_2X	Water_B	-0.1189	-0.1189	-0.0219	-0.0000	0.0970
12_MeNH2-Water	wB97XD	MeNH2_A	0.1206	0.1206	0.0232	0.0000	-0.0975
12_MeNH2-Water	wB97XD	Water_B	-0.1206	-0.1206	-0.0232	0.0000	0.0975
13_Peptide-MeOH	B3LYP_D3BJ	Peptide_A	-0.0842	-0.0842	-0.0174	-0.0000	0.0668
13_Peptide-MeOH	B3LYP_D3BJ	MeOH_B	0.0842	0.0842	0.0174	-0.0000	-0.0668
13_Peptide-MeOH	M06_2X	Peptide_A	-0.0822	-0.0822	-0.0155	-0.0000	0.0667
13_Peptide-MeOH	M06_2X	MeOH_B	0.0822	0.0822	0.0155	-0.0000	-0.0667
13_Peptide-MeOH	wB97XD	Peptide_A	-0.0845	-0.0845	-0.0166	0.0000	0.0678
13_Peptide-MeOH	wB97XD	MeOH_B	0.0845	0.0845	0.0166	-0.0000	-0.0678
14_Peptide-MeNH2	B3LYP_D3BJ	Peptide_A	-0.1108	-0.1108	-0.0232	0.0000	0.0877
14_Peptide-MeNH2	B3LYP_D3BJ	MeNH2_B	0.1108	0.1108	0.0232	-0.0000	-0.0877
14_Peptide-MeNH2	M06_2X	Peptide_A	-0.1082	-0.1082	-0.0204	-0.0000	0.0878
14_Peptide-MeNH2	M06_2X	MeNH2_B	0.1082	0.1082	0.0204	0.0000	-0.0878
14_Peptide-MeNH2	wB97XD	Peptide_A	-0.1104	-0.1104	-0.0214	-0.0000	0.0891
14_Peptide-MeNH2	wB97XD	MeNH2_B	0.1104	0.1104	0.0214	-0.0000	-0.0891
15_Peptide-Peptide	B3LYP_D3BJ	Peptide_A	-0.0987	-0.0987	-0.0255	-0.0000	0.0732

Continued on the next page

Table S2: Comparison of Hirshfeld charges obtained from the sum of atomic charges, fragments defined from atomic densities, and fragments defined through WFX files. All calculations were obtained with the 6-311++G** basis set (continued).

System	Functional	Fragment	q_{sum}	q_{atomic}	q_{WFX}	Δ_{atomic}	Δ_{WFX}
15_Peptide-Peptide	B3LYP_D3BJ	Peptide_B	0.0987	0.0987	0.0255	-0.0000	-0.0732
15_Peptide-Peptide	M06_2X	Peptide_A	-0.0959	-0.0959	-0.0228	-0.0000	0.0730
15_Peptide-Peptide	M06_2X	Peptide_B	0.0959	0.0959	0.0228	-0.0000	-0.0730
15_Peptide-Peptide	wB97XD	Peptide_A	-0.0984	-0.0984	-0.0243	-0.0000	0.0741
15_Peptide-Peptide	wB97XD	Peptide_B	0.0984	0.0984	0.0243	-0.0000	-0.0741
16_Peptide-Water	B3LYP_D3BJ	Peptide_A	-0.0862	-0.0862	-0.0106	-0.0000	0.0756
16_Peptide-Water	B3LYP_D3BJ	Water_B	0.0862	0.0862	0.0106	-0.0000	-0.0756
16_Peptide-Water	M06_2X	Peptide_A	-0.0846	-0.0846	-0.0098	-0.0000	0.0748
16_Peptide-Water	M06_2X	Water_B	0.0846	0.0846	0.0098	-0.0000	-0.0748
16_Peptide-Water	wB97XD	Peptide_A	-0.0863	-0.0863	-0.0103	-0.0000	0.0760
16_Peptide-Water	wB97XD	Water_B	0.0863	0.0863	0.0103	0.0000	-0.0760
17_Uracil-Uracil_BP	B3LYP_D3BJ	Uracil_A	0.0182	0.0182	0.0063	-0.0000	-0.0119
17_Uracil-Uracil_BP	B3LYP_D3BJ	Uracil_B	-0.0182	-0.0182	-0.0063	-0.0000	0.0119
17_Uracil-Uracil_BP	M06_2X	Uracil_A	0.0173	0.0173	0.0062	-0.0000	-0.0112
17_Uracil-Uracil_BP	M06_2X	Uracil_B	-0.0173	-0.0173	-0.0062	-0.0000	0.0112
17_Uracil-Uracil_BP	wB97XD	Uracil_A	0.0179	0.0179	0.0062	-0.0000	-0.0117
17_Uracil-Uracil_BP	wB97XD	Uracil_B	-0.0179	-0.0179	-0.0062	-0.0000	0.0117
18_Water-Pyridine	B3LYP_D3BJ	Water_A	-0.1234	-0.1234	-0.0285	-0.0000	0.0949
18_Water-Pyridine	B3LYP_D3BJ	Pyridine_B	0.1234	0.1234	0.0285	-0.0000	-0.0949
18_Water-Pyridine	M06_2X	Water_A	-0.1209	-0.1209	-0.0246	-0.0000	0.0964
18_Water-Pyridine	M06_2X	Pyridine_B	0.1210	0.1210	0.0246	0.0000	-0.0964
18_Water-Pyridine	wB97XD	Water_A	-0.1229	-0.1229	-0.0258	0.0000	0.0971
18_Water-Pyridine	wB97XD	Pyridine_B	0.1229	0.1229	0.0258	-0.0000	-0.0971
19_MeOH-Pyridine	B3LYP_D3BJ	MeOH_A	-0.1344	-0.1344	-0.0314	0.0000	0.1030
19_MeOH-Pyridine	B3LYP_D3BJ	Pyridine_B	0.1344	0.1344	0.0314	0.0000	-0.1030
19_MeOH-Pyridine	M06_2X	MeOH_A	-0.1320	-0.1320	-0.0279	0.0000	0.1041
19_MeOH-Pyridine	M06_2X	Pyridine_B	0.1320	0.1320	0.0279	0.0000	-0.1041
19_MeOH-Pyridine	wB97XD	MeOH_A	-0.1338	-0.1338	-0.0289	0.0000	0.1049
19_MeOH-Pyridine	wB97XD	Pyridine_B	0.1338	0.1338	0.0289	-0.0000	-0.1049
20_AcOH-AcOH	B3LYP_D3BJ	AcOH_A	0.0000	0.0000	0.0000	-0.0000	0.0000
20_AcOH-AcOH	B3LYP_D3BJ	AcOH_B	-0.0000	-0.0000	-0.0000	0.0000	-0.0000
20_AcOH-AcOH	M06_2X	AcOH_A	0.0000	0.0000	0.0000	-0.0000	0.0000
20_AcOH-AcOH	M06_2X	AcOH_B	-0.0000	-0.0000	-0.0000	-0.0000	-0.0000
20_AcOH-AcOH	wB97XD	AcOH_A	0.0000	0.0000	0.0000	-0.0000	0.0000
20_AcOH-AcOH	wB97XD	AcOH_B	-0.0000	-0.0000	-0.0000	-0.0000	-0.0000
21_AcNH2-AcNH2	B3LYP_D3BJ	AcNH2_A	-0.0000	-0.0000	-0.0000	-0.0000	0.0000
21_AcNH2-AcNH2	B3LYP_D3BJ	AcNH2_B	0.0000	0.0000	0.0000	0.0000	-0.0000
21_AcNH2-AcNH2	M06_2X	AcNH2_A	-0.0000	-0.0000	-0.0000	-0.0000	0.0000
21_AcNH2-AcNH2	M06_2X	AcNH2_B	0.0000	0.0000	0.0000	-0.0000	-0.0000
21_AcNH2-AcNH2	wB97XD	AcNH2_A	-0.0000	-0.0000	-0.0000	0.0000	0.0000
21_AcNH2-AcNH2	wB97XD	AcNH2_B	0.0000	0.0000	0.0000	0.0000	-0.0000
22_AcOH-Uracil	B3LYP_D3BJ	AcOH_A	-0.0203	-0.0203	-0.0107	-0.0000	0.0096
22_AcOH-Uracil	B3LYP_D3BJ	Uracil_B	0.0203	0.0203	0.0107	-0.0000	-0.0096
22_AcOH-Uracil	M06_2X	AcOH_A	-0.0208	-0.0208	-0.0095	-0.0000	0.0113

Continued on the next page

Table S2: Comparison of Hirshfeld charges obtained from the sum of atomic charges, fragments defined from atomic densities, and fragments defined through WFX files. All calculations were obtained with the 6-311++G** basis set (continued).

System	Functional	Fragment	q_{sum}	q_{atomic}	q_{WFX}	Δ_{atomic}	Δ_{WFX}
22_AcOH-Uracil	M06_2X	Uracil_B	0.0208	0.0208	0.0095	0.0000	-0.0113
22_AcOH-Uracil	wB97XD	AcOH_A	-0.0201	-0.0201	-0.0096	-0.0000	0.0104
22_AcOH-Uracil	wB97XD	Uracil_B	0.0201	0.0201	0.0096	-0.0000	-0.0104
23_AcNH2-Uracil	B3LYP_D3BJ	AcNH2_A	0.0484	0.0484	0.0183	0.0000	-0.0301
23_AcNH2-Uracil	B3LYP_D3BJ	Uracil_B	-0.0484	-0.0484	-0.0183	0.0000	0.0301
23_AcNH2-Uracil	M06_2X	AcNH2_A	0.0478	0.0478	0.0181	0.0000	-0.0297
23_AcNH2-Uracil	M06_2X	Uracil_B	-0.0478	-0.0478	-0.0181	0.0000	0.0297
23_AcNH2-Uracil	wB97XD	AcNH2_A	0.0479	0.0479	0.0178	-0.0000	-0.0302
23_AcNH2-Uracil	wB97XD	Uracil_B	-0.0479	-0.0479	-0.0178	0.0000	0.0302
24_Benzene-Benzene_pi-pi	B3LYP_D3BJ	Benzene_A	0.0000	0.0000	0.0000	-0.0000	-0.0000
24_Benzene-Benzene_pi-pi	B3LYP_D3BJ	Benzene_B	-0.0000	-0.0000	-0.0000	-0.0000	0.0000
24_Benzene-Benzene_pi-pi	M06_2X	Benzene_A	0.0000	0.0000	0.0000	-0.0000	-0.0000
24_Benzene-Benzene_pi-pi	M06_2X	Benzene_B	-0.0000	-0.0000	-0.0000	-0.0000	0.0000
24_Benzene-Benzene_pi-pi	wB97XD	Benzene_A	0.0000	0.0000	-0.0000	-0.0000	-0.0000
24_Benzene-Benzene_pi-pi	wB97XD	Benzene_B	-0.0000	-0.0000	-0.0000	-0.0000	0.0000
25_Pyridine-Pyridine_pi-pi	B3LYP_D3BJ	Pyridine_A	-0.0002	-0.0002	-0.0001	-0.0000	0.0002
25_Pyridine-Pyridine_pi-pi	B3LYP_D3BJ	Pyridine_B	0.0002	0.0002	0.0001	-0.0000	-0.0002
25_Pyridine-Pyridine_pi-pi	M06_2X	Pyridine_A	-0.0002	-0.0002	-0.0001	-0.0000	0.0002
25_Pyridine-Pyridine_pi-pi	M06_2X	Pyridine_B	0.0002	0.0002	0.0001	-0.0000	-0.0002
25_Pyridine-Pyridine_pi-pi	wB97XD	Pyridine_A	-0.0002	-0.0002	-0.0001	-0.0000	0.0002
25_Pyridine-Pyridine_pi-pi	wB97XD	Pyridine_B	0.0002	0.0002	0.0001	-0.0000	-0.0002
26_Uracil-Uracil_pi-pi	B3LYP_D3BJ	Uracil_A	-0.0000	-0.0000	-0.0000	-0.0000	-0.0000
26_Uracil-Uracil_pi-pi	B3LYP_D3BJ	Uracil_B	-0.0000	-0.0000	-0.0000	-0.0000	0.0000
26_Uracil-Uracil_pi-pi	M06_2X	Uracil_A	-0.0000	-0.0000	-0.0000	-0.0000	-0.0000
26_Uracil-Uracil_pi-pi	M06_2X	Uracil_B	-0.0000	-0.0000	-0.0000	-0.0000	0.0000
26_Uracil-Uracil_pi-pi	wB97XD	Uracil_A	-0.0000	-0.0000	-0.0000	-0.0000	-0.0000
26_Uracil-Uracil_pi-pi	wB97XD	Uracil_B	-0.0000	-0.0000	-0.0000	-0.0000	0.0000
27_Benzene-Pyridine_pi-pi	B3LYP_D3BJ	Benzene_A	0.0071	0.0071	0.0016	0.0000	-0.0055
27_Benzene-Pyridine_pi-pi	B3LYP_D3BJ	Pyridine_B	-0.0071	-0.0071	-0.0016	0.0000	0.0055
27_Benzene-Pyridine_pi-pi	M06_2X	Benzene_A	0.0069	0.0069	0.0014	-0.0000	-0.0055
27_Benzene-Pyridine_pi-pi	M06_2X	Pyridine_B	-0.0069	-0.0069	-0.0014	-0.0000	0.0055
27_Benzene-Pyridine_pi-pi	wB97XD	Benzene_A	0.0066	0.0066	0.0011	-0.0000	-0.0054
27_Benzene-Pyridine_pi-pi	wB97XD	Pyridine_B	-0.0066	-0.0066	-0.0011	-0.0000	0.0054
28_Benzene-Uracil_pi-pi	B3LYP_D3BJ	Benzene_A	0.0068	0.0068	-0.0025	-0.0000	-0.0093
28_Benzene-Uracil_pi-pi	B3LYP_D3BJ	Uracil_B	-0.0068	-0.0068	0.0025	-0.0000	0.0093
28_Benzene-Uracil_pi-pi	M06_2X	Benzene_A	0.0080	0.0080	-0.0016	-0.0000	-0.0097
28_Benzene-Uracil_pi-pi	M06_2X	Uracil_B	-0.0080	-0.0080	0.0016	-0.0000	0.0097
28_Benzene-Uracil_pi-pi	wB97XD	Benzene_A	0.0064	0.0064	-0.0030	-0.0000	-0.0094
28_Benzene-Uracil_pi-pi	wB97XD	Uracil_B	-0.0064	-0.0064	0.0030	-0.0000	0.0094
29_Pyridine-Uracil_pi-pi	B3LYP_D3BJ	Pyridine_A	0.0024	0.0024	-0.0035	-0.0000	-0.0058
29_Pyridine-Uracil_pi-pi	B3LYP_D3BJ	Uracil_B	-0.0024	-0.0024	0.0035	-0.0000	0.0058
29_Pyridine-Uracil_pi-pi	M06_2X	Pyridine_A	0.0036	0.0036	-0.0021	-0.0000	-0.0056
29_Pyridine-Uracil_pi-pi	M06_2X	Uracil_B	-0.0036	-0.0036	0.0020	-0.0000	0.0056
29_Pyridine-Uracil_pi-pi	wB97XD	Pyridine_A	0.0029	0.0029	-0.0029	-0.0000	-0.0058

Continued on the next page

Table S2: Comparison of Hirshfeld charges obtained from the sum of atomic charges, fragments defined from atomic densities, and fragments defined through WFX files. All calculations were obtained with the 6-311++G** basis set (continued).

System	Functional	Fragment	q_{sum}	q_{atomic}	q_{WFX}	Δ_{atomic}	Δ_{WFX}
29_Pyridine-Uracil_pi-pi	wB97XD	Uracil_B	-0.0030	-0.0030	0.0028	-0.0000	0.0058
30_Benzene-Ethene	B3LYP_D3BJ	Benzene_A	0.0144	0.0144	0.0065	0.0000	-0.0079
30_Benzene-Ethene	B3LYP_D3BJ	Ethene_B	-0.0144	-0.0144	-0.0065	-0.0000	0.0079
30_Benzene-Ethene	M06_2X	Benzene_A	0.0145	0.0145	0.0044	-0.0000	-0.0101
30_Benzene-Ethene	M06_2X	Ethene_B	-0.0145	-0.0145	-0.0045	-0.0000	0.0101
30_Benzene-Ethene	wB97XD	Benzene_A	0.0139	0.0139	0.0047	-0.0000	-0.0092
30_Benzene-Ethene	wB97XD	Ethene_B	-0.0139	-0.0139	-0.0047	-0.0000	0.0092
31_Uracil-Ethene	B3LYP_D3BJ	Uracil_A	0.0077	0.0077	0.0033	0.0000	-0.0043
31_Uracil-Ethene	B3LYP_D3BJ	Ethene_B	-0.0077	-0.0077	-0.0033	-0.0000	0.0043
31_Uracil-Ethene	M06_2X	Uracil_A	0.0081	0.0081	0.0008	0.0000	-0.0073
31_Uracil-Ethene	M06_2X	Ethene_B	-0.0081	-0.0081	-0.0008	-0.0000	0.0073
31_Uracil-Ethene	wB97XD	Uracil_A	0.0080	0.0080	0.0020	0.0000	-0.0061
31_Uracil-Ethene	wB97XD	Ethene_B	-0.0080	-0.0080	-0.0020	-0.0000	0.0061
32_Uracil-Ethyne	B3LYP_D3BJ	Uracil_A	-0.0030	-0.0030	0.0024	0.0000	0.0054
32_Uracil-Ethyne	B3LYP_D3BJ	Ethyne_B	0.0030	0.0030	-0.0024	-0.0000	-0.0054
32_Uracil-Ethyne	M06_2X	Uracil_A	-0.0031	-0.0031	-0.0000	0.0000	0.0031
32_Uracil-Ethyne	M06_2X	Ethyne_B	0.0031	0.0031	0.0000	-0.0000	-0.0031
32_Uracil-Ethyne	wB97XD	Uracil_A	-0.0029	-0.0029	0.0014	0.0000	0.0042
32_Uracil-Ethyne	wB97XD	Ethyne_B	0.0029	0.0029	-0.0014	-0.0000	-0.0042
33_Pyridine-Ethene	B3LYP_D3BJ	Pyridine_A	0.0098	0.0098	0.0059	0.0000	-0.0039
33_Pyridine-Ethene	B3LYP_D3BJ	Ethene_B	-0.0098	-0.0098	-0.0059	0.0000	0.0039
33_Pyridine-Ethene	M06_2X	Pyridine_A	0.0101	0.0101	0.0039	-0.0000	-0.0062
33_Pyridine-Ethene	M06_2X	Ethene_B	-0.0102	-0.0102	-0.0039	-0.0000	0.0062
33_Pyridine-Ethene	wB97XD	Pyridine_A	0.0097	0.0097	0.0044	-0.0000	-0.0054
33_Pyridine-Ethene	wB97XD	Ethene_B	-0.0097	-0.0097	-0.0044	-0.0000	0.0054
34_Pentane-Pentane	B3LYP_D3BJ	Pentane_A	-0.0000	-0.0000	-0.0000	0.0000	-0.0000
34_Pentane-Pentane	B3LYP_D3BJ	Pentane_B	-0.0000	-0.0000	-0.0000	0.0000	0.0000
34_Pentane-Pentane	M06_2X	Pentane_A	-0.0000	-0.0000	-0.0000	0.0000	-0.0000
34_Pentane-Pentane	M06_2X	Pentane_B	-0.0000	-0.0000	-0.0000	-0.0000	0.0000
34_Pentane-Pentane	wB97XD	Pentane_A	-0.0000	-0.0000	-0.0000	-0.0000	-0.0000
34_Pentane-Pentane	wB97XD	Pentane_B	-0.0000	-0.0000	-0.0000	-0.0000	0.0000
35_Neopentane-Pentane	B3LYP_D3BJ	Neopentane_A	-0.0016	-0.0016	0.0022	0.0000	0.0037
35_Neopentane-Pentane	B3LYP_D3BJ	Pentane_B	0.0015	0.0015	-0.0022	-0.0000	-0.0037
35_Neopentane-Pentane	M06_2X	Neopentane_A	-0.0015	-0.0015	0.0007	0.0000	0.0022
35_Neopentane-Pentane	M06_2X	Pentane_B	0.0014	0.0014	-0.0008	-0.0000	-0.0022
35_Neopentane-Pentane	wB97XD	Neopentane_A	-0.0014	-0.0014	0.0012	-0.0000	0.0025
35_Neopentane-Pentane	wB97XD	Pentane_B	0.0013	0.0013	-0.0012	-0.0000	-0.0025
36_Neopentane-Neopentane	B3LYP_D3BJ	Neopentane_A	0.0000	0.0000	0.0000	-0.0000	0.0000
36_Neopentane-Neopentane	B3LYP_D3BJ	Neopentane_B	0.0000	0.0000	0.0000	-0.0000	-0.0000
36_Neopentane-Neopentane	M06_2X	Neopentane_A	0.0000	0.0000	0.0000	-0.0000	0.0000
36_Neopentane-Neopentane	M06_2X	Neopentane_B	0.0000	0.0000	0.0000	-0.0000	-0.0000
36_Neopentane-Neopentane	wB97XD	Neopentane_A	0.0000	0.0000	0.0000	-0.0000	0.0000
36_Neopentane-Neopentane	wB97XD	Neopentane_B	0.0000	0.0000	0.0000	-0.0000	-0.0000
37_Cyclopentane-Neopentane	B3LYP_D3BJ	Cyclopentane_A	-0.0039	-0.0039	0.0005	-0.0000	0.0044

Continued on the next page

Table S2: Comparison of Hirshfeld charges obtained from the sum of atomic charges, fragments defined from atomic densities, and fragments defined through WFX files. All calculations were obtained with the 6-311++G** basis set (continued).

System	Functional	Fragment	q_{sum}	q_{atomic}	q_{WFX}	Δ_{atomic}	Δ_{WFX}
37_Cyclopentane-Neopentane	B3LYP_D3BJ	Neopentane_B	0.0039	0.0039	-0.0005	0.0000	-0.0044
37_Cyclopentane-Neopentane	M06_2X	Cyclopentane_A	-0.0036	-0.0036	-0.0004	0.0000	0.0032
37_Cyclopentane-Neopentane	M06_2X	Neopentane_B	0.0036	0.0036	0.0004	-0.0000	-0.0032
37_Cyclopentane-Neopentane	wB97XD	Cyclopentane_A	-0.0035	-0.0035	0.0001	-0.0000	0.0036
37_Cyclopentane-Neopentane	wB97XD	Neopentane_B	0.0035	0.0035	-0.0001	-0.0000	-0.0036
38_Cyclopentane-Cyclopentane	B3LYP_D3BJ	Cyclopentane_A	-0.0000	-0.0000	-0.0000	-0.0000	-0.0000
38_Cyclopentane-Cyclopentane	B3LYP_D3BJ	Cyclopentane_B	-0.0000	-0.0000	-0.0000	0.0000	0.0000
38_Cyclopentane-Cyclopentane	M06_2X	Cyclopentane_A	-0.0000	-0.0000	-0.0000	-0.0000	-0.0000
38_Cyclopentane-Cyclopentane	M06_2X	Cyclopentane_B	-0.0000	-0.0000	-0.0000	0.0000	0.0000
38_Cyclopentane-Cyclopentane	wB97XD	Cyclopentane_A	0.0000	0.0000	0.0000	-0.0000	-0.0000
38_Cyclopentane-Cyclopentane	wB97XD	Cyclopentane_B	-0.0000	-0.0000	-0.0000	-0.0000	0.0000
39_Benzene-Cyclopentane	B3LYP_D3BJ	Benzene_A	0.0524	0.0524	0.0043	0.0000	-0.0481
39_Benzene-Cyclopentane	B3LYP_D3BJ	Cyclopentane_B	-0.0524	-0.0524	-0.0043	-0.0000	0.0481
39_Benzene-Cyclopentane	M06_2X	Benzene_A	0.0566	0.0566	0.0039	-0.0000	-0.0527
39_Benzene-Cyclopentane	M06_2X	Cyclopentane_B	-0.0566	-0.0566	-0.0039	-0.0000	0.0527
39_Benzene-Cyclopentane	wB97XD	Benzene_A	0.0534	0.0534	0.0027	-0.0000	-0.0506
39_Benzene-Cyclopentane	wB97XD	Cyclopentane_B	-0.0534	-0.0534	-0.0028	-0.0000	0.0506
40_Benzene-Neopentane	B3LYP_D3BJ	Benzene_A	0.0451	0.0451	0.0063	0.0000	-0.0388
40_Benzene-Neopentane	B3LYP_D3BJ	Neopentane_B	-0.0451	-0.0451	-0.0063	-0.0000	0.0388
40_Benzene-Neopentane	M06_2X	Benzene_A	0.0484	0.0484	0.0046	-0.0000	-0.0437
40_Benzene-Neopentane	M06_2X	Neopentane_B	-0.0484	-0.0484	-0.0046	-0.0000	0.0437
40_Benzene-Neopentane	wB97XD	Benzene_A	0.0459	0.0459	0.0042	-0.0000	-0.0417
40_Benzene-Neopentane	wB97XD	Neopentane_B	-0.0459	-0.0459	-0.0042	-0.0000	0.0417
41_Uracil-Pentane	B3LYP_D3BJ	Uracil_A	0.0560	0.0560	0.0047	-0.0000	-0.0513
41_Uracil-Pentane	B3LYP_D3BJ	Pentane_B	-0.0561	-0.0561	-0.0047	0.0000	0.0513
41_Uracil-Pentane	M06_2X	Uracil_A	0.0601	0.0601	0.0029	-0.0000	-0.0571
41_Uracil-Pentane	M06_2X	Pentane_B	-0.0601	-0.0601	-0.0030	-0.0000	0.0571
41_Uracil-Pentane	wB97XD	Uracil_A	0.0578	0.0578	0.0035	-0.0000	-0.0543
41_Uracil-Pentane	wB97XD	Pentane_B	-0.0579	-0.0579	-0.0036	-0.0000	0.0543
42_Uracil-Cyclopentane	B3LYP_D3BJ	Uracil_A	0.0527	0.0527	0.0033	-0.0000	-0.0494
42_Uracil-Cyclopentane	B3LYP_D3BJ	Cyclopentane_B	-0.0527	-0.0527	-0.0033	0.0000	0.0494
42_Uracil-Cyclopentane	M06_2X	Uracil_A	0.0565	0.0565	0.0019	-0.0000	-0.0546
42_Uracil-Cyclopentane	M06_2X	Cyclopentane_B	-0.0565	-0.0565	-0.0019	-0.0000	0.0546
42_Uracil-Cyclopentane	wB97XD	Uracil_A	0.0543	0.0543	0.0021	-0.0000	-0.0522
42_Uracil-Cyclopentane	wB97XD	Cyclopentane_B	-0.0543	-0.0543	-0.0021	-0.0000	0.0522
43_Uracil-Neopentane	B3LYP_D3BJ	Uracil_A	0.0333	0.0333	0.0028	-0.0000	-0.0305
43_Uracil-Neopentane	B3LYP_D3BJ	Neopentane_B	-0.0333	-0.0333	-0.0028	-0.0000	0.0305
43_Uracil-Neopentane	M06_2X	Uracil_A	0.0359	0.0359	0.0010	-0.0000	-0.0349
43_Uracil-Neopentane	M06_2X	Neopentane_B	-0.0359	-0.0359	-0.0010	-0.0000	0.0349
43_Uracil-Neopentane	wB97XD	Uracil_A	0.0348	0.0348	0.0016	-0.0000	-0.0332
43_Uracil-Neopentane	wB97XD	Neopentane_B	-0.0348	-0.0348	-0.0016	-0.0000	0.0332
44_Ethene-Pentane	B3LYP_D3BJ	Ethene_A	0.0070	0.0070	-0.0010	0.0000	-0.0081
44_Ethene-Pentane	B3LYP_D3BJ	Pentane_B	-0.0070	-0.0070	0.0010	0.0000	0.0081
44_Ethene-Pentane	M06_2X	Ethene_A	0.0075	0.0075	0.0005	-0.0000	-0.0070

Continued on the next page

Table S2: Comparison of Hirshfeld charges obtained from the sum of atomic charges, fragments defined from atomic densities, and fragments defined through WFX files. All calculations were obtained with the 6-311++G** basis set (continued).

System	Functional	Fragment	q_{sum}	q_{atomic}	q_{WFX}	Δ_{atomic}	Δ_{WFX}
44_Ethene-Pentane	M06_2X	Pentane_B	-0.0075	-0.0075	-0.0005	0.0000	0.0070
44_Ethene-Pentane	wB97XD	Ethene_A	0.0071	0.0071	-0.0001	-0.0000	-0.0072
44_Ethene-Pentane	wB97XD	Pentane_B	-0.0071	-0.0071	0.0001	-0.0000	0.0072
45_Ethyne-Pentane	B3LYP_D3BJ	Ethyne_A	0.0329	0.0329	0.0004	-0.0000	-0.0326
45_Ethyne-Pentane	B3LYP_D3BJ	Pentane_B	-0.0330	-0.0330	-0.0004	0.0000	0.0326
45_Ethyne-Pentane	M06_2X	Ethyne_A	0.0346	0.0346	0.0015	-0.0000	-0.0330
45_Ethyne-Pentane	M06_2X	Pentane_B	-0.0346	-0.0346	-0.0016	0.0000	0.0330
45_Ethyne-Pentane	wB97XD	Ethyne_A	0.0332	0.0332	0.0005	-0.0000	-0.0327
45_Ethyne-Pentane	wB97XD	Pentane_B	-0.0332	-0.0332	-0.0005	-0.0000	0.0327
46_Peptide-Pentane	B3LYP_D3BJ	Peptide_A	0.0352	0.0352	0.0049	0.0000	-0.0302
46_Peptide-Pentane	B3LYP_D3BJ	Pentane_B	-0.0352	-0.0352	-0.0049	0.0000	0.0302
46_Peptide-Pentane	M06_2X	Peptide_A	0.0370	0.0370	0.0046	-0.0000	-0.0324
46_Peptide-Pentane	M06_2X	Pentane_B	-0.0370	-0.0370	-0.0046	-0.0000	0.0324
46_Peptide-Pentane	wB97XD	Peptide_A	0.0361	0.0361	0.0049	-0.0000	-0.0312
46_Peptide-Pentane	wB97XD	Pentane_B	-0.0361	-0.0361	-0.0049	-0.0000	0.0312
47_Benzene-Benzene_TS	B3LYP_D3BJ	Benzene_A	0.0459	0.0459	0.0050	-0.0000	-0.0408
47_Benzene-Benzene_TS	B3LYP_D3BJ	Benzene_B	-0.0459	-0.0459	-0.0050	-0.0000	0.0408
47_Benzene-Benzene_TS	M06_2X	Benzene_A	0.0488	0.0488	0.0036	-0.0000	-0.0452
47_Benzene-Benzene_TS	M06_2X	Benzene_B	-0.0488	-0.0488	-0.0036	-0.0000	0.0452
47_Benzene-Benzene_TS	wB97XD	Benzene_A	0.0465	0.0465	0.0030	-0.0000	-0.0434
47_Benzene-Benzene_TS	wB97XD	Benzene_B	-0.0465	-0.0465	-0.0030	-0.0000	0.0434
48_Pyridine-Pyridine_TS	B3LYP_D3BJ	Pyridine_A	0.0577	0.0577	0.0073	-0.0000	-0.0504
48_Pyridine-Pyridine_TS	B3LYP_D3BJ	Pyridine_B	-0.0577	-0.0577	-0.0073	-0.0000	0.0504
48_Pyridine-Pyridine_TS	M06_2X	Pyridine_A	0.0602	0.0602	0.0058	-0.0000	-0.0544
48_Pyridine-Pyridine_TS	M06_2X	Pyridine_B	-0.0602	-0.0602	-0.0058	-0.0000	0.0544
48_Pyridine-Pyridine_TS	wB97XD	Pyridine_A	0.0586	0.0586	0.0056	-0.0000	-0.0531
48_Pyridine-Pyridine_TS	wB97XD	Pyridine_B	-0.0586	-0.0586	-0.0056	-0.0000	0.0531
49_Benzene-Pyridine_TS	B3LYP_D3BJ	Benzene_A	0.0524	0.0524	0.0068	-0.0000	-0.0456
49_Benzene-Pyridine_TS	B3LYP_D3BJ	Pyridine_B	-0.0524	-0.0524	-0.0068	-0.0000	0.0456
49_Benzene-Pyridine_TS	M06_2X	Benzene_A	0.0552	0.0552	0.0052	-0.0000	-0.0500
49_Benzene-Pyridine_TS	M06_2X	Pyridine_B	-0.0553	-0.0553	-0.0052	-0.0000	0.0500
49_Benzene-Pyridine_TS	wB97XD	Benzene_A	0.0529	0.0529	0.0046	-0.0000	-0.0483
49_Benzene-Pyridine_TS	wB97XD	Pyridine_B	-0.0529	-0.0529	-0.0046	-0.0000	0.0483
50_Benzene-Ethyne_CH-pi	B3LYP_D3BJ	Benzene_A	0.0537	0.0537	0.0080	-0.0000	-0.0457
50_Benzene-Ethyne_CH-pi	B3LYP_D3BJ	Ethyne_B	-0.0537	-0.0537	-0.0080	0.0000	0.0457
50_Benzene-Ethyne_CH-pi	M06_2X	Benzene_A	0.0554	0.0554	0.0059	-0.0000	-0.0494
50_Benzene-Ethyne_CH-pi	M06_2X	Ethyne_B	-0.0554	-0.0554	-0.0060	-0.0000	0.0494
50_Benzene-Ethyne_CH-pi	wB97XD	Benzene_A	0.0535	0.0535	0.0056	0.0000	-0.0479
50_Benzene-Ethyne_CH-pi	wB97XD	Ethyne_B	-0.0535	-0.0535	-0.0056	0.0000	0.0479
51_Ethyne-Ethyne_TS	B3LYP_D3BJ	Ethyne_A	0.0390	0.0390	0.0044	-0.0000	-0.0347
51_Ethyne-Ethyne_TS	B3LYP_D3BJ	Ethyne_B	-0.0390	-0.0390	-0.0044	-0.0000	0.0347
51_Ethyne-Ethyne_TS	M06_2X	Ethyne_A	0.0387	0.0387	0.0035	-0.0000	-0.0352
51_Ethyne-Ethyne_TS	M06_2X	Ethyne_B	-0.0387	-0.0387	-0.0035	-0.0000	0.0352
51_Ethyne-Ethyne_TS	wB97XD	Ethyne_A	0.0387	0.0387	0.0036	0.0000	-0.0351

Continued on the next page

Table S2: Comparison of Hirshfeld charges obtained from the sum of atomic charges, fragments defined from atomic densities, and fragments defined through WFX files. All calculations were obtained with the 6-311++G** basis set (continued).

System	Functional	Fragment	q_{sum}	q_{atomic}	q_{WFX}	Δ_{atomic}	Δ_{WFX}
51_Ethyne-Ethyne_TS	wB97XD	Ethyne_B	-0.0387	-0.0387	-0.0036	-0.0000	0.0351
52_Benzene-AcOH_OH-pi	B3LYP_D3BJ	Benzene_A	0.0616	0.0616	0.0133	-0.0000	-0.0483
52_Benzene-AcOH_OH-pi	B3LYP_D3BJ	AcOH_B	-0.0616	-0.0616	-0.0133	-0.0000	0.0483
52_Benzene-AcOH_OH-pi	M06_2X	Benzene_A	0.0645	0.0645	0.0101	-0.0000	-0.0544
52_Benzene-AcOH_OH-pi	M06_2X	AcOH_B	-0.0645	-0.0645	-0.0101	-0.0000	0.0544
52_Benzene-AcOH_OH-pi	wB97XD	Benzene_A	0.0618	0.0618	0.0098	-0.0000	-0.0520
52_Benzene-AcOH_OH-pi	wB97XD	AcOH_B	-0.0618	-0.0618	-0.0098	-0.0000	0.0520
53_Benzene-AcNH2_NH-pi	B3LYP_D3BJ	Benzene_A	0.0094	0.0094	0.0047	-0.0000	-0.0047
53_Benzene-AcNH2_NH-pi	B3LYP_D3BJ	AcNH2_B	-0.0094	-0.0094	-0.0047	0.0000	0.0047
53_Benzene-AcNH2_NH-pi	M06_2X	Benzene_A	0.0104	0.0104	0.0028	-0.0000	-0.0076
53_Benzene-AcNH2_NH-pi	M06_2X	AcNH2_B	-0.0104	-0.0104	-0.0028	-0.0000	0.0076
53_Benzene-AcNH2_NH-pi	wB97XD	Benzene_A	0.0092	0.0092	0.0028	-0.0000	-0.0064
53_Benzene-AcNH2_NH-pi	wB97XD	AcNH2_B	-0.0092	-0.0092	-0.0028	-0.0000	0.0064
54_Benzene-Water_OH-pi	B3LYP_D3BJ	Benzene_A	0.0605	0.0605	0.0063	-0.0000	-0.0543
54_Benzene-Water_OH-pi	B3LYP_D3BJ	Water_B	-0.0605	-0.0605	-0.0063	0.0000	0.0543
54_Benzene-Water_OH-pi	M06_2X	Benzene_A	0.0636	0.0636	0.0040	-0.0000	-0.0596
54_Benzene-Water_OH-pi	M06_2X	Water_B	-0.0636	-0.0636	-0.0040	-0.0000	0.0596
54_Benzene-Water_OH-pi	wB97XD	Benzene_A	0.0610	0.0610	0.0037	-0.0000	-0.0573
54_Benzene-Water_OH-pi	wB97XD	Water_B	-0.0610	-0.0610	-0.0037	0.0000	0.0573
55_Benzene-MeOH_OH-pi	B3LYP_D3BJ	Benzene_A	0.0753	0.0753	0.0069	-0.0000	-0.0684
55_Benzene-MeOH_OH-pi	B3LYP_D3BJ	MeOH_B	-0.0753	-0.0753	-0.0069	-0.0000	0.0684
55_Benzene-MeOH_OH-pi	M06_2X	Benzene_A	0.0793	0.0793	0.0053	-0.0000	-0.0740
55_Benzene-MeOH_OH-pi	M06_2X	MeOH_B	-0.0793	-0.0793	-0.0053	-0.0000	0.0740
55_Benzene-MeOH_OH-pi	wB97XD	Benzene_A	0.0759	0.0759	0.0045	-0.0000	-0.0714
55_Benzene-MeOH_OH-pi	wB97XD	MeOH_B	-0.0759	-0.0759	-0.0045	-0.0000	0.0714
56_Benzene-MeNH2_NH-pi	B3LYP_D3BJ	Benzene_A	0.0554	0.0554	0.0054	-0.0000	-0.0500
56_Benzene-MeNH2_NH-pi	B3LYP_D3BJ	MeNH2_B	-0.0554	-0.0554	-0.0053	-0.0000	0.0500
56_Benzene-MeNH2_NH-pi	M06_2X	Benzene_A	0.0589	0.0589	0.0039	-0.0000	-0.0550
56_Benzene-MeNH2_NH-pi	M06_2X	MeNH2_B	-0.0589	-0.0589	-0.0039	-0.0000	0.0550
56_Benzene-MeNH2_NH-pi	wB97XD	Benzene_A	0.0563	0.0563	0.0032	-0.0000	-0.0531
56_Benzene-MeNH2_NH-pi	wB97XD	MeNH2_B	-0.0563	-0.0563	-0.0032	-0.0000	0.0531
57_Benzene-Peptide_NH-pi	B3LYP_D3BJ	Benzene_A	0.0768	0.0768	0.0107	-0.0000	-0.0661
57_Benzene-Peptide_NH-pi	B3LYP_D3BJ	Peptide_B	-0.0768	-0.0768	-0.0107	-0.0000	0.0661
57_Benzene-Peptide_NH-pi	M06_2X	Benzene_A	0.0806	0.0806	0.0090	-0.0000	-0.0716
57_Benzene-Peptide_NH-pi	M06_2X	Peptide_B	-0.0806	-0.0806	-0.0090	-0.0000	0.0716
57_Benzene-Peptide_NH-pi	wB97XD	Benzene_A	0.0776	0.0776	0.0080	-0.0000	-0.0696
57_Benzene-Peptide_NH-pi	wB97XD	Peptide_B	-0.0776	-0.0776	-0.0080	-0.0000	0.0696
58_Pyridine-Pyridine_CH-N	B3LYP_D3BJ	Pyridine_A	-0.0000	-0.0000	-0.0000	-0.0000	0.0000
58_Pyridine-Pyridine_CH-N	B3LYP_D3BJ	Pyridine_B	0.0000	0.0000	0.0000	-0.0000	-0.0000
58_Pyridine-Pyridine_CH-N	M06_2X	Pyridine_A	-0.0000	-0.0000	-0.0000	-0.0000	0.0000
58_Pyridine-Pyridine_CH-N	M06_2X	Pyridine_B	0.0000	0.0000	0.0000	-0.0000	-0.0000
58_Pyridine-Pyridine_CH-N	wB97XD	Pyridine_A	-0.0000	-0.0000	-0.0000	-0.0000	0.0000
58_Pyridine-Pyridine_CH-N	wB97XD	Pyridine_B	0.0000	0.0000	0.0000	-0.0000	-0.0000
59_Ethyne-Water_CH-O	B3LYP_D3BJ	Ethyne_A	-0.0597	-0.0597	-0.0057	0.0000	0.0540

Continued on the next page

Table S2: Comparison of Hirshfeld charges obtained from the sum of atomic charges, fragments defined from atomic densities, and fragments defined through WFX files. All calculations were obtained with the 6-311++G** basis set (continued).

System	Functional	Fragment	q_{sum}	q_{atomic}	q_{WFX}	Δ_{atomic}	Δ_{WFX}
59_Ethyne-Water_CH-O	B3LYP_D3BJ	Water_B	0.0597	0.0597	0.0057	0.0000	-0.0540
59_Ethyne-Water_CH-O	M06_2X	Ethyne_A	-0.0584	-0.0584	-0.0051	0.0000	0.0532
59_Ethyne-Water_CH-O	M06_2X	Water_B	0.0584	0.0584	0.0051	-0.0000	-0.0532
59_Ethyne-Water_CH-O	wB97XD	Ethyne_A	-0.0594	-0.0594	-0.0055	-0.0000	0.0539
59_Ethyne-Water_CH-O	wB97XD	Water_B	0.0594	0.0594	0.0055	-0.0000	-0.0539
60_Ethyne-AcOH_OH-pi	B3LYP_D3BJ	Ethyne_A	0.0450	0.0450	0.0157	-0.0000	-0.0293
60_Ethyne-AcOH_OH-pi	B3LYP_D3BJ	AcOH_B	-0.0450	-0.0450	-0.0157	-0.0000	0.0293
60_Ethyne-AcOH_OH-pi	M06_2X	Ethyne_A	0.0449	0.0449	0.0130	-0.0000	-0.0319
60_Ethyne-AcOH_OH-pi	M06_2X	AcOH_B	-0.0449	-0.0449	-0.0130	-0.0000	0.0319
60_Ethyne-AcOH_OH-pi	wB97XD	Ethyne_A	0.0443	0.0443	0.0132	0.0000	-0.0311
60_Ethyne-AcOH_OH-pi	wB97XD	AcOH_B	-0.0443	-0.0443	-0.0132	-0.0000	0.0311
61_Pentane-AcOH	B3LYP_D3BJ	Pentane_A	-0.0268	-0.0268	-0.0028	-0.0000	0.0239
61_Pentane-AcOH	B3LYP_D3BJ	AcOH_B	0.0268	0.0268	0.0028	-0.0000	-0.0239
61_Pentane-AcOH	M06_2X	Pentane_A	-0.0272	-0.0272	-0.0027	-0.0000	0.0245
61_Pentane-AcOH	M06_2X	AcOH_B	0.0272	0.0272	0.0027	-0.0000	-0.0245
61_Pentane-AcOH	wB97XD	Pentane_A	-0.0274	-0.0274	-0.0031	-0.0000	0.0242
61_Pentane-AcOH	wB97XD	AcOH_B	0.0274	0.0274	0.0032	-0.0000	-0.0242
62_Pentane-AcNH2	B3LYP_D3BJ	Pentane_A	-0.0428	-0.0428	-0.0047	-0.0000	0.0381
62_Pentane-AcNH2	B3LYP_D3BJ	AcNH2_B	0.0428	0.0428	0.0047	-0.0000	-0.0381
62_Pentane-AcNH2	M06_2X	Pentane_A	-0.0439	-0.0439	-0.0049	-0.0000	0.0390
62_Pentane-AcNH2	M06_2X	AcNH2_B	0.0439	0.0439	0.0049	-0.0000	-0.0390
62_Pentane-AcNH2	wB97XD	Pentane_A	-0.0433	-0.0433	-0.0051	-0.0000	0.0383
62_Pentane-AcNH2	wB97XD	AcNH2_B	0.0433	0.0433	0.0051	-0.0000	-0.0383
63_Benzene-AcOH	B3LYP_D3BJ	Benzene_A	0.0368	0.0368	0.0034	-0.0000	-0.0334
63_Benzene-AcOH	B3LYP_D3BJ	AcOH_B	-0.0368	-0.0368	-0.0034	-0.0000	0.0334
63_Benzene-AcOH	M06_2X	Benzene_A	0.0397	0.0397	0.0027	-0.0000	-0.0370
63_Benzene-AcOH	M06_2X	AcOH_B	-0.0397	-0.0397	-0.0027	-0.0000	0.0370
63_Benzene-AcOH	wB97XD	Benzene_A	0.0369	0.0369	0.0015	-0.0000	-0.0354
63_Benzene-AcOH	wB97XD	AcOH_B	-0.0369	-0.0369	-0.0015	-0.0000	0.0354
64_Peptide-Ethene	B3LYP_D3BJ	Peptide_A	0.0219	0.0219	0.0035	-0.0000	-0.0184
64_Peptide-Ethene	B3LYP_D3BJ	Ethene_B	-0.0219	-0.0219	-0.0035	0.0000	0.0184
64_Peptide-Ethene	M06_2X	Peptide_A	0.0225	0.0225	0.0023	-0.0000	-0.0203
64_Peptide-Ethene	M06_2X	Ethene_B	-0.0225	-0.0225	-0.0023	-0.0000	0.0203
64_Peptide-Ethene	wB97XD	Peptide_A	0.0225	0.0225	0.0030	-0.0000	-0.0195
64_Peptide-Ethene	wB97XD	Ethene_B	-0.0225	-0.0225	-0.0030	-0.0000	0.0195
65_Pyridine-Ethyne	B3LYP_D3BJ	Pyridine_A	0.0796	0.0796	0.0146	-0.0000	-0.0650
65_Pyridine-Ethyne	B3LYP_D3BJ	Ethyne_B	-0.0796	-0.0796	-0.0146	-0.0000	0.0650
65_Pyridine-Ethyne	M06_2X	Pyridine_A	0.0775	0.0775	0.0125	-0.0000	-0.0650
65_Pyridine-Ethyne	M06_2X	Ethyne_B	-0.0775	-0.0775	-0.0125	0.0000	0.0650
65_Pyridine-Ethyne	wB97XD	Pyridine_A	0.0795	0.0795	0.0135	-0.0000	-0.0660
65_Pyridine-Ethyne	wB97XD	Ethyne_B	-0.0795	-0.0795	-0.0135	-0.0000	0.0660
66_MeNH2-Pyridine	B3LYP_D3BJ	MeNH2_A	-0.0603	-0.0603	-0.0062	0.0000	0.0541
66_MeNH2-Pyridine	B3LYP_D3BJ	Pyridine_B	0.0603	0.0603	0.0062	-0.0000	-0.0541
66_MeNH2-Pyridine	M06_2X	MeNH2_A	-0.0622	-0.0622	-0.0049	0.0000	0.0572

Continued on the next page

Table S2: Comparison of Hirshfeld charges obtained from the sum of atomic charges, fragments defined from atomic densities, and fragments defined through WFX files. All calculations were obtained with the 6-311++G** basis set (continued).

System	Functional	Fragment	q_{sum}	q_{atomic}	q_{WFX}	Δ_{atomic}	Δ_{WFX}
66_MeNH2-Pyridine	M06_2X	Pyridine_B	0.0622	0.0622	0.0049	-0.0000	-0.0572
66_MeNH2-Pyridine	wB97XD	MeNH2_A	-0.0613	-0.0613	-0.0051	0.0000	0.0562
66_MeNH2-Pyridine	wB97XD	Pyridine_B	0.0613	0.0613	0.0051	-0.0000	-0.0562

3 CCSD results/6-311++G**

Table S3: Hirshfeld charges obtained using fragments defined from atomic densities and fragments defined through WFX files from CCSD/6-311++G** method.

System	Functional	Fragment	q_{atomic}	q_{WFX}	Δ_{WFX}
01_water_dimer_1	CCSD	cluster_1	-0.0894	-0.0122	0.0772
01_water_dimer_1	CCSD	cluster_2	0.0894	0.0122	-0.0772
02_water_trimer_1	CCSD	cluster_1	-0.0045	-0.0016	0.0030
02_water_trimer_1	CCSD	cluster_2	0.0010	0.0005	-0.0005
02_water_trimer_1	CCSD	cluster_3	0.0036	0.0011	-0.0025
03_water_tetramer_1	CCSD	cluster_1	-0.0000	-0.0000	0.0000
03_water_tetramer_1	CCSD	cluster_2	-0.0000	-0.0000	-0.0000
03_water_tetramer_1	CCSD	cluster_3	-0.0000	-0.0000	-0.0000
03_water_tetramer_1	CCSD	cluster_4	-0.0000	-0.0000	0.0000
04_water_pentamer_1	CCSD	cluster_1	-0.0003	-0.0001	0.0002
04_water_pentamer_1	CCSD	cluster_2	0.0010	0.0004	-0.0005
04_water_pentamer_1	CCSD	cluster_3	0.0035	0.0017	-0.0018
04_water_pentamer_1	CCSD	cluster_4	-0.0042	-0.0022	0.0020
04_water_pentamer_1	CCSD	cluster_5	0.0000	0.0002	0.0002
05_water_hexamer_1	CCSD	cluster_1	-0.0000	-0.0000	0.0000
05_water_hexamer_1	CCSD	cluster_2	-0.0000	-0.0000	0.0000
05_water_hexamer_1	CCSD	cluster_3	-0.0000	-0.0000	-0.0000
05_water_hexamer_1	CCSD	cluster_4	-0.0000	-0.0000	0.0000
05_water_hexamer_1	CCSD	cluster_5	-0.0000	-0.0000	-0.0000
05_water_hexamer_1	CCSD	cluster_6	-0.0000	-0.0000	-0.0000
06_water_hexamer_2	CCSD	cluster_1	0.0047	-0.0123	-0.0170
06_water_hexamer_2	CCSD	cluster_2	-0.0122	0.0124	0.0246
06_water_hexamer_2	CCSD	cluster_3	0.0294	-0.0112	-0.0406
06_water_hexamer_2	CCSD	cluster_4	-0.0503	0.0042	0.0546
06_water_hexamer_2	CCSD	cluster_5	0.0307	-0.0032	-0.0340
06_water_hexamer_2	CCSD	cluster_6	-0.0023	0.0102	0.0124
07_water_hexamer_3	CCSD	cluster_1	0.0351	-0.0047	-0.0399
07_water_hexamer_3	CCSD	cluster_2	-0.0288	0.0061	0.0349
07_water_hexamer_3	CCSD	cluster_3	-0.0365	0.0090	0.0455
07_water_hexamer_3	CCSD	cluster_4	0.0362	-0.0081	-0.0442
07_water_hexamer_3	CCSD	cluster_5	0.0106	0.0043	-0.0063
07_water_hexamer_3	CCSD	cluster_6	-0.0166	-0.0066	0.0100
08_water_hexamer_4	CCSD	cluster_1	0.0486	-0.0032	-0.0518
08_water_hexamer_4	CCSD	cluster_2	0.0045	0.0020	-0.0025
08_water_hexamer_4	CCSD	cluster_3	-0.0033	-0.0021	0.0012
08_water_hexamer_4	CCSD	cluster_4	-0.0058	0.0003	0.0062
08_water_hexamer_4	CCSD	cluster_5	0.0031	-0.0006	-0.0037
08_water_hexamer_4	CCSD	cluster_6	-0.0470	0.0036	0.0506
09_water_hexamer_5	CCSD	cluster_1	-0.0081	-0.0028	0.0053
09_water_hexamer_5	CCSD	cluster_2	-0.0413	0.0153	0.0566
09_water_hexamer_5	CCSD	cluster_3	-0.0081	-0.0028	0.0053
09_water_hexamer_5	CCSD	cluster_4	0.0076	0.0021	-0.0055

Continued on the next page

09_water_hexamer_5	CCSD	cluster_5	0.0422	-0.0140	-0.0562
09_water_hexamer_5	CCSD	cluster_6	0.0076	0.0021	-0.0055
01_Water-Water	CCSD	Water_1	-0.0883	-0.0114	0.0769
01_Water-Water	CCSD	Water_2	0.0883	0.0114	-0.0769
03_Water-MeNH2	CCSD	Water_1	-0.1246	-0.0181	0.1065
03_Water-MeNH2	CCSD	MeNH2_2	0.1246	0.0181	-0.1065
06_MeOH-MeNH2	CCSD	MeOH_1	-0.1310	-0.0213	0.1097
06_MeOH-MeNH2	CCSD	MeNH2_2	0.1310	0.0213	-0.1097
18_Water-Pyridine	CCSD	Water_1	-0.1195	-0.0222	0.0973
18_Water-Pyridine	CCSD	Pyridine_2	0.1195	0.0222	-0.0973
19_MeOH-Pyridine	CCSD	MeOH_1	-0.1299	-0.0251	0.1048
19_MeOH-Pyridine	CCSD	Pyridine_2	0.1299	0.0251	-0.1048

4 Water clusters/6-31G*

Table S4: Comparison of Hirshfeld charges obtained from the sum of atomic charges, fragments defined from atomic densities, and fragments defined through WFX files for water clusters. All calculations were obtained with the 6-31G* basis set.

System	Functional	Fragment	q_{sum}	q_{atomic}	q_{WFX}	Δ_{atomic}	Δ_{WFX}
01_water_dimer_1	B3LYP_D3BJ	cluster_1	-0.0991	-0.0991	-0.0320	0.0000	0.0671
01_water_dimer_1	B3LYP_D3BJ	cluster_2	0.0991	0.0991	0.0320	0.0000	-0.0671
01_water_dimer_1	M06_2X	cluster_1	-0.0953	-0.0953	-0.0263	-0.0000	0.0690
01_water_dimer_1	M06_2X	cluster_2	0.0953	0.0953	0.0263	-0.0000	-0.0690
01_water_dimer_1	wB97XD	cluster_1	-0.0984	-0.0984	-0.0281	-0.0000	0.0703
01_water_dimer_1	wB97XD	cluster_2	0.0984	0.0984	0.0281	-0.0000	-0.0703
02_water_trimer_1	B3LYP_D3BJ	cluster_1	-0.0056	-0.0056	-0.0028	0.0000	0.0028
02_water_trimer_1	B3LYP_D3BJ	cluster_2	0.0017	0.0017	0.0013	-0.0000	-0.0004
02_water_trimer_1	B3LYP_D3BJ	cluster_3	0.0039	0.0039	0.0015	0.0000	-0.0024
02_water_trimer_1	M06_2X	cluster_1	-0.0054	-0.0054	-0.0025	0.0000	0.0029
02_water_trimer_1	M06_2X	cluster_2	0.0014	0.0014	0.0010	0.0000	-0.0004
02_water_trimer_1	M06_2X	cluster_3	0.0039	0.0039	0.0014	0.0000	-0.0025
02_water_trimer_1	wB97XD	cluster_1	-0.0055	-0.0055	-0.0026	-0.0000	0.0029
02_water_trimer_1	wB97XD	cluster_2	0.0015	0.0015	0.0011	-0.0000	-0.0004
02_water_trimer_1	wB97XD	cluster_3	0.0040	0.0040	0.0014	0.0000	-0.0025
03_water_tetramer_1	B3LYP_D3BJ	cluster_1	-0.0000	-0.0000	-0.0000	0.0000	0.0000
03_water_tetramer_1	B3LYP_D3BJ	cluster_2	-0.0000	-0.0000	-0.0000	0.0000	0.0000
03_water_tetramer_1	B3LYP_D3BJ	cluster_3	-0.0000	-0.0000	-0.0000	0.0000	0.0000
03_water_tetramer_1	B3LYP_D3BJ	cluster_4	-0.0000	-0.0000	-0.0000	0.0000	0.0000
03_water_tetramer_1	M06_2X	cluster_1	-0.0000	-0.0000	-0.0000	0.0000	-0.0000
03_water_tetramer_1	M06_2X	cluster_2	-0.0000	-0.0000	-0.0000	0.0000	0.0000
03_water_tetramer_1	M06_2X	cluster_3	-0.0000	-0.0000	-0.0000	0.0000	-0.0000
03_water_tetramer_1	M06_2X	cluster_4	-0.0000	-0.0000	-0.0000	0.0000	0.0000
03_water_tetramer_1	wB97XD	cluster_1	-0.0000	-0.0000	-0.0000	0.0000	0.0000
03_water_tetramer_1	wB97XD	cluster_2	-0.0000	-0.0000	-0.0000	0.0000	0.0000
03_water_tetramer_1	wB97XD	cluster_3	-0.0000	-0.0000	-0.0000	0.0000	0.0000

Continued on the next page

Table S4: Comparison of Hirshfeld charges obtained from the sum of atomic charges, fragments defined from atomic densities, and fragments defined through WFX files for water clusters. All calculations were obtained with the 6-31G* basis set (continued).

System	Functional	Fragment	q_{sum}	q_{atomic}	q_{WFX}	Δ_{atomic}	Δ_{WFX}
03_water_tetramer_1	wB97XD	cluster_4	-0.0000	-0.0000	-0.0000	0.0000	0.0000
04_water_pentamer_1	B3LYP_D3BJ	cluster_1	-0.0005	-0.0005	-0.0002	0.0000	0.0002
04_water_pentamer_1	B3LYP_D3BJ	cluster_2	0.0008	0.0008	0.0005	-0.0000	-0.0003
04_water_pentamer_1	B3LYP_D3BJ	cluster_3	0.0044	0.0044	0.0026	-0.0000	-0.0018
04_water_pentamer_1	B3LYP_D3BJ	cluster_4	-0.0052	-0.0052	-0.0033	0.0000	0.0019
04_water_pentamer_1	B3LYP_D3BJ	cluster_5	0.0005	0.0005	0.0005	-0.0000	-0.0000
04_water_pentamer_1	M06_2X	cluster_1	-0.0004	-0.0004	-0.0002	0.0000	0.0002
04_water_pentamer_1	M06_2X	cluster_2	0.0008	0.0008	0.0004	0.0000	-0.0004
04_water_pentamer_1	M06_2X	cluster_3	0.0044	0.0044	0.0025	0.0000	-0.0019
04_water_pentamer_1	M06_2X	cluster_4	-0.0051	-0.0051	-0.0031	0.0000	0.0020
04_water_pentamer_1	M06_2X	cluster_5	0.0004	0.0004	0.0004	-0.0000	-0.0000
04_water_pentamer_1	wB97XD	cluster_1	-0.0004	-0.0004	-0.0002	0.0000	0.0002
04_water_pentamer_1	wB97XD	cluster_2	0.0008	0.0008	0.0004	0.0000	-0.0004
04_water_pentamer_1	wB97XD	cluster_3	0.0043	0.0043	0.0025	0.0000	-0.0019
04_water_pentamer_1	wB97XD	cluster_4	-0.0051	-0.0051	-0.0031	0.0000	0.0020
04_water_pentamer_1	wB97XD	cluster_5	0.0004	0.0004	0.0004	-0.0000	-0.0000
05_water_hexamer_1	B3LYP_D3BJ	cluster_1	-0.0000	-0.0000	-0.0000	-0.0000	0.0000
05_water_hexamer_1	B3LYP_D3BJ	cluster_2	0.0000	0.0000	0.0000	0.0000	-0.0000
05_water_hexamer_1	B3LYP_D3BJ	cluster_3	0.0000	0.0000	0.0000	0.0000	-0.0000
05_water_hexamer_1	B3LYP_D3BJ	cluster_4	-0.0000	-0.0000	-0.0000	-0.0000	0.0000
05_water_hexamer_1	B3LYP_D3BJ	cluster_5	0.0000	0.0000	0.0000	0.0000	-0.0000
05_water_hexamer_1	B3LYP_D3BJ	cluster_6	0.0000	0.0000	0.0000	0.0000	-0.0000
05_water_hexamer_1	M06_2X	cluster_1	-0.0000	-0.0000	0.0000	0.0000	0.0000
05_water_hexamer_1	M06_2X	cluster_2	0.0000	0.0000	-0.0000	0.0000	-0.0000
05_water_hexamer_1	M06_2X	cluster_3	-0.0000	-0.0000	-0.0000	0.0000	0.0000
05_water_hexamer_1	M06_2X	cluster_4	-0.0000	-0.0000	0.0000	0.0000	0.0000
05_water_hexamer_1	M06_2X	cluster_5	0.0000	0.0000	-0.0000	0.0000	-0.0000
05_water_hexamer_1	M06_2X	cluster_6	-0.0000	-0.0000	-0.0000	-0.0000	0.0000
05_water_hexamer_1	wB97XD	cluster_1	-0.0000	-0.0000	-0.0000	-0.0000	0.0000
05_water_hexamer_1	wB97XD	cluster_2	-0.0000	-0.0000	-0.0000	-0.0000	-0.0000
05_water_hexamer_1	wB97XD	cluster_3	0.0000	0.0000	0.0000	0.0000	-0.0000
05_water_hexamer_1	wB97XD	cluster_4	-0.0000	-0.0000	-0.0000	-0.0000	0.0000
05_water_hexamer_1	wB97XD	cluster_5	-0.0000	-0.0000	-0.0000	0.0000	-0.0000
05_water_hexamer_1	wB97XD	cluster_6	0.0000	0.0000	0.0000	0.0000	-0.0000
06_water_hexamer_2	B3LYP_D3BJ	cluster_1	0.0050	0.0050	-0.0099	0.0000	-0.0148
06_water_hexamer_2	B3LYP_D3BJ	cluster_2	-0.0181	-0.0181	0.0046	0.0000	0.0227
06_water_hexamer_2	B3LYP_D3BJ	cluster_3	0.0342	0.0342	-0.0018	0.0000	-0.0360
06_water_hexamer_2	B3LYP_D3BJ	cluster_4	-0.0527	-0.0527	-0.0067	0.0000	0.0460
06_water_hexamer_2	B3LYP_D3BJ	cluster_5	0.0349	0.0349	0.0052	0.0000	-0.0298
06_water_hexamer_2	B3LYP_D3BJ	cluster_6	-0.0032	-0.0032	0.0086	-0.0000	0.0119
06_water_hexamer_2	M06_2X	cluster_1	0.0048	0.0048	-0.0104	0.0000	-0.0152
06_water_hexamer_2	M06_2X	cluster_2	-0.0156	-0.0156	0.0075	0.0000	0.0231
06_water_hexamer_2	M06_2X	cluster_3	0.0322	0.0322	-0.0047	0.0000	-0.0369
06_water_hexamer_2	M06_2X	cluster_4	-0.0518	-0.0518	-0.0044	0.0000	0.0474

Continued on the next page

Table S4: Comparison of Hirshfeld charges obtained from the sum of atomic charges, fragments defined from atomic densities, and fragments defined through WFX files for water clusters. All calculations were obtained with the 6-31G* basis set (continued).

System	Functional	Fragment	q_{sum}	q_{atomic}	q_{WFX}	Δ_{atomic}	Δ_{WFX}
06_water_hexamer_2	M06_2X	cluster_5	0.0338	0.0338	0.0030	0.0000	-0.0308
06_water_hexamer_2	M06_2X	cluster_6	-0.0034	-0.0034	0.0091	-0.0000	0.0125
06_water_hexamer_2	wB97XD	cluster_1	0.0057	0.0057	-0.0102	0.0000	-0.0159
06_water_hexamer_2	wB97XD	cluster_2	-0.0182	-0.0182	0.0059	-0.0000	0.0240
06_water_hexamer_2	wB97XD	cluster_3	0.0349	0.0349	-0.0031	0.0000	-0.0380
06_water_hexamer_2	wB97XD	cluster_4	-0.0535	-0.0535	-0.0051	-0.0000	0.0483
06_water_hexamer_2	wB97XD	cluster_5	0.0351	0.0351	0.0036	0.0000	-0.0314
06_water_hexamer_2	wB97XD	cluster_6	-0.0040	-0.0040	0.0089	-0.0000	0.0129
07_water_hexamer_3	B3LYP_D3BJ	cluster_1	0.0405	0.0405	0.0053	-0.0000	-0.0352
07_water_hexamer_3	B3LYP_D3BJ	cluster_2	-0.0326	-0.0326	-0.0019	-0.0000	0.0307
07_water_hexamer_3	B3LYP_D3BJ	cluster_3	-0.0382	-0.0382	0.0010	-0.0000	0.0392
07_water_hexamer_3	B3LYP_D3BJ	cluster_4	0.0397	0.0397	0.0016	-0.0000	-0.0381
07_water_hexamer_3	B3LYP_D3BJ	cluster_5	0.0119	0.0119	0.0069	-0.0000	-0.0051
07_water_hexamer_3	B3LYP_D3BJ	cluster_6	-0.0213	-0.0213	-0.0128	-0.0000	0.0085
07_water_hexamer_3	M06_2X	cluster_1	0.0393	0.0393	0.0028	0.0000	-0.0365
07_water_hexamer_3	M06_2X	cluster_2	-0.0320	-0.0320	0.0000	0.0000	0.0320
07_water_hexamer_3	M06_2X	cluster_3	-0.0364	-0.0364	0.0038	-0.0000	0.0402
07_water_hexamer_3	M06_2X	cluster_4	0.0376	0.0376	-0.0014	-0.0000	-0.0390
07_water_hexamer_3	M06_2X	cluster_5	0.0113	0.0113	0.0061	0.0000	-0.0052
07_water_hexamer_3	M06_2X	cluster_6	-0.0199	-0.0199	-0.0112	-0.0000	0.0086
07_water_hexamer_3	wB97XD	cluster_1	0.0409	0.0409	0.0037	-0.0000	-0.0373
07_water_hexamer_3	wB97XD	cluster_2	-0.0333	-0.0333	-0.0007	0.0000	0.0326
07_water_hexamer_3	wB97XD	cluster_3	-0.0391	-0.0391	0.0023	-0.0000	0.0414
07_water_hexamer_3	wB97XD	cluster_4	0.0401	0.0401	0.0000	0.0000	-0.0401
07_water_hexamer_3	wB97XD	cluster_5	0.0116	0.0116	0.0063	-0.0000	-0.0052
07_water_hexamer_3	wB97XD	cluster_6	-0.0202	-0.0202	-0.0117	0.0000	0.0085
08_water_hexamer_4	B3LYP_D3BJ	cluster_1	0.0562	0.0562	0.0106	-0.0000	-0.0456
08_water_hexamer_4	B3LYP_D3BJ	cluster_2	0.0045	0.0045	0.0024	0.0000	-0.0021
08_water_hexamer_4	B3LYP_D3BJ	cluster_3	-0.0044	-0.0044	-0.0031	-0.0000	0.0014
08_water_hexamer_4	B3LYP_D3BJ	cluster_4	-0.0045	-0.0045	-0.0001	0.0000	0.0044
08_water_hexamer_4	B3LYP_D3BJ	cluster_5	0.0033	0.0033	0.0007	0.0000	-0.0026
08_water_hexamer_4	B3LYP_D3BJ	cluster_6	-0.0551	-0.0551	-0.0106	-0.0000	0.0445
08_water_hexamer_4	M06_2X	cluster_1	0.0538	0.0538	0.0069	0.0000	-0.0470
08_water_hexamer_4	M06_2X	cluster_2	0.0045	0.0045	0.0023	0.0000	-0.0022
08_water_hexamer_4	M06_2X	cluster_3	-0.0039	-0.0039	-0.0026	-0.0000	0.0014
08_water_hexamer_4	M06_2X	cluster_4	-0.0046	-0.0046	-0.0001	0.0000	0.0045
08_water_hexamer_4	M06_2X	cluster_5	0.0030	0.0030	0.0004	0.0000	-0.0026
08_water_hexamer_4	M06_2X	cluster_6	-0.0529	-0.0529	-0.0069	0.0000	0.0459
08_water_hexamer_4	wB97XD	cluster_1	0.0563	0.0563	0.0084	0.0000	-0.0479
08_water_hexamer_4	wB97XD	cluster_2	0.0045	0.0045	0.0023	0.0000	-0.0022
08_water_hexamer_4	wB97XD	cluster_3	-0.0040	-0.0040	-0.0026	0.0000	0.0014
08_water_hexamer_4	wB97XD	cluster_4	-0.0046	-0.0046	-0.0001	-0.0000	0.0045
08_water_hexamer_4	wB97XD	cluster_5	0.0032	0.0032	0.0006	-0.0000	-0.0027
08_water_hexamer_4	wB97XD	cluster_6	-0.0555	-0.0555	-0.0086	0.0000	0.0469

Continued on the next page

Table S4: Comparison of Hirshfeld charges obtained from the sum of atomic charges, fragments defined from atomic densities, and fragments defined through WFX files for water clusters. All calculations were obtained with the 6-31G* basis set (continued).

System	Functional	Fragment	q_{sum}	q_{atomic}	q_{WFX}	Δ_{atomic}	Δ_{WFX}
09_water_hexamer_5	B3LYP_D3BJ	cluster_1	-0.0089	-0.0089	-0.0043	-0.0000	0.0046
09_water_hexamer_5	B3LYP_D3BJ	cluster_2	-0.0480	-0.0480	0.0015	0.0000	0.0495
09_water_hexamer_5	B3LYP_D3BJ	cluster_3	-0.0089	-0.0089	-0.0043	-0.0000	0.0046
09_water_hexamer_5	B3LYP_D3BJ	cluster_4	0.0083	0.0083	0.0031	-0.0000	-0.0052
09_water_hexamer_5	B3LYP_D3BJ	cluster_5	0.0492	0.0492	0.0010	-0.0000	-0.0481
09_water_hexamer_5	B3LYP_D3BJ	cluster_6	0.0083	0.0083	0.0031	-0.0000	-0.0052
09_water_hexamer_5	M06_2X	cluster_1	-0.0086	-0.0086	-0.0038	0.0000	0.0047
09_water_hexamer_5	M06_2X	cluster_2	-0.0444	-0.0444	0.0058	0.0000	0.0502
09_water_hexamer_5	M06_2X	cluster_3	-0.0086	-0.0086	-0.0038	0.0000	0.0047
09_water_hexamer_5	M06_2X	cluster_4	0.0081	0.0081	0.0028	-0.0000	-0.0053
09_water_hexamer_5	M06_2X	cluster_5	0.0453	0.0453	-0.0038	-0.0000	-0.0491
09_water_hexamer_5	M06_2X	cluster_6	0.0081	0.0081	0.0028	-0.0000	-0.0053
09_water_hexamer_5	wB97XD	cluster_1	-0.0087	-0.0087	-0.0039	0.0000	0.0047
09_water_hexamer_5	wB97XD	cluster_2	-0.0487	-0.0487	0.0032	0.0000	0.0519
09_water_hexamer_5	wB97XD	cluster_3	-0.0087	-0.0087	-0.0039	0.0000	0.0047
09_water_hexamer_5	wB97XD	cluster_4	0.0083	0.0083	0.0029	-0.0000	-0.0054
09_water_hexamer_5	wB97XD	cluster_5	0.0494	0.0494	-0.0011	-0.0000	-0.0505
09_water_hexamer_5	wB97XD	cluster_6	0.0083	0.0083	0.0029	-0.0000	-0.0054

5 S66 set/6-31G*

Table S5: Comparison of Hirshfeld charges obtained from the sum of atomic charges, fragments defined from atomic densities, and fragments defined through WFX files. All calculations were obtained with the 6-31G* basis set.

System	Functional	Fragment	q_{sum}	q_{atomic}	q_{WFX}	Δ_{atomic}	Δ_{WFX}
01_Water-Water	B3LYP_D3BJ	Water_A	-0.0974	-0.0974	-0.0304	-0.0000	0.0670
01_Water-Water	B3LYP_D3BJ	Water_B	0.0974	0.0974	0.0304	0.0000	-0.0670
01_Water-Water	M06_2X	Water_A	-0.0939	-0.0939	-0.0250	-0.0000	0.0689
01_Water-Water	M06_2X	Water_B	0.0939	0.0939	0.0250	-0.0000	-0.0689
01_Water-Water	wB97XD	Water_A	-0.0968	-0.0968	-0.0267	-0.0000	0.0701
01_Water-Water	wB97XD	Water_B	0.0968	0.0968	0.0267	0.0000	-0.0701
02_Water-MeOH	B3LYP_D3BJ	MeOH_B	0.0996	0.0996	0.0322	0.0000	-0.0674
02_Water-MeOH	B3LYP_D3BJ	Water_A	-0.0996	-0.0996	-0.0322	0.0000	0.0674
02_Water-MeOH	M06_2X	MeOH_B	0.0963	0.0963	0.0266	-0.0000	-0.0697
02_Water-MeOH	M06_2X	Water_A	-0.0963	-0.0963	-0.0266	-0.0000	0.0697
02_Water-MeOH	wB97XD	MeOH_B	0.0993	0.0993	0.0284	-0.0000	-0.0709
02_Water-MeOH	wB97XD	Water_A	-0.0993	-0.0993	-0.0284	0.0000	0.0709
03_Water-MeNH2	B3LYP_D3BJ	MeNH2_B	0.1364	0.1364	0.0422	0.0000	-0.0941
03_Water-MeNH2	B3LYP_D3BJ	Water_A	-0.1364	-0.1364	-0.0422	-0.0000	0.0941
03_Water-MeNH2	M06_2X	MeNH2_B	0.1328	0.1328	0.0351	0.0000	-0.0977

Continued on the next page

Table S5: Comparison of Hirshfeld charges obtained from the sum of atomic charges, fragments defined from atomic densities, and fragments defined through WFX files. All calculations were obtained with the 6-31G* basis set (continued).

System	Functional	Fragment	q_{sum}	q_{atomic}	q_{WFX}	Δ_{atomic}	Δ_{WFX}
03_Water-MeNH2	M06_2X	Water_A	-0.1328	-0.1328	-0.0351	0.0000	0.0977
03_Water-MeNH2	wB97XD	MeNH2_B	0.1352	0.1352	0.0367	0.0000	-0.0985
03_Water-MeNH2	wB97XD	Water_A	-0.1352	-0.1352	-0.0367	-0.0000	0.0985
04_Water-Peptide	B3LYP_D3BJ	Peptide_B	0.1048	0.1048	0.0363	-0.0000	-0.0686
04_Water-Peptide	B3LYP_D3BJ	Water_A	-0.1048	-0.1048	-0.0363	-0.0000	0.0686
04_Water-Peptide	M06_2X	Peptide_B	0.1013	0.1013	0.0305	-0.0000	-0.0709
04_Water-Peptide	M06_2X	Water_A	-0.1013	-0.1013	-0.0305	-0.0000	0.0709
04_Water-Peptide	wB97XD	Peptide_B	0.1043	0.1043	0.0323	0.0000	-0.0720
04_Water-Peptide	wB97XD	Water_A	-0.1043	-0.1043	-0.0323	-0.0000	0.0720
05_MeOH-MeOH	B3LYP_D3BJ	MeOH_A	-0.1032	-0.1032	-0.0340	0.0000	0.0692
05_MeOH-MeOH	B3LYP_D3BJ	MeOH_B	0.1032	0.1032	0.0340	0.0000	-0.0692
05_MeOH-MeOH	M06_2X	MeOH_A	-0.1000	-0.1000	-0.0285	-0.0000	0.0716
05_MeOH-MeOH	M06_2X	MeOH_B	0.1000	0.1000	0.0285	0.0000	-0.0716
05_MeOH-MeOH	wB97XD	MeOH_A	-0.1029	-0.1029	-0.0302	-0.0000	0.0728
05_MeOH-MeOH	wB97XD	MeOH_B	0.1029	0.1029	0.0302	0.0000	-0.0728
06_MeOH-MeNH2	B3LYP_D3BJ	MeNH2_B	0.1413	0.1413	0.0447	-0.0000	-0.0966
06_MeOH-MeNH2	B3LYP_D3BJ	MeOH_A	-0.1413	-0.1413	-0.0447	0.0000	0.0966
06_MeOH-MeNH2	M06_2X	MeNH2_B	0.1385	0.1385	0.0380	-0.0000	-0.1005
06_MeOH-MeNH2	M06_2X	MeOH_A	-0.1385	-0.1385	-0.0380	-0.0000	0.1005
06_MeOH-MeNH2	wB97XD	MeNH2_B	0.1405	0.1405	0.0394	-0.0000	-0.1011
06_MeOH-MeNH2	wB97XD	MeOH_A	-0.1405	-0.1405	-0.0394	0.0000	0.1011
07_MeOH-Peptide	B3LYP_D3BJ	MeOH_A	-0.1003	-0.1003	-0.0331	0.0000	0.0672
07_MeOH-Peptide	B3LYP_D3BJ	Peptide_B	0.1003	0.1003	0.0330	-0.0000	-0.0672
07_MeOH-Peptide	M06_2X	MeOH_A	-0.0979	-0.0979	-0.0286	0.0000	0.0694
07_MeOH-Peptide	M06_2X	Peptide_B	0.0979	0.0979	0.0286	0.0000	-0.0694
07_MeOH-Peptide	wB97XD	MeOH_A	-0.1002	-0.1002	-0.0298	-0.0000	0.0704
07_MeOH-Peptide	wB97XD	Peptide_B	0.1002	0.1002	0.0298	0.0000	-0.0704
08_MeOH-Water	B3LYP_D3BJ	MeOH_A	-0.0998	-0.0998	-0.0314	0.0000	0.0683
08_MeOH-Water	B3LYP_D3BJ	Water_B	0.0998	0.0998	0.0314	0.0000	-0.0683
08_MeOH-Water	M06_2X	MeOH_A	-0.0965	-0.0965	-0.0262	0.0000	0.0703
08_MeOH-Water	M06_2X	Water_B	0.0965	0.0965	0.0262	0.0000	-0.0703
08_MeOH-Water	wB97XD	MeOH_A	-0.0993	-0.0993	-0.0277	0.0000	0.0716
08_MeOH-Water	wB97XD	Water_B	0.0993	0.0993	0.0277	0.0000	-0.0716
09_MeNH2-MeOH	B3LYP_D3BJ	MeNH2_A	-0.0600	-0.0600	-0.0212	0.0000	0.0388
09_MeNH2-MeOH	B3LYP_D3BJ	MeOH_B	0.0600	0.0600	0.0212	-0.0000	-0.0388
09_MeNH2-MeOH	M06_2X	MeNH2_A	-0.0572	-0.0572	-0.0173	0.0000	0.0399
09_MeNH2-MeOH	M06_2X	MeOH_B	0.0572	0.0572	0.0173	0.0000	-0.0399
09_MeNH2-MeOH	wB97XD	MeNH2_A	-0.0596	-0.0596	-0.0187	-0.0000	0.0409
09_MeNH2-MeOH	wB97XD	MeOH_B	0.0596	0.0596	0.0187	-0.0000	-0.0409
10_MeNH2-MeNH2	B3LYP_D3BJ	MeNH2_A	-0.0623	-0.0623	-0.0191	0.0000	0.0432
10_MeNH2-MeNH2	B3LYP_D3BJ	MeNH2_B	0.0623	0.0623	0.0191	-0.0000	-0.0432
10_MeNH2-MeNH2	M06_2X	MeNH2_A	-0.0605	-0.0605	-0.0156	0.0000	0.0449
10_MeNH2-MeNH2	M06_2X	MeNH2_B	0.0605	0.0605	0.0156	-0.0000	-0.0449
10_MeNH2-MeNH2	wB97XD	MeNH2_A	-0.0620	-0.0620	-0.0165	0.0000	0.0456

Continued on the next page

Table S5: Comparison of Hirshfeld charges obtained from the sum of atomic charges, fragments defined from atomic densities, and fragments defined through WFX files. All calculations were obtained with the 6-31G* basis set (continued).

System	Functional	Fragment	q_{sum}	q_{atomic}	q_{WFX}	Δ_{atomic}	Δ_{WFX}
10_MeNH2-MeNH2	wB97XD	MeNH2_B	0.0620	0.0620	0.0165	-0.0000	-0.0456
11_MeNH2-Peptide	B3LYP_D3BJ	MeNH2_A	-0.0288	-0.0288	-0.0079	-0.0000	0.0209
11_MeNH2-Peptide	B3LYP_D3BJ	Peptide_B	0.0288	0.0288	0.0079	0.0000	-0.0209
11_MeNH2-Peptide	M06_2X	MeNH2_A	-0.0277	-0.0277	-0.0065	0.0000	0.0212
11_MeNH2-Peptide	M06_2X	Peptide_B	0.0277	0.0277	0.0065	-0.0000	-0.0212
11_MeNH2-Peptide	wB97XD	MeNH2_A	-0.0285	-0.0285	-0.0070	0.0000	0.0215
11_MeNH2-Peptide	wB97XD	Peptide_B	0.0285	0.0285	0.0070	0.0000	-0.0215
12_MeNH2-Water	B3LYP_D3BJ	MeNH2_A	0.1306	0.1306	0.0393	0.0000	-0.0912
12_MeNH2-Water	B3LYP_D3BJ	Water_B	-0.1306	-0.1306	-0.0393	-0.0000	0.0912
12_MeNH2-Water	M06_2X	MeNH2_A	0.1276	0.1276	0.0328	-0.0000	-0.0948
12_MeNH2-Water	M06_2X	Water_B	-0.1276	-0.1276	-0.0328	-0.0000	0.0948
12_MeNH2-Water	wB97XD	MeNH2_A	0.1300	0.1300	0.0343	-0.0000	-0.0956
12_MeNH2-Water	wB97XD	Water_B	-0.1300	-0.1300	-0.0343	0.0000	0.0956
13_Peptide-MeOH	B3LYP_D3BJ	MeOH_B	0.0861	0.0861	0.0289	0.0000	-0.0572
13_Peptide-MeOH	B3LYP_D3BJ	Peptide_A	-0.0861	-0.0861	-0.0289	-0.0000	0.0572
13_Peptide-MeOH	M06_2X	MeOH_B	0.0833	0.0833	0.0242	0.0000	-0.0591
13_Peptide-MeOH	M06_2X	Peptide_A	-0.0833	-0.0833	-0.0242	-0.0000	0.0591
13_Peptide-MeOH	wB97XD	MeOH_B	0.0865	0.0865	0.0260	-0.0000	-0.0605
13_Peptide-MeOH	wB97XD	Peptide_A	-0.0865	-0.0865	-0.0260	0.0000	0.0605
14_Peptide-MeNH2	B3LYP_D3BJ	MeNH2_B	0.1161	0.1161	0.0382	0.0000	-0.0779
14_Peptide-MeNH2	B3LYP_D3BJ	Peptide_A	-0.1161	-0.1161	-0.0382	-0.0000	0.0779
14_Peptide-MeNH2	M06_2X	MeNH2_B	0.1133	0.1133	0.0321	-0.0000	-0.0812
14_Peptide-MeNH2	M06_2X	Peptide_A	-0.1133	-0.1133	-0.0321	0.0000	0.0812
14_Peptide-MeNH2	wB97XD	MeNH2_B	0.1160	0.1160	0.0339	0.0000	-0.0821
14_Peptide-MeNH2	wB97XD	Peptide_A	-0.1160	-0.1160	-0.0339	0.0000	0.0821
15_Peptide-Peptide	B3LYP_D3BJ	Peptide_A	-0.0986	-0.0986	-0.0383	0.0000	0.0603
15_Peptide-Peptide	B3LYP_D3BJ	Peptide_B	0.0986	0.0986	0.0383	-0.0000	-0.0603
15_Peptide-Peptide	M06_2X	Peptide_A	-0.0946	-0.0946	-0.0329	0.0000	0.0617
15_Peptide-Peptide	M06_2X	Peptide_B	0.0946	0.0946	0.0329	0.0000	-0.0617
15_Peptide-Peptide	wB97XD	Peptide_A	-0.0981	-0.0981	-0.0350	-0.0000	0.0631
15_Peptide-Peptide	wB97XD	Peptide_B	0.0981	0.0981	0.0350	-0.0000	-0.0631
16_Peptide-Water	B3LYP_D3BJ	Peptide_A	-0.0869	-0.0869	-0.0281	0.0000	0.0588
16_Peptide-Water	B3LYP_D3BJ	Water_B	0.0869	0.0869	0.0281	0.0000	-0.0588
16_Peptide-Water	M06_2X	Peptide_A	-0.0840	-0.0840	-0.0234	-0.0000	0.0606
16_Peptide-Water	M06_2X	Water_B	0.0840	0.0840	0.0234	0.0000	-0.0606
16_Peptide-Water	wB97XD	Peptide_A	-0.0869	-0.0869	-0.0249	0.0000	0.0620
16_Peptide-Water	wB97XD	Water_B	0.0869	0.0869	0.0249	0.0000	-0.0620
17_Uracil-Uracil_BP	B3LYP_D3BJ	Uracil_A	0.0165	0.0165	0.0080	0.0000	-0.0085
17_Uracil-Uracil_BP	B3LYP_D3BJ	Uracil_B	-0.0165	-0.0165	-0.0080	-0.0000	0.0085
17_Uracil-Uracil_BP	M06_2X	Uracil_A	0.0157	0.0157	0.0074	0.0000	-0.0083
17_Uracil-Uracil_BP	M06_2X	Uracil_B	-0.0157	-0.0157	-0.0074	-0.0000	0.0083
17_Uracil-Uracil_BP	wB97XD	Uracil_A	0.0161	0.0161	0.0076	0.0000	-0.0085
17_Uracil-Uracil_BP	wB97XD	Uracil_B	-0.0161	-0.0161	-0.0076	-0.0000	0.0085
18_Water-Pyridine	B3LYP_D3BJ	Pyridine_B	0.1268	0.1268	0.0403	-0.0000	-0.0865

Continued on the next page

Table S5: Comparison of Hirshfeld charges obtained from the sum of atomic charges, fragments defined from atomic densities, and fragments defined through WFX files. All calculations were obtained with the 6-31G* basis set (continued).

System	Functional	Fragment	q_{sum}	q_{atomic}	q_{WFX}	Δ_{atomic}	Δ_{WFX}
18_Water-Pyridine	B3LYP_D3BJ	Water_A	-0.1268	-0.1268	-0.0403	-0.0000	0.0865
18_Water-Pyridine	M06_2X	Pyridine_B	0.1234	0.1234	0.0338	0.0000	-0.0896
18_Water-Pyridine	M06_2X	Water_A	-0.1234	-0.1234	-0.0338	-0.0000	0.0896
18_Water-Pyridine	wB97XD	Pyridine_B	0.1261	0.1261	0.0353	0.0000	-0.0908
18_Water-Pyridine	wB97XD	Water_A	-0.1261	-0.1261	-0.0353	0.0000	0.0908
19_MeOH-Pyridine	B3LYP_D3BJ	MeOH_A	-0.1351	-0.1351	-0.0446	0.0000	0.0905
19_MeOH-Pyridine	B3LYP_D3BJ	Pyridine_B	0.1351	0.1351	0.0446	-0.0000	-0.0905
19_MeOH-Pyridine	M06_2X	MeOH_A	-0.1318	-0.1318	-0.0381	0.0000	0.0937
19_MeOH-Pyridine	M06_2X	Pyridine_B	0.1319	0.1319	0.0381	0.0000	-0.0937
19_MeOH-Pyridine	wB97XD	MeOH_A	-0.1343	-0.1343	-0.0394	-0.0000	0.0948
19_MeOH-Pyridine	wB97XD	Pyridine_B	0.1343	0.1343	0.0394	-0.0000	-0.0948
20_AcOH-AcOH	B3LYP_D3BJ	AcOH_A	0.0000	0.0000	0.0000	0.0000	0.0000
20_AcOH-AcOH	B3LYP_D3BJ	AcOH_B	-0.0000	-0.0000	-0.0000	0.0000	-0.0000
20_AcOH-AcOH	M06_2X	AcOH_A	0.0000	0.0000	0.0000	0.0000	0.0000
20_AcOH-AcOH	M06_2X	AcOH_B	-0.0000	-0.0000	-0.0000	0.0000	-0.0000
20_AcOH-AcOH	wB97XD	AcOH_A	0.0000	0.0000	0.0000	0.0000	0.0000
20_AcOH-AcOH	wB97XD	AcOH_B	-0.0000	-0.0000	-0.0000	0.0000	-0.0000
21_AcNH2-AcNH2	B3LYP_D3BJ	AcNH2_A	-0.0000	-0.0000	-0.0000	0.0000	0.0000
21_AcNH2-AcNH2	B3LYP_D3BJ	AcNH2_B	0.0000	0.0000	0.0000	0.0000	-0.0000
21_AcNH2-AcNH2	M06_2X	AcNH2_A	-0.0000	-0.0000	-0.0000	0.0000	0.0000
21_AcNH2-AcNH2	M06_2X	AcNH2_B	0.0000	0.0000	0.0000	-0.0000	-0.0000
21_AcNH2-AcNH2	wB97XD	AcNH2_A	-0.0000	-0.0000	-0.0000	0.0000	0.0000
21_AcNH2-AcNH2	wB97XD	AcNH2_B	0.0000	0.0000	0.0000	-0.0000	-0.0000
22_AcOH-Uracil	B3LYP_D3BJ	AcOH_A	-0.0192	-0.0192	-0.0080	0.0000	0.0112
22_AcOH-Uracil	B3LYP_D3BJ	Uracil_B	0.0192	0.0192	0.0080	0.0000	-0.0112
22_AcOH-Uracil	M06_2X	AcOH_A	-0.0199	-0.0199	-0.0073	-0.0000	0.0127
22_AcOH-Uracil	M06_2X	Uracil_B	0.0199	0.0199	0.0073	-0.0000	-0.0127
22_AcOH-Uracil	wB97XD	AcOH_A	-0.0192	-0.0192	-0.0070	-0.0000	0.0122
22_AcOH-Uracil	wB97XD	Uracil_B	0.0192	0.0192	0.0070	-0.0000	-0.0122
23_AcNH2-Uracil	B3LYP_D3BJ	AcNH2_A	0.0455	0.0455	0.0228	0.0000	-0.0227
23_AcNH2-Uracil	B3LYP_D3BJ	Uracil_B	-0.0455	-0.0455	-0.0228	0.0000	0.0227
23_AcNH2-Uracil	M06_2X	AcNH2_A	0.0451	0.0451	0.0217	0.0000	-0.0234
23_AcNH2-Uracil	M06_2X	Uracil_B	-0.0451	-0.0451	-0.0217	-0.0000	0.0234
23_AcNH2-Uracil	wB97XD	AcNH2_A	0.0451	0.0451	0.0218	0.0000	-0.0234
23_AcNH2-Uracil	wB97XD	Uracil_B	-0.0451	-0.0451	-0.0218	-0.0000	0.0234
24_Benzene-Benzene_pi-pi	B3LYP_D3BJ	Benzene_A	0.0000	0.0000	0.0000	-0.0000	-0.0000
24_Benzene-Benzene_pi-pi	B3LYP_D3BJ	Benzene_B	-0.0000	-0.0000	-0.0000	-0.0000	0.0000
24_Benzene-Benzene_pi-pi	M06_2X	Benzene_A	0.0000	0.0000	0.0000	0.0000	-0.0000
24_Benzene-Benzene_pi-pi	M06_2X	Benzene_B	-0.0000	-0.0000	-0.0000	0.0000	0.0000
24_Benzene-Benzene_pi-pi	wB97XD	Benzene_A	0.0000	0.0000	0.0000	-0.0000	-0.0000
24_Benzene-Benzene_pi-pi	wB97XD	Benzene_B	-0.0000	-0.0000	-0.0000	0.0000	0.0000
25_Pyridine-Pyridine_pi-pi	B3LYP_D3BJ	Pyridine_A	-0.0003	-0.0003	-0.0001	-0.0000	0.0001
25_Pyridine-Pyridine_pi-pi	B3LYP_D3BJ	Pyridine_B	0.0002	0.0002	0.0001	-0.0000	-0.0001
25_Pyridine-Pyridine_pi-pi	M06_2X	Pyridine_A	-0.0002	-0.0002	-0.0001	-0.0000	0.0001

Continued on the next page

Table S5: Comparison of Hirshfeld charges obtained from the sum of atomic charges, fragments defined from atomic densities, and fragments defined through WFX files. All calculations were obtained with the 6-31G* basis set (continued).

System	Functional	Fragment	q_{sum}	q_{atomic}	q_{WFX}	Δ_{atomic}	Δ_{WFX}
25_Pyridine-Pyridine_pi-pi	M06_2X	Pyridine_B	0.0002	0.0002	0.0001	-0.0000	-0.0001
25_Pyridine-Pyridine_pi-pi	wB97XD	Pyridine_A	-0.0002	-0.0002	-0.0001	0.0000	0.0001
25_Pyridine-Pyridine_pi-pi	wB97XD	Pyridine_B	0.0002	0.0002	0.0001	-0.0000	-0.0001
26_Uracil-Uracil_pi-pi	B3LYP_D3BJ	Uracil_A	-0.0000	-0.0000	-0.0000	0.0000	-0.0000
26_Uracil-Uracil_pi-pi	B3LYP_D3BJ	Uracil_B	-0.0000	-0.0000	-0.0000	-0.0000	0.0000
26_Uracil-Uracil_pi-pi	M06_2X	Uracil_A	-0.0000	-0.0000	-0.0000	-0.0000	-0.0000
26_Uracil-Uracil_pi-pi	M06_2X	Uracil_B	-0.0000	-0.0000	-0.0000	0.0000	0.0000
26_Uracil-Uracil_pi-pi	wB97XD	Uracil_A	-0.0000	-0.0000	-0.0000	-0.0000	-0.0000
26_Uracil-Uracil_pi-pi	wB97XD	Uracil_B	-0.0000	-0.0000	-0.0000	0.0000	0.0000
27_Benzene-Pyridine_pi-pi	B3LYP_D3BJ	Benzene_A	0.0062	0.0062	0.0035	-0.0000	-0.0026
27_Benzene-Pyridine_pi-pi	B3LYP_D3BJ	Pyridine_B	-0.0061	-0.0061	-0.0035	0.0000	0.0026
27_Benzene-Pyridine_pi-pi	M06_2X	Benzene_A	0.0059	0.0059	0.0030	0.0000	-0.0029
27_Benzene-Pyridine_pi-pi	M06_2X	Pyridine_B	-0.0059	-0.0059	-0.0030	-0.0000	0.0029
27_Benzene-Pyridine_pi-pi	wB97XD	Benzene_A	0.0057	0.0057	0.0029	0.0000	-0.0028
27_Benzene-Pyridine_pi-pi	wB97XD	Pyridine_B	-0.0057	-0.0057	-0.0029	-0.0000	0.0028
28_Benzene-Uracil_pi-pi	B3LYP_D3BJ	Benzene_A	0.0160	0.0160	0.0055	0.0000	-0.0105
28_Benzene-Uracil_pi-pi	B3LYP_D3BJ	Uracil_B	-0.0160	-0.0160	-0.0055	0.0000	0.0105
28_Benzene-Uracil_pi-pi	M06_2X	Benzene_A	0.0178	0.0178	0.0056	-0.0000	-0.0122
28_Benzene-Uracil_pi-pi	M06_2X	Uracil_B	-0.0178	-0.0178	-0.0056	0.0000	0.0122
28_Benzene-Uracil_pi-pi	wB97XD	Benzene_A	0.0165	0.0165	0.0046	-0.0000	-0.0119
28_Benzene-Uracil_pi-pi	wB97XD	Uracil_B	-0.0165	-0.0165	-0.0046	-0.0000	0.0119
29_Pyridine-Uracil_pi-pi	B3LYP_D3BJ	Pyridine_A	0.0055	0.0055	0.0001	-0.0000	-0.0054
29_Pyridine-Uracil_pi-pi	B3LYP_D3BJ	Uracil_B	-0.0055	-0.0055	-0.0001	-0.0000	0.0054
29_Pyridine-Uracil_pi-pi	M06_2X	Pyridine_A	0.0074	0.0074	0.0011	-0.0000	-0.0063
29_Pyridine-Uracil_pi-pi	M06_2X	Uracil_B	-0.0074	-0.0074	-0.0011	0.0000	0.0063
29_Pyridine-Uracil_pi-pi	wB97XD	Pyridine_A	0.0067	0.0067	0.0005	-0.0000	-0.0063
29_Pyridine-Uracil_pi-pi	wB97XD	Uracil_B	-0.0068	-0.0068	-0.0005	-0.0000	0.0063
30_Benzene-Ethene	B3LYP_D3BJ	Benzene_A	0.0139	0.0139	0.0059	-0.0000	-0.0080
30_Benzene-Ethene	B3LYP_D3BJ	Ethene_B	-0.0139	-0.0139	-0.0059	0.0000	0.0080
30_Benzene-Ethene	M06_2X	Benzene_A	0.0134	0.0134	0.0046	-0.0000	-0.0087
30_Benzene-Ethene	M06_2X	Ethene_B	-0.0134	-0.0134	-0.0047	0.0000	0.0087
30_Benzene-Ethene	wB97XD	Benzene_A	0.0131	0.0131	0.0046	0.0000	-0.0086
30_Benzene-Ethene	wB97XD	Ethene_B	-0.0132	-0.0132	-0.0046	-0.0000	0.0086
31_Uracil-Ethene	B3LYP_D3BJ	Ethene_B	-0.0096	-0.0096	-0.0013	-0.0000	0.0083
31_Uracil-Ethene	B3LYP_D3BJ	Uracil_A	0.0096	0.0096	0.0013	-0.0000	-0.0083
31_Uracil-Ethene	M06_2X	Ethene_B	-0.0083	-0.0083	0.0003	0.0000	0.0087
31_Uracil-Ethene	M06_2X	Uracil_A	0.0083	0.0083	-0.0003	0.0000	-0.0087
31_Uracil-Ethene	wB97XD	Ethene_B	-0.0089	-0.0089	-0.0003	-0.0000	0.0086
31_Uracil-Ethene	wB97XD	Uracil_A	0.0089	0.0089	0.0003	0.0000	-0.0086
32_Uracil-Ethyne	B3LYP_D3BJ	Ethyne_B	-0.0018	-0.0018	0.0006	-0.0000	0.0023
32_Uracil-Ethyne	B3LYP_D3BJ	Uracil_A	0.0017	0.0017	-0.0006	-0.0000	-0.0023
32_Uracil-Ethyne	M06_2X	Ethyne_B	-0.0007	-0.0007	0.0018	0.0000	0.0025
32_Uracil-Ethyne	M06_2X	Uracil_A	0.0007	0.0007	-0.0018	0.0000	-0.0025
32_Uracil-Ethyne	wB97XD	Ethyne_B	-0.0011	-0.0011	0.0012	-0.0000	0.0022

Continued on the next page

Table S5: Comparison of Hirshfeld charges obtained from the sum of atomic charges, fragments defined from atomic densities, and fragments defined through WFX files. All calculations were obtained with the 6-31G* basis set (continued).

System	Functional	Fragment	q_{sum}	q_{atomic}	q_{WFX}	Δ_{atomic}	Δ_{WFX}
32_Uracil-Ethyne	wB97XD	Uracil_A	0.0011	0.0011	-0.0012	0.0000	-0.0022
33_Pyridine-Ethene	B3LYP_D3BJ	Ethene_B	-0.0108	-0.0108	-0.0042	0.0000	0.0065
33_Pyridine-Ethene	B3LYP_D3BJ	Pyridine_A	0.0107	0.0107	0.0042	-0.0000	-0.0065
33_Pyridine-Ethene	M06_2X	Ethene_B	-0.0103	-0.0103	-0.0031	0.0000	0.0072
33_Pyridine-Ethene	M06_2X	Pyridine_A	0.0103	0.0103	0.0031	-0.0000	-0.0072
33_Pyridine-Ethene	wB97XD	Ethene_B	-0.0102	-0.0102	-0.0032	0.0000	0.0070
33_Pyridine-Ethene	wB97XD	Pyridine_A	0.0102	0.0102	0.0031	0.0000	-0.0070
34_Pentane-Pentane	B3LYP_D3BJ	Pentane_A	-0.0000	-0.0000	-0.0000	0.0000	-0.0000
34_Pentane-Pentane	B3LYP_D3BJ	Pentane_B	-0.0000	-0.0000	-0.0000	0.0000	0.0000
34_Pentane-Pentane	M06_2X	Pentane_A	-0.0000	-0.0000	-0.0000	0.0000	-0.0000
34_Pentane-Pentane	M06_2X	Pentane_B	-0.0000	-0.0000	-0.0000	0.0000	0.0000
34_Pentane-Pentane	wB97XD	Pentane_A	-0.0000	-0.0000	-0.0000	-0.0000	-0.0000
34_Pentane-Pentane	wB97XD	Pentane_B	-0.0000	-0.0000	-0.0000	-0.0000	0.0000
35_Neopentane-Pentane	B3LYP_D3BJ	Neopentane_A	0.0003	0.0003	0.0005	0.0000	0.0002
35_Neopentane-Pentane	B3LYP_D3BJ	Pentane_B	-0.0003	-0.0003	-0.0005	-0.0000	-0.0002
35_Neopentane-Pentane	M06_2X	Neopentane_A	0.0002	0.0002	-0.0003	0.0000	-0.0005
35_Neopentane-Pentane	M06_2X	Pentane_B	-0.0003	-0.0003	0.0002	-0.0000	0.0005
35_Neopentane-Pentane	wB97XD	Neopentane_A	0.0006	0.0006	-0.0001	-0.0000	-0.0007
35_Neopentane-Pentane	wB97XD	Pentane_B	-0.0007	-0.0007	0.0001	0.0000	0.0007
36_Neopentane-Neopentane	B3LYP_D3BJ	Neopentane_A	0.0000	0.0000	0.0000	0.0000	0.0000
36_Neopentane-Neopentane	B3LYP_D3BJ	Neopentane_B	0.0000	0.0000	0.0000	-0.0000	-0.0000
36_Neopentane-Neopentane	M06_2X	Neopentane_A	0.0000	0.0000	0.0000	-0.0000	0.0000
36_Neopentane-Neopentane	M06_2X	Neopentane_B	0.0000	0.0000	0.0000	-0.0000	-0.0000
36_Neopentane-Neopentane	wB97XD	Neopentane_A	0.0000	0.0000	0.0000	-0.0000	0.0000
36_Neopentane-Neopentane	wB97XD	Neopentane_B	0.0000	0.0000	0.0000	0.0000	-0.0000
37_Cyclopentane-Neopentane	B3LYP_D3BJ	Cyclopentane_A	-0.0023	-0.0023	-0.0005	-0.0000	0.0018
37_Cyclopentane-Neopentane	B3LYP_D3BJ	Neopentane_B	0.0023	0.0023	0.0005	-0.0000	-0.0018
37_Cyclopentane-Neopentane	M06_2X	Cyclopentane_A	-0.0022	-0.0022	-0.0005	0.0000	0.0017
37_Cyclopentane-Neopentane	M06_2X	Neopentane_B	0.0022	0.0022	0.0005	-0.0000	-0.0017
37_Cyclopentane-Neopentane	wB97XD	Cyclopentane_A	-0.0020	-0.0020	-0.0005	-0.0000	0.0015
37_Cyclopentane-Neopentane	wB97XD	Neopentane_B	0.0020	0.0020	0.0005	-0.0000	-0.0015
38_Cyclopentane-Cyclopentane	B3LYP_D3BJ	Cyclopentane_A	-0.0000	-0.0000	-0.0000	-0.0000	-0.0000
38_Cyclopentane-Cyclopentane	B3LYP_D3BJ	Cyclopentane_B	-0.0000	-0.0000	-0.0000	-0.0000	0.0000
38_Cyclopentane-Cyclopentane	M06_2X	Cyclopentane_A	-0.0000	-0.0000	-0.0000	-0.0000	-0.0000
38_Cyclopentane-Cyclopentane	M06_2X	Cyclopentane_B	-0.0000	-0.0000	-0.0000	-0.0000	0.0000
38_Cyclopentane-Cyclopentane	wB97XD	Cyclopentane_A	0.0000	0.0000	0.0000	-0.0000	-0.0000
38_Cyclopentane-Cyclopentane	wB97XD	Cyclopentane_B	-0.0000	-0.0000	-0.0000	0.0000	0.0000
39_Benzene-Cyclopentane	B3LYP_D3BJ	Benzene_A	0.0572	0.0572	0.0184	0.0000	-0.0388
39_Benzene-Cyclopentane	B3LYP_D3BJ	Cyclopentane_B	-0.0572	-0.0572	-0.0184	-0.0000	0.0388
39_Benzene-Cyclopentane	M06_2X	Benzene_A	0.0577	0.0577	0.0153	-0.0000	-0.0424
39_Benzene-Cyclopentane	M06_2X	Cyclopentane_B	-0.0577	-0.0577	-0.0153	0.0000	0.0424
39_Benzene-Cyclopentane	wB97XD	Benzene_A	0.0573	0.0573	0.0147	0.0000	-0.0425
39_Benzene-Cyclopentane	wB97XD	Cyclopentane_B	-0.0573	-0.0573	-0.0147	0.0000	0.0425
40_Benzene-Neopentane	B3LYP_D3BJ	Benzene_A	0.0512	0.0512	0.0183	0.0000	-0.0329

Continued on the next page

Table S5: Comparison of Hirshfeld charges obtained from the sum of atomic charges, fragments defined from atomic densities, and fragments defined through WFX files. All calculations were obtained with the 6-31G* basis set (continued).

System	Functional	Fragment	q_{sum}	q_{atomic}	q_{WFX}	Δ_{atomic}	Δ_{WFX}
40_Benzene-Neopentane	B3LYP_D3BJ	Neopentane_B	-0.0512	-0.0512	-0.0183	-0.0000	0.0329
40_Benzene-Neopentane	M06_2X	Benzene_A	0.0513	0.0513	0.0151	0.0000	-0.0362
40_Benzene-Neopentane	M06_2X	Neopentane_B	-0.0513	-0.0513	-0.0151	0.0000	0.0362
40_Benzene-Neopentane	wB97XD	Benzene_A	0.0512	0.0512	0.0148	-0.0000	-0.0364
40_Benzene-Neopentane	wB97XD	Neopentane_B	-0.0512	-0.0512	-0.0148	0.0000	0.0364
41_Uracil-Pentane	B3LYP_D3BJ	Pentane_B	-0.0605	-0.0605	-0.0178	-0.0000	0.0427
41_Uracil-Pentane	B3LYP_D3BJ	Uracil_A	0.0605	0.0605	0.0178	-0.0000	-0.0427
41_Uracil-Pentane	M06_2X	Pentane_B	-0.0592	-0.0592	-0.0138	0.0000	0.0454
41_Uracil-Pentane	M06_2X	Uracil_A	0.0592	0.0592	0.0138	0.0000	-0.0454
41_Uracil-Pentane	wB97XD	Pentane_B	-0.0604	-0.0604	-0.0145	-0.0000	0.0459
41_Uracil-Pentane	wB97XD	Uracil_A	0.0603	0.0603	0.0145	-0.0000	-0.0459
42_Uracil-Cyclopentane	B3LYP_D3BJ	Cyclopentane_B	-0.0534	-0.0534	-0.0132	0.0000	0.0402
42_Uracil-Cyclopentane	B3LYP_D3BJ	Uracil_A	0.0534	0.0534	0.0132	0.0000	-0.0402
42_Uracil-Cyclopentane	M06_2X	Cyclopentane_B	-0.0525	-0.0525	-0.0094	-0.0000	0.0431
42_Uracil-Cyclopentane	M06_2X	Uracil_A	0.0525	0.0525	0.0094	0.0000	-0.0431
42_Uracil-Cyclopentane	wB97XD	Cyclopentane_B	-0.0536	-0.0536	-0.0102	-0.0000	0.0434
42_Uracil-Cyclopentane	wB97XD	Uracil_A	0.0536	0.0536	0.0102	0.0000	-0.0434
43_Uracil-Neopentane	B3LYP_D3BJ	Neopentane_B	-0.0365	-0.0365	-0.0101	-0.0000	0.0264
43_Uracil-Neopentane	B3LYP_D3BJ	Uracil_A	0.0365	0.0365	0.0101	0.0000	-0.0264
43_Uracil-Neopentane	M06_2X	Neopentane_B	-0.0353	-0.0353	-0.0074	-0.0000	0.0278
43_Uracil-Neopentane	M06_2X	Uracil_A	0.0353	0.0353	0.0075	-0.0000	-0.0278
43_Uracil-Neopentane	wB97XD	Neopentane_B	-0.0362	-0.0362	-0.0081	0.0000	0.0281
43_Uracil-Neopentane	wB97XD	Uracil_A	0.0362	0.0362	0.0081	-0.0000	-0.0281
44_Ethene-Pentane	B3LYP_D3BJ	Ethene_A	0.0049	0.0049	0.0031	-0.0000	-0.0018
44_Ethene-Pentane	B3LYP_D3BJ	Pentane_B	-0.0049	-0.0049	-0.0031	0.0000	0.0018
44_Ethene-Pentane	M06_2X	Ethene_A	0.0047	0.0047	0.0035	-0.0000	-0.0012
44_Ethene-Pentane	M06_2X	Pentane_B	-0.0047	-0.0047	-0.0035	0.0000	0.0012
44_Ethene-Pentane	wB97XD	Ethene_A	0.0045	0.0045	0.0031	0.0000	-0.0014
44_Ethene-Pentane	wB97XD	Pentane_B	-0.0046	-0.0046	-0.0031	0.0000	0.0014
45_Ethyne-Pentane	B3LYP_D3BJ	Ethyne_A	0.0313	0.0313	0.0115	-0.0000	-0.0198
45_Ethyne-Pentane	B3LYP_D3BJ	Pentane_B	-0.0313	-0.0313	-0.0116	0.0000	0.0198
45_Ethyne-Pentane	M06_2X	Ethyne_A	0.0308	0.0308	0.0104	-0.0000	-0.0205
45_Ethyne-Pentane	M06_2X	Pentane_B	-0.0308	-0.0308	-0.0104	0.0000	0.0205
45_Ethyne-Pentane	wB97XD	Ethyne_A	0.0307	0.0307	0.0098	0.0000	-0.0209
45_Ethyne-Pentane	wB97XD	Pentane_B	-0.0308	-0.0308	-0.0098	0.0000	0.0209
46_Peptide-Pentane	B3LYP_D3BJ	Pentane_B	-0.0353	-0.0353	-0.0135	-0.0000	0.0217
46_Peptide-Pentane	B3LYP_D3BJ	Peptide_A	0.0352	0.0352	0.0135	0.0000	-0.0217
46_Peptide-Pentane	M06_2X	Pentane_B	-0.0343	-0.0343	-0.0116	-0.0000	0.0227
46_Peptide-Pentane	M06_2X	Peptide_A	0.0343	0.0343	0.0116	-0.0000	-0.0227
46_Peptide-Pentane	wB97XD	Pentane_B	-0.0353	-0.0353	-0.0121	0.0000	0.0232
46_Peptide-Pentane	wB97XD	Peptide_A	0.0353	0.0353	0.0120	0.0000	-0.0232
47_Benzene-Benzene_TS	B3LYP_D3BJ	Benzene_A	0.0491	0.0491	0.0153	-0.0000	-0.0338
47_Benzene-Benzene_TS	B3LYP_D3BJ	Benzene_B	-0.0491	-0.0491	-0.0153	-0.0000	0.0338
47_Benzene-Benzene_TS	M06_2X	Benzene_A	0.0497	0.0497	0.0121	0.0000	-0.0376

Continued on the next page

Table S5: Comparison of Hirshfeld charges obtained from the sum of atomic charges, fragments defined from atomic densities, and fragments defined through WFX files. All calculations were obtained with the 6-31G* basis set (continued).

System	Functional	Fragment	q_{sum}	q_{atomic}	q_{WFX}	Δ_{atomic}	Δ_{WFX}
47_Benzene-Benzene_TS	M06_2X	Benzene_B	-0.0497	-0.0497	-0.0121	-0.0000	0.0376
47_Benzene-Benzene_TS	wB97XD	Benzene_A	0.0496	0.0496	0.0119	0.0000	-0.0377
47_Benzene-Benzene_TS	wB97XD	Benzene_B	-0.0496	-0.0496	-0.0119	0.0000	0.0377
48_Pyridine-Pyridine_TS	B3LYP_D3BJ	Pyridine_A	0.0588	0.0588	0.0174	0.0000	-0.0414
48_Pyridine-Pyridine_TS	B3LYP_D3BJ	Pyridine_B	-0.0588	-0.0588	-0.0174	0.0000	0.0414
48_Pyridine-Pyridine_TS	M06_2X	Pyridine_A	0.0587	0.0587	0.0137	-0.0000	-0.0450
48_Pyridine-Pyridine_TS	M06_2X	Pyridine_B	-0.0587	-0.0587	-0.0137	0.0000	0.0450
48_Pyridine-Pyridine_TS	wB97XD	Pyridine_A	0.0595	0.0595	0.0141	0.0000	-0.0454
48_Pyridine-Pyridine_TS	wB97XD	Pyridine_B	-0.0596	-0.0596	-0.0141	-0.0000	0.0454
49_Benzene-Pyridine_TS	B3LYP_D3BJ	Benzene_A	0.0542	0.0542	0.0172	0.0000	-0.0370
49_Benzene-Pyridine_TS	B3LYP_D3BJ	Pyridine_B	-0.0542	-0.0542	-0.0172	-0.0000	0.0370
49_Benzene-Pyridine_TS	M06_2X	Benzene_A	0.0548	0.0548	0.0137	-0.0000	-0.0411
49_Benzene-Pyridine_TS	M06_2X	Pyridine_B	-0.0548	-0.0548	-0.0137	0.0000	0.0411
49_Benzene-Pyridine_TS	wB97XD	Benzene_A	0.0547	0.0547	0.0135	0.0000	-0.0412
49_Benzene-Pyridine_TS	wB97XD	Pyridine_B	-0.0547	-0.0547	-0.0135	-0.0000	0.0412
50_Benzene-Ethyne_CH-pi	B3LYP_D3BJ	Benzene_A	0.0550	0.0550	0.0144	-0.0000	-0.0406
50_Benzene-Ethyne_CH-pi	B3LYP_D3BJ	Ethyne_B	-0.0551	-0.0551	-0.0144	0.0000	0.0406
50_Benzene-Ethyne_CH-pi	M06_2X	Benzene_A	0.0548	0.0548	0.0109	-0.0000	-0.0438
50_Benzene-Ethyne_CH-pi	M06_2X	Ethyne_B	-0.0548	-0.0548	-0.0109	0.0000	0.0438
50_Benzene-Ethyne_CH-pi	wB97XD	Benzene_A	0.0546	0.0546	0.0107	0.0000	-0.0440
50_Benzene-Ethyne_CH-pi	wB97XD	Ethyne_B	-0.0546	-0.0546	-0.0107	0.0000	0.0440
51_Ethyne-Ethyne_TS	B3LYP_D3BJ	Ethyne_A	0.0405	0.0405	0.0143	-0.0000	-0.0262
51_Ethyne-Ethyne_TS	B3LYP_D3BJ	Ethyne_B	-0.0405	-0.0405	-0.0143	0.0000	0.0262
51_Ethyne-Ethyne_TS	M06_2X	Ethyne_A	0.0387	0.0387	0.0114	-0.0000	-0.0273
51_Ethyne-Ethyne_TS	M06_2X	Ethyne_B	-0.0387	-0.0387	-0.0114	0.0000	0.0273
51_Ethyne-Ethyne_TS	wB97XD	Ethyne_A	0.0399	0.0399	0.0118	0.0000	-0.0281
51_Ethyne-Ethyne_TS	wB97XD	Ethyne_B	-0.0399	-0.0399	-0.0118	0.0000	0.0281
52_Benzene-AcOH_OH-pi	B3LYP_D3BJ	AcOH_B	-0.0738	-0.0738	-0.0188	-0.0000	0.0550
52_Benzene-AcOH_OH-pi	B3LYP_D3BJ	Benzene_A	0.0739	0.0739	0.0188	-0.0000	-0.0550
52_Benzene-AcOH_OH-pi	M06_2X	AcOH_B	-0.0751	-0.0751	-0.0149	0.0000	0.0602
52_Benzene-AcOH_OH-pi	M06_2X	Benzene_A	0.0751	0.0751	0.0149	0.0000	-0.0602
52_Benzene-AcOH_OH-pi	wB97XD	AcOH_B	-0.0744	-0.0744	-0.0147	-0.0000	0.0597
52_Benzene-AcOH_OH-pi	wB97XD	Benzene_A	0.0744	0.0744	0.0147	-0.0000	-0.0597
53_Benzene-AcNH2_NH-pi	B3LYP_D3BJ	AcNH2_B	-0.0195	-0.0195	-0.0032	-0.0000	0.0164
53_Benzene-AcNH2_NH-pi	B3LYP_D3BJ	Benzene_A	0.0195	0.0195	0.0032	-0.0000	-0.0164
53_Benzene-AcNH2_NH-pi	M06_2X	AcNH2_B	-0.0210	-0.0210	-0.0019	-0.0000	0.0191
53_Benzene-AcNH2_NH-pi	M06_2X	Benzene_A	0.0210	0.0210	0.0019	-0.0000	-0.0191
53_Benzene-AcNH2_NH-pi	wB97XD	AcNH2_B	-0.0199	-0.0199	-0.0014	-0.0000	0.0185
53_Benzene-AcNH2_NH-pi	wB97XD	Benzene_A	0.0199	0.0199	0.0014	-0.0000	-0.0185
54_Benzene-Water_OH-pi	B3LYP_D3BJ	Benzene_A	0.0676	0.0676	0.0140	-0.0000	-0.0536
54_Benzene-Water_OH-pi	B3LYP_D3BJ	Water_B	-0.0676	-0.0676	-0.0140	-0.0000	0.0536
54_Benzene-Water_OH-pi	M06_2X	Benzene_A	0.0681	0.0681	0.0106	-0.0000	-0.0575
54_Benzene-Water_OH-pi	M06_2X	Water_B	-0.0681	-0.0681	-0.0106	-0.0000	0.0575
54_Benzene-Water_OH-pi	wB97XD	Benzene_A	0.0678	0.0678	0.0105	0.0000	-0.0573

Continued on the next page

Table S5: Comparison of Hirshfeld charges obtained from the sum of atomic charges, fragments defined from atomic densities, and fragments defined through WFX files. All calculations were obtained with the 6-31G* basis set (continued).

System	Functional	Fragment	q_{sum}	q_{atomic}	q_{WFX}	Δ_{atomic}	Δ_{WFX}
54_Benzene-Water_OH-pi	wB97XD	Water_B	-0.0678	-0.0678	-0.0105	-0.0000	0.0573
55_Benzene-MeOH_OH-pi	B3LYP_D3BJ	Benzene_A	0.0812	0.0812	0.0185	-0.0000	-0.0627
55_Benzene-MeOH_OH-pi	B3LYP_D3BJ	MeOH_B	-0.0812	-0.0812	-0.0185	0.0000	0.0627
55_Benzene-MeOH_OH-pi	M06_2X	Benzene_A	0.0822	0.0822	0.0150	0.0000	-0.0673
55_Benzene-MeOH_OH-pi	M06_2X	MeOH_B	-0.0822	-0.0822	-0.0150	0.0000	0.0673
55_Benzene-MeOH_OH-pi	wB97XD	Benzene_A	0.0814	0.0814	0.0146	-0.0000	-0.0668
55_Benzene-MeOH_OH-pi	wB97XD	MeOH_B	-0.0814	-0.0814	-0.0146	0.0000	0.0668
56_Benzene-MeNH2_NH-pi	B3LYP_D3BJ	Benzene_A	0.0608	0.0608	0.0143	-0.0000	-0.0465
56_Benzene-MeNH2_NH-pi	B3LYP_D3BJ	MeNH2_B	-0.0608	-0.0608	-0.0143	0.0000	0.0465
56_Benzene-MeNH2_NH-pi	M06_2X	Benzene_A	0.0615	0.0615	0.0113	-0.0000	-0.0502
56_Benzene-MeNH2_NH-pi	M06_2X	MeNH2_B	-0.0615	-0.0615	-0.0113	-0.0000	0.0502
56_Benzene-MeNH2_NH-pi	wB97XD	Benzene_A	0.0611	0.0611	0.0111	-0.0000	-0.0500
56_Benzene-MeNH2_NH-pi	wB97XD	MeNH2_B	-0.0611	-0.0611	-0.0111	-0.0000	0.0500
57_Benzene-Peptide_NH-pi	B3LYP_D3BJ	Benzene_A	0.0821	0.0821	0.0232	0.0000	-0.0589
57_Benzene-Peptide_NH-pi	B3LYP_D3BJ	Peptide_B	-0.0821	-0.0821	-0.0232	0.0000	0.0589
57_Benzene-Peptide_NH-pi	M06_2X	Benzene_A	0.0831	0.0831	0.0192	-0.0000	-0.0639
57_Benzene-Peptide_NH-pi	M06_2X	Peptide_B	-0.0831	-0.0831	-0.0192	-0.0000	0.0639
57_Benzene-Peptide_NH-pi	wB97XD	Benzene_A	0.0823	0.0823	0.0188	0.0000	-0.0635
57_Benzene-Peptide_NH-pi	wB97XD	Peptide_B	-0.0823	-0.0823	-0.0188	-0.0000	0.0635
58_Pyridine-Pyridine_CH-N	B3LYP_D3BJ	Pyridine_A	-0.0000	-0.0000	-0.0000	-0.0000	0.0000
58_Pyridine-Pyridine_CH-N	B3LYP_D3BJ	Pyridine_B	0.0000	0.0000	0.0000	-0.0000	-0.0000
58_Pyridine-Pyridine_CH-N	M06_2X	Pyridine_A	-0.0000	-0.0000	-0.0000	-0.0000	0.0000
58_Pyridine-Pyridine_CH-N	M06_2X	Pyridine_B	0.0000	0.0000	0.0000	-0.0000	-0.0000
58_Pyridine-Pyridine_CH-N	wB97XD	Pyridine_A	-0.0000	-0.0000	-0.0000	0.0000	0.0000
58_Pyridine-Pyridine_CH-N	wB97XD	Pyridine_B	0.0000	0.0000	0.0000	-0.0000	-0.0000
59_Ethyne-Water_CH-O	B3LYP_D3BJ	Ethyne_A	-0.0581	-0.0581	-0.0182	-0.0000	0.0399
59_Ethyne-Water_CH-O	B3LYP_D3BJ	Water_B	0.0581	0.0581	0.0182	0.0000	-0.0399
59_Ethyne-Water_CH-O	M06_2X	Ethyne_A	-0.0557	-0.0557	-0.0148	-0.0000	0.0409
59_Ethyne-Water_CH-O	M06_2X	Water_B	0.0557	0.0557	0.0148	-0.0000	-0.0409
59_Ethyne-Water_CH-O	wB97XD	Ethyne_A	-0.0582	-0.0582	-0.0159	0.0000	0.0423
59_Ethyne-Water_CH-O	wB97XD	Water_B	0.0582	0.0582	0.0159	0.0000	-0.0423
60_Ethyne-AcOH_OH-pi	B3LYP_D3BJ	AcOH_B	-0.0509	-0.0509	-0.0188	0.0000	0.0322
60_Ethyne-AcOH_OH-pi	B3LYP_D3BJ	Ethyne_A	0.0509	0.0509	0.0188	-0.0000	-0.0322
60_Ethyne-AcOH_OH-pi	M06_2X	AcOH_B	-0.0511	-0.0511	-0.0158	0.0000	0.0353
60_Ethyne-AcOH_OH-pi	M06_2X	Ethyne_A	0.0511	0.0511	0.0158	-0.0000	-0.0353
60_Ethyne-AcOH_OH-pi	wB97XD	AcOH_B	-0.0507	-0.0507	-0.0157	-0.0000	0.0350
60_Ethyne-AcOH_OH-pi	wB97XD	Ethyne_A	0.0507	0.0507	0.0157	-0.0000	-0.0350
61_Pentane-AcOH	B3LYP_D3BJ	AcOH_B	0.0267	0.0267	0.0107	-0.0000	-0.0160
61_Pentane-AcOH	B3LYP_D3BJ	Pentane_A	-0.0267	-0.0267	-0.0107	0.0000	0.0160
61_Pentane-AcOH	M06_2X	AcOH_B	0.0251	0.0251	0.0089	-0.0000	-0.0162
61_Pentane-AcOH	M06_2X	Pentane_A	-0.0251	-0.0251	-0.0089	0.0000	0.0162
61_Pentane-AcOH	wB97XD	AcOH_B	0.0266	0.0266	0.0096	-0.0000	-0.0170
61_Pentane-AcOH	wB97XD	Pentane_A	-0.0266	-0.0266	-0.0096	0.0000	0.0170
62_Pentane-AcNH2	B3LYP_D3BJ	AcNH2_B	0.0410	0.0410	0.0185	-0.0000	-0.0225

Continued on the next page

Table S5: Comparison of Hirshfeld charges obtained from the sum of atomic charges, fragments defined from atomic densities, and fragments defined through WFX files. All calculations were obtained with the 6-31G* basis set (continued).

System	Functional	Fragment	q_{sum}	q_{atomic}	q_{WFX}	Δ_{atomic}	Δ_{WFX}
62_Pentane-AcNH2	B3LYP_D3BJ	Pentane_A	-0.0410	-0.0410	-0.0185	0.0000	0.0225
62_Pentane-AcNH2	M06_2X	AcNH2_B	0.0392	0.0392	0.0163	-0.0000	-0.0229
62_Pentane-AcNH2	M06_2X	Pentane_A	-0.0392	-0.0392	-0.0163	-0.0000	0.0229
62_Pentane-AcNH2	wB97XD	AcNH2_B	0.0404	0.0404	0.0168	-0.0000	-0.0236
62_Pentane-AcNH2	wB97XD	Pentane_A	-0.0404	-0.0404	-0.0168	-0.0000	0.0236
63_Benzene-AcOH	B3LYP_D3BJ	AcOH_B	-0.0424	-0.0424	-0.0122	0.0000	0.0302
63_Benzene-AcOH	B3LYP_D3BJ	Benzene_A	0.0424	0.0424	0.0122	0.0000	-0.0302
63_Benzene-AcOH	M06_2X	AcOH_B	-0.0433	-0.0433	-0.0105	0.0000	0.0328
63_Benzene-AcOH	M06_2X	Benzene_A	0.0433	0.0433	0.0105	-0.0000	-0.0328
63_Benzene-AcOH	wB97XD	AcOH_B	-0.0424	-0.0424	-0.0099	-0.0000	0.0325
63_Benzene-AcOH	wB97XD	Benzene_A	0.0424	0.0424	0.0099	-0.0000	-0.0325
64_Peptide-Ethene	B3LYP_D3BJ	Ethene_B	-0.0218	-0.0218	-0.0061	-0.0000	0.0157
64_Peptide-Ethene	B3LYP_D3BJ	Peptide_A	0.0218	0.0218	0.0061	0.0000	-0.0157
64_Peptide-Ethene	M06_2X	Ethene_B	-0.0211	-0.0211	-0.0045	0.0000	0.0166
64_Peptide-Ethene	M06_2X	Peptide_A	0.0211	0.0211	0.0045	-0.0000	-0.0166
64_Peptide-Ethene	wB97XD	Ethene_B	-0.0221	-0.0221	-0.0053	-0.0000	0.0169
64_Peptide-Ethene	wB97XD	Peptide_A	0.0221	0.0221	0.0053	-0.0000	-0.0169
65_Pyridine-Ethyne	B3LYP_D3BJ	Ethyne_B	-0.0806	-0.0806	-0.0276	-0.0000	0.0530
65_Pyridine-Ethyne	B3LYP_D3BJ	Pyridine_A	0.0806	0.0806	0.0276	0.0000	-0.0530
65_Pyridine-Ethyne	M06_2X	Ethyne_B	-0.0768	-0.0768	-0.0224	0.0000	0.0544
65_Pyridine-Ethyne	M06_2X	Pyridine_A	0.0768	0.0768	0.0224	0.0000	-0.0544
65_Pyridine-Ethyne	wB97XD	Ethyne_B	-0.0802	-0.0802	-0.0240	0.0000	0.0561
65_Pyridine-Ethyne	wB97XD	Pyridine_A	0.0802	0.0802	0.0241	0.0000	-0.0561
66_MeNH2-Pyridine	B3LYP_D3BJ	MeNH2_A	-0.0649	-0.0649	-0.0163	-0.0000	0.0486
66_MeNH2-Pyridine	B3LYP_D3BJ	Pyridine_B	0.0649	0.0649	0.0163	0.0000	-0.0486
66_MeNH2-Pyridine	M06_2X	MeNH2_A	-0.0640	-0.0640	-0.0133	0.0000	0.0507
66_MeNH2-Pyridine	M06_2X	Pyridine_B	0.0641	0.0641	0.0133	0.0000	-0.0507
66_MeNH2-Pyridine	wB97XD	MeNH2_A	-0.0650	-0.0650	-0.0138	0.0000	0.0512
66_MeNH2-Pyridine	wB97XD	Pyridine_B	0.0650	0.0650	0.0138	-0.0000	-0.0512
